# A randomized non-inferiority trial of therapeutic strategy with immunosuppressants versus biologics for Vogt-Koyanagi-Harada disease

**DOI:** 10.1038/s41467-023-39483-5

**Published:** 2023-06-24

**Authors:** Zhenyu Zhong, Lingyu Dai, Qiuying Wu, Yu Gao, Yanlin Pu, Guannan Su, Xiaorong Lu, Fuxiang Zhang, Chong Tang, Yao Wang, Chunjiang Zhou, Peizeng Yang

**Affiliations:** The First Affiliated Hospital of Chongqing Medical University, Chongqing Key Laboratory of Ophthalmology, Chongqing Eye Institute, and Chongqing Branch (Municipality Division) of National Clinical Research Center for Ocular Diseases, Chongqing, China

**Keywords:** Uveal diseases, Biological therapy, Translational research, Immunotherapy

## Abstract

Biologics are increasingly used to treat Vogt-Koyanagi-Harada disease, but head-to-head comparisons with conventional immunosuppressants are lacking. Here in this randomized trial (Chinese Clinical Trial Registry, ChiCTR2100043061), we assigned 110 patients (27 early-phase and 83 late-phase) to cyclosporine-based immunosuppressant strategy (*N* = 56) or adalimumab-based biologic strategy (*N* = 54), each combined with a modified corticosteroid regimen. The primary outcome is change from baseline in best-corrected visual acuity at week 26. The margin of non-inferiority for cyclosporine is −7 letters. The primary outcome is 11.2 letters (95% CI, 7.5 to 14.9) in the cyclosporine group and 6.3 letters (95% CI, 3.1 to 9.6) in the adalimumab group (difference, 4.9; 95% CI, 0.2 to 9.5; *P* < 0.001 for non-inferiority). The between-group difference is −0.8 letters (95% CI, −6.1 to 4.5) in early-phase disease and 5.7 letters (95% CI, 0.2 to 11.2) in late-phase. Serious adverse events are reported less frequently in the cyclosporine group than in the adalimumab group (0.70 vs. 1.21 events per patient-year). Here, we report that combined with a non-standard corticosteroid regimen, cyclosporine-based immunosuppressant strategy is non-inferior to adalimumab-based biologic strategy by 26 weeks for visual improvement in a cohort of patients with Vogt-Koyanagi-Harada disease, 75% of whom have a late-phase disease.

## Abstract (web summary)

Different classes of biologic therapeutics have been assessed in the context of Vogt-Koyanagi-Harada disease. Here the authors compared cyclosporine immunosuppression or adalimumab in a randomised clinical trial for the treatment of patients with Vogt-Koyanagi-Harada disease and found non-inferiority upon treatment with cyclosporine.

## Introduction

Vogt-Koyanagi-Harada (VKH) disease is an autoimmune disorder manifested as bilateral uveitis with or without vitiligo, poliosis, alopecia, meningeal manifestations, and auditory-vestibular dysfunction^1^. It is estimated with an incidence of 0.6 to 1.7 per 100,000 person-years^2^, while this disease is one of the common causes of uveitis and its visual impairment, accounting for 10 to 20% of all uveitis cases^1^. The precise pathogenesis of the disease remains unclear, but it has been theorized as a T-lymphocyte-mediated autoimmunity against melanocytes of all organs triggered in genetically predisposed individuals^1,3,4^. Rapid visual loss following transiently blurred vision is the most common complaint of uveitis in VKH disease^5^. If treated inadequately, this disease typically develops a progressive course from bilateral diffuse choroiditis in the early-phase to granulomatous anterior uveitis in the late phase^5,6^. Patients in the late phase, also known as chronic recurrent VKH disease, may be refractory to treatment, manifesting severe anterior segment inflammation, frequent vision-threatening complications, and a poor visual outcome^7^.

Although several drugs are used for VKH disease in clinical practice, the therapeutic strategy for the disease is generally empirical. Systemic corticosteroids remain the mainstay of treatment for VKH disease, but they frequently cause adverse effects or intolerance which have tempered the overall effectiveness^8^. Some evidence suggests that corticosteroid monotherapy may not be effective enough^7,9,10^. Combination with an immunomodulatory therapy would bring additional benefits in visual improvement and corticosteroid-sparing effects^7,11–14^. Adalimumab, one of anti-tumor necrosis factor (anti-TNF) drugs, is an approved biologic therapy for uveitis, of which the efficacy and safety were shown in phase 3 randomized-controlled trials involving patients with VKH disease^15,16^. Observational studies have also shown that adalimumab was effective to achieve remission in some patients with refractory VKH disease^17,18^. In addition, retrospective studies and early small trials have suggested that conventional immunosuppressants, such as cyclosporine, would be an effective option for VKH disease^5,12,19,20^. Guidance on Noncorticosteroid Systemic Immunomodulatory Therapy in Noninfectious Uveitis have included adalimumab (Grade A recommendation, evidence level 1B) as a biologic therapy and cyclosporine (Grade B recommendation, evidence level 2B) as an immunosuppressive therapy to be considered in the management of patients^21^. However, there is a lack of head-to-head comparisons between immunosuppressant and biologic therapies to guide the medical choices in VKH disease.

In this work, we conducted a pragmatic, blinded-end point, non-inferiority, randomized trial to compare clinical effectiveness and safety profile between cyclosporine-based conventional immunosuppressant strategy and adalimumab-based biologic strategy in combination with corticosteroids for the 26-week induction of remission in VKH disease.

## Results

### Patients

**Fig. 1:**
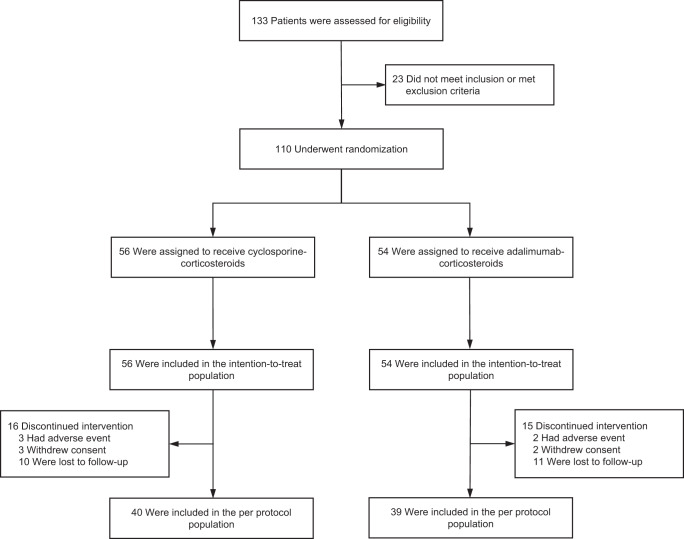
Enrollment, randomization, and analysis populations. A total of 133 patients were screened, and 110 eligible patients underwent randomization and were included in the intention-to-treat population. In this study, 56 patients were assigned to receive cyclosporine plus corticosteroids and 54 patients were assigned to receive adalimumab plus corticosteroids. With the exception of 31 patients who discontinued intervention, a total of 79 patients were included in the per-protocol population.

Between 23 February 2021 and 28 October 2021, a total of 133 patients were screened, and 27 eligible patients with early-phase VKH disease and 83 with late-phase disease underwent stratified randomization (intention-to-treat population), with disease phase as a stratification factor. Of these participants, 56 were assigned to the immunosuppressant group with oral cyclosporine and 54 to the biologic group with adalimumab injection. In this pragmatic trial, 79 patients were included in the per-protocol population (Fig. 1). Overall, the mean (±std) age of the participants was 40.3 ± 12.1 years, and 51 (46.4%) were female. Classification outcomes of disease phase did not differ between the Chinese diagnostic criteria for VKH disease and the Standardization of Uveitis Nomenclature (SUN) criteria for the classification of disease phase (Supplementary Table 1)^6,22^. The median duration of disease was 23 days (interquartile range, 12–40) for patients with early-phase disease and 17 months (interquartile range, 9–43) for those with late-phase disease. All patients had active posterior or panuveitis, and none of them had isolated anterior uveitis only. No patients were receiving systemic corticosteroid therapy at the study entry time point. The two groups of patients had roughly similar demographic and clinical characteristics at baseline (Table 1). During the trial period, oral prednisone was used at a median average daily dose of 20 mg (interquartile range, 19.3–20.1) in the cyclosporine group and at that of 20 mg (interquartile range, 18.8 to 20.2) in the adalimumab group (Supplementary Table 2). Of 56 patients initially assigned to the cyclosporine therapy, the median daily amount of cyclosporine was 150 mg (interquartile range, 150–175). Such a daily amount was given in two divided doses (*b.i.d*.). By week 26, 4 (7.1%) patients in the immunosuppressant group had received step-up treatment with another immunosuppressive agent (Supplementary Table 3).

**Table 1:** Clinical characteristics of participants at baseline

Characteristic	Cyclosporine-corticosteroids (*N* = 56)^a^	Adalimumab-corticosteroids (*N* = 54)^a^
Patient-level characteristic		
Age, year	41.9 ± 12.1	38.7 ± 11.9
Female sex, no. (%)	28 (50.0)	23 (42.6)
BMI, kg/m^2^	24.0 ± 3.6	24.1 ± 4.9
Duration of disease, median (IQR), month	12.0 (1.8–31.5)	12.5 (2.5–34.5)
Disease phase, no. (%)		
Early	14 (25.0)	13 (24.1)
Late	42 (75.0)	41 (75.9)
Systemic symptoms and signs, no. (%)		
Headache	21 (37.5)	16 (29.6)
Neck stiffness	11 (19.6)	13 (24.1)
Vitiligo	6 (10.7)	9 (16.7)
Poliosis	19 (33.9)	18 (33.3)
Alopecia	13 (23.2)	19 (35.2)
Tinnitus	17 (30.4)	17 (31.5)
Hearing loss	15 (26.8)	12 (22.2)
EQ-5D score	0.766 ± 0.182	0.812 ± 0.141
VFQ-25 composite score	51.9 ± 24.5	58.4 ± 22.6
Daily prednisone at baseline, median (IQR), mg	20 (20–25)	20 (20–25)
Eye-level characteristic		
No. of eyes assessed	112	108
BCVA ETDRS score, letter	67.5 ± 24.7	63.8 ± 24.0
Intraocular pressure, median (IQR), mmHg	16 (14–19)	17 (15–19)
Eye with retinal detachment, no./total no. (%)^b^	31/111 (27.9)	18/102 (17.6)
Central macular thickness, median (IQR), μm^c^	238.0 (202.5–281.5)	236.5 (204.8–278.5)
Visual field index, median (IQR), %^d^	88.0 (73.8–95.3)	89.0 (66.0–95.0)
Mean deviation, median (IQR), dB^d^	−7.6 (−13.0 to −4.3)	−8.6 (−16.9 to −4.9)
Pattern standard deviation, median (IQR), dB^d^	3.2 (1.9–5.6)	3.3 (1.9–5.1)

*BMI* body mass index, *IQR* interquartile range, *BCVA* best-corrected visual acuity, *ETDRS* Early Treatment Diabetic Retinopathy Study, *EQ-5D* European Quality of Life-5 Dimensions, *VFQ-25* Visual Functioning Questionnaire-25.

^a^Plus-minus values are means ± SD.

^b^Retinal detachment was assessed with optical coherence tomography (OCT). Two eyes from one patient did not undergo the OCT examination. Five eyes from three patients received the OCT examination, but the status of retinal detachment could not be accurately assessed.

^c^Central macular thickness was measured by OCT. Two eyes from one patient did not undergo the OCT examination. In addition, values of 25 eyes were not measured out by OCT.

^d^Values of 7 eyes from five patients were not measured out by the examination of visual field due to low vision. The visual field index represents an overall parameter, ranging from 0% indicating perimetrically blindness to 100% indicating perimetrically normal field. The mean deviation represents the total amount of visual field loss, with normal values ranging from 0 to −2 dB. The pattern standard deviation represents the irregularity by adding the absolute values of differences between the threshold and the mean visual field sensitivity at each point.

### Primary outcome

**Fig. 2:**
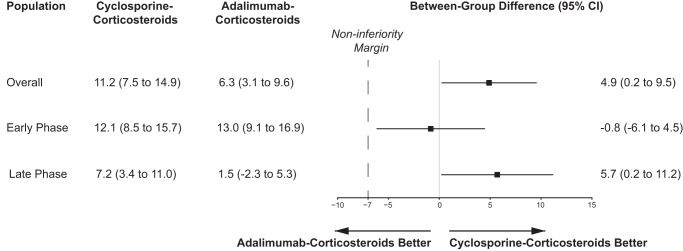
Primary outcome. The primary outcome was the change from baseline in best-corrected visual acuity measured as Early Treatment Diabetic Retinopathy Study (ETDRS) score at week 26. The least-squares mean changes from baseline and between-group differences were adjusted for baseline value, disease phase, and the correlation between the eyes of the same patient, with the use of the generalized estimating equation. *n* = 220 independent eyes for overall patients; *n* = 54 independent eyes for early-phase patients; *n* = 166 independent eyes for late-phase patients. Source data are provided as a Source Data file.

In the intention-to-treat population, the mean change in best-corrected visual acuity (BCVA) measured as the Early Treatment Diabetic Retinopathy Study (ETDRS) score from baseline to week 26 was 11.2 letters (95% confidence interval [CI], 7.5–14.9) in the immunosuppressant group, as compared with 6.3 letters (95% CI, 3.1–9.6) in the biologic group (difference, 4.9; 95% CI, 0.2–9.5; one-sided *P* value < 0.001 for non-inferiority). The lower limit of the confidence interval of the difference was greater than the prespecified non-inferiority margin of −7, suggesting that the criteria for non-inferiority for cyclosporine-based immunosuppressant strategy could be met. Subgroup analysis showed no effect modification according to the disease phase (two-sided *P* value = 0.309 for interaction). The between-group difference was −0.8 letters (95% CI, −6.1–4.5) in early-phase VKH disease and 5.7 letters (95% CI, 0.2–11.2) in late-phase VKH disease (Fig. 2). The lower limit of the confidence interval of the difference was even greater than 0 in the stratum of late-phase VKH disease, but that was merely greater than −7 in early-phase disease.

In the overall per protocol population, a more discernible difference in point estimate was observed, but the analysis was relatively less powered, leading to a wider confidence interval (difference, 6.2; 95% CI, 0.1–12.4; Supplementary Table 4). The between-group difference was −1.0 letters (95% CI, −5.1–3.2) in early-phase VKH disease and 6.6 letters (95% CI, −0.9–14.2) in late-phase VKH disease in the per protocol analysis.

A total of 10 sensitivity analyses that addressed assumptions on potential confounding, data missing, conservative estimate, and working correlation structures, respectively, produced largely consistent results with the primary finding, suggesting the non-inferiority for the immunosuppressant group (Supplementary Table 5–14). However, the lower limits of the confidence interval of the between-group difference in sensitivity analyses were not always greater than 0.

### Secondary outcomes

Results of secondary outcomes at 26 weeks are provided in Table 2. Improvement in ETDRS score by 15 letters or greater in both eyes was reached in 19.1% of patients in the immunosuppressant group and in 11.0% of those in the biologic group (difference, 8.0%; 95% CI, −8.1%–24.2%). Inactive uveitis in both eyes was achieved in 92.3% of patients in the immunosuppressant group, as compared with 81.7% of those in the biologic group (difference, 10.6%; 95% CI, −3.2%–24.4%). Both groups showed an improvement in visual field indices, a decrease in central macular thickness, and an increase in European Quality of Life-5 Dimensions (EQ-5D) score and Visual Functioning Questionnaire-25 (VFQ-25) composite score. No differences were observed between these two groups.

**Table 2:** Secondary outcomes at week 26 in intention-to-treat population

Outcome	Cyclosporine-Corticosteroids (*N* = 56)	Adalimumab-Corticosteroids (*N* = 54)	Difference (95% CI)^a^
Improvement in ETDRS score by 15 letters or greater			
No. of patients assessed	43	41	-
Percentage of patients who achieved in either eye (95% CI)^b^	37.0 (24.6–55.8)	24.6 (14.0–43.3)	12.4 (−8.1–33.0)
Percentage of patients who achieved in both eyes (95% CI)^b^	19.1 (9.9–36.8)	11.0 (4.4–27.6)	8.0 (−8.1–24.2)
Inactive uveitis in both eyes			
No. of patients assessed	43	41	-
Percentage of patients who achieved (95% CI)^b^	92.3 (77.8–97.3)	81.7 (60.7–91.5)	10.6 (−3.2–24.4)
Visual field index			
No. of eyes assessed	80	78	-
Change from baseline (95% CI), %^c^	10.0 (7.3–12.7)	10.3 (7.6–13.1)	−0.4 (−4.3–3.6)
Mean deviation			
No. of eyes assessed	80	78	-
Change from baseline (95% CI), dB^c^	4.1 (3.1–5.1)	4.0 (3.0–5.1)	0.1 (−1.3–1.5)
Pattern standard deviation			
No. of eyes assessed	80	78	-
Change from baseline (95% CI), dB^c^	−0.9 (−1.4 to −0.3)	−0.3 (−0.7–0.2)	−0.6 (−1.3–0.1)
Resolution of retinal detachment			
No. of eyes assessed^d^	26	13	-
Percentage of eyes with resolution (95% CI)	92.3 (75.9–97.9)	100 (77.2–100)	−7.7 (−24.1–15.8)
Central macular thickness			
No. of eyes assessed	78	64	
Change from baseline (95% CI), μm^c^	−82.7 (−94.0 to −71.4)	−70.0 (−80.9 to −59.1)	−12.7 (−30.4–5.0)
EQ-5D score			
No. of patients assessed	43	41	-
Change from baseline (95% CI)^e^	0.068 (0.027–0.110)	0.082 (0.038–0.126)	−0.013 (−0.079–0.052)
VFQ-25 composite score			
No. of patients assessed	43	41	-
Change from baseline (95% CI)^e^	20.1 (15.3–24.9)	20.1 (14.8–25.4)	0.0 (−6.7–6.6)
Step-treatment before week 26			
No. of patients assessed^f^	56	-	-
No. of patients who received (%)	4 (7.1)	-	-

*BCVA* best-corrected visual acuity, *ETDRS* Early Treatment Diabetic Retinopathy Study, *EQ-5D* European Quality of Life-5 Dimensions, *VFQ-25* Visual Functioning Questionnaire-25.

^a^The 95% confidence intervals were not adjusted for multiple comparisons.

^b^The percentages of patients and the differences between the two groups were adjusted for disease phase, with the use of the log-binomial regression.

^c^The least-squares mean changes and between-group differences were adjusted for baseline value, disease phase, and the correlation between eyes of the same patient, with the use of the generalized estimating equation.

^d^This end point was assessed in eyes with retinal detachment at baseline and with available data at 26 weeks.

^e^The least-squares mean changes and between-group differences were adjusted for baseline value and disease phase, with the use of the generalized estimating equation.

^f^This end point was assessed in patients initially assigned to the cyclosporine-corticosteroids group.

### Adverse events

Adverse events occurred with a total incidence of 15.74 events per patient-year in participants receiving cyclosporine plus corticosteroids and with that 14.48 events per patient-year in those receiving adalimumab plus corticosteroids. Serious adverse events were reported more frequently in participants receiving adalimumab plus corticosteroids than in those receiving cyclosporine plus corticosteroids (1.21 vs. 0.70 events per patient-year) (Table 3), due to a higher incidence of hospitalization for ocular hypertension or glaucoma (considered by the investigators to be possibly associated with VKH disease). The total incidence of adverse events possibly associated with the disease was also higher with adalimumab plus corticosteroids (Table 3). All adverse events are reported in Supplementary Table 15. No new safety signals were identified in both groups.

**Table 3:** Adverse events in safety population

Event	Cyclosporine–corticosteroids (*N* = 56)		Adalimumab–corticosteroids (*N* = 54)	
	No. of events	Events/100 person-years	No. of events	Events/100 person-years
Any adverse event	384	1573.8	372	1447.5
Serious adverse event	17	69.7	31	120.6
Hospitalization for cataract surgery	14	57.4	12	46.7
Hospitalization for ocular hypertension	2	8.2	14	54.5
Glaucoma	0	0.0	3	11.7
Pneumonitis	1	4.1	0	0.0
Shingles	0	0.0	1	3.9
Corneal ulcer	0	0.0	1	3.9
Adverse event leading to discontinuation of trial drugs	3	12.3	2	7.8
Adverse event possibly associated with trial drugs	314	1286.9	257	1000.0
Adverse event possibly associated with VKH disease	70	286.9	115	447.5
Adverse event that occurred in ≥10% of total participants				
Alanine aminotransferse increased	9	36.9	8	31.1
Arthragia	13	53.3	0	0.0
Blood bilirubin increased	17	69.7	9	35.0
Blood lactate dehydrogenase increased	9	36.9	3	11.7
Blood urea nitrogen increased	13	53.3	9	35.0
Cataract	15	61.5	14	54.5
Creatinine increased	13	53.3	7	27.2
Erythrocytes sedimentation rate increased	10	41.0	9	35.0
Glomerular filteration rate decreased	11	45.1	6	23.3
Hemoglobin increased	8	32.8	7	27.2
Hyperglycemia	15	61.5	12	46.7
Hypertension^a^	20	82.0	17	66.1
Hyperuricemia	19	77.9	16	62.3
Lymphocyte count increased	25	102.5	37	144.0
Leukocytosis	37	151.6	38	147.9
Iris synechiae	4	16.4	16	62.3
Ocular hypertension	31	127.0	61	237.4

^a^Hypertension was defined as a pathological increase in blood pressure to the extent of Grade 1 or higher (systolic blood pressure ≥120 mmHg or diastolic blood pressure ≥80 mmHg) according to Common Terminology Criteria for Adverse Events (CTCAE), V5.0.

## Discussion

In this 26-week, comparative effectiveness trial involving patients with VKH disease, 75% of whom had a late-phase disease, we found that the immunosuppressant strategy with cyclosporine plus corticosteroids was non-inferior to the biologic strategy with adalimumab plus corticosteroids with respect to visual improvement. Of note, the systemic corticosteroid regimen used in this trial was different from that in standard care. The overall incidence of adverse events was similar with two types of treatment, while, there were fewer serious adverse events reported in the immunosuppressant group than in the biologic group in the trial.

Our trial used a modified, non-standard, corticosteroid background therapy for the treatment of VKH disease. Specifically, for those with early-phase VKH disease, the initial prednisone dose was 30–40 mg per day (0.6–0.8 mg per kilogram per day). The daily dose of prednisone we commenced was lower than that in previous studies (1 mg per kilogram of body weight or higher)^14,23,24^. The purpose of treatment with a lower dose of corticosteroids was to minimize the incidence of adverse events, especially when another immunomodulatory drug was added. In this trial, such a regimen of corticosteroids combined with cyclosporine or adalimumab resulted in infrequent systemic serious adverse events. Our effectiveness data also suggest that either combination regimen could, to some extent, improve visual acuity and mitigate uveitis activity. Notwithstanding, a standard treatment for early-phase VKH disease is currently the high-dose corticosteroid monotherapy without the need for immunomodulatory therapies^25^. Our trial, along with a previous study^12^, provides some effectiveness and safety data for the combination regimen of reduced corticosteroids with immunomodulatory agents. However, the comparative effectiveness with the standard care for early-phase VKH disease requires further investigation.

For patients with late-phase VKH disease, our initial prednisone dose was 20–30 mg per day (0.4–0.6 mg per kilogram per day). Such a corticosteroid regimen was chosen based on several considerations. First, the late-phase VKH disease is characterized by chronic recurrent intraocular inflammation requiring prolonged treatment^26^. High-dose prednisone may be useful in early-phase VKH disease^27^, but it cannot be used for a long period because of its significant side effects. Second, when the disease progresses to the late phase with chronic recurrent evolution, patients have missed the therapeutic window of opportunity^9^, and aggressive corticosteroids are no longer able to modify the phenotype and prognosis of the disease^28^. In addition, a number of studies suggested the need for immunomodulatory therapies added to corticosteroids for late-phase disease^7,10,28,29^. Prior evidence suggested that our corticosteroid regimen showed a low risk of intolerance and treatment interruption, especially when combined with additional immunomodulatory therapies^12^.

Our tapering strategy is also different from that used in standard care. Considering that corticosteroid therapy remains the mainstay of treatment of VKH disease and usually lasts over a year in real-world practice^25,30,31^, we had not tapered prednisone to a successfully corticosteroid-sparing dose (7.5 mg daily or less) by week 26^32,33^. Instead, we tapered prednisone until the lowest dose of 15 mg daily was maintained. On average, our patients were treated with prednisone at a median average daily dose of 20 mg to maintain an adequate therapeutic effect, which would be useful to examine whether there were additional benefits of other immunomodulatory drugs. However, this practice has complicated the attempts to compare the effectiveness between cyclosporine and adalimumab. Because the patients, especially those with early-phase disease, are still likely to be controlled by the prednisone, such an adequate level of corticosteroid therapy may have influenced the non-inferiority outcome. The non-inferiority results may be due to the inclusion of those participants for whom addition of immunosuppression to sufficient corticosteroid therapy is unnecessary. Nevertheless, our data suggest that there is likely to be additional benefit from cyclosporine in late-phase disease, which has not been masked by corticosteroids. We found that the lower limit of confidence interval of the between-group difference was greater than 0 in late-phase disease. But, this finding derived from a subgroup and should be considered exploratory only rather than definitive. In general, the superiority has not been supported by our data and requires formal testing in future trials.

In this study, patients with late-phase VKH disease accounted for 75% of those enrolled. The specific prevalence of late-phase disease in this cohort may differ from that in other centers, in which late-phase VKH disease could be rarely seen due to early diagnosis and treatment. There seem to be unmet medical needs in patient with VKH disease in China, which have enabled us to recruit a number of patients with late-phase VKH disease during a relatively short period. Nevertheless, interpretation of findings may be limited by the fact that the majority of study participants were late-phase VKH disease. Generally, our study extended previous studies favoring the immunosuppression as a safe and effective option for late-phase VKH disease^5,12,29^. In the immunosuppressant group, we used a dosing schedule of cyclosporine that was in line with previous studies^12,14,19,34,35^. We also anticipated the possibility of initiation of step-up treatment, but it was actually used in less than 10% of patients. Conservative estimate with the exclusion of effects of step-up treatment in sensitivity analysis consistently showed the non-inferiority for cyclosporine plus corticosteroids. Therefore, the first-line treatment with cyclosporine plus corticosteroids would be useful for the majority of patients with late-phase VKH disease, of which the cost-effectiveness warrants further studies.

Overall, adalimumab plus corticosteroids resulted in less visual improvement in the point estimate than cyclosporine plus corticosteroids. This outcome was mainly due to the relatively modest visual improvement by adalimumab in the late-phase VKH disease. Some previous studies similarly showed that adalimumab effectively controlled intraocular inflammation in late-phase VKH disease but that visual improvement was not statistically significant^18,36^. The possible reasons may be, at least partially, related to the suboptimal effect of adalimumab in preventing or improving ocular complications as observed in our trial. We found that disease-related adverse events or complications (e.g. ocular hypertension, secondary glaucoma, iris synechiae), which are usually seen in late-phase disease^37^, occurred more frequently in the adalimumab group than in the cyclosporine group. These complication outcomes were in line with the observed visual prognosis. The primary outcome, BCVA, is a functional outcome of key relevance to VKH disease, which may objectively and sensitively reflect the overall visual function and the severity of intraocular inflammation and ocular complications. Nevertheless, the effect of adalimumab on ocular complications in late-phase disease requires further validation.

Strengths of the trial include the involvement of refractory patients with poorer vision (ETDRS score of 20 letters or less) as compared with former trials to maximize real-world applicability and generalizability^15,16^. The non-inferiority margin for the primary outcome has historical precedent^32^. Blinded measurement of BCVA would limit the potential for bias in estimated treatment effects. Consistent findings from objective (e.g., BCVA) and subjective (e.g., clinical assessment of disease activity, quality of life) measures further mitigate concerns about bias.

Several important limitations of our trial should be noted. First, as mentioned earlier, our tapering regimen of prednisone did not correspond to the standard tapering regimen for corticosteroid sparing^32,33^. Our practice was originally designed to explore the additional benefits of immunomodulatory drugs under the adequate treatment of background corticosteroids. But, we recognize that a daily dose of 15 mg or more of prednisone for 26 weeks has not been used in many other centers and may be hardly generalizable, and this practice may have confounded the ability to independently interpret comparative effectiveness of cyclosporine and adalimumab. Second, this trial intends for the induction of remission to rapidly control intraocular inflammation and improve BCVA by week 26, but patients with late-phase VKH disease usually require subsequent maintenance therapy over a long period. We have not yet assessed long-term outcomes. Third, VKH disease mostly affects Asian people, whereas is infrequent in European and African descent^38^. Our results may need further validation in patients other than Asians. Fourth, we recognize that cyclosporine is not preferentially used as the first-line medication among immunosuppressive drugs for uveitis in some regions, although it has been used in our clinical practice and has shown effectiveness in late-phase VKH disease^1,5,12^. It is not clear whether its effectiveness and treatment response would be varied with different ethnic groups or local practice. Fifth, because VKH disease is individually rare, recruitment of patients was conducted in a specialized uveitis center due to patient clustering and clinical convenience, but the residences of participants were actually scattered across mainland China. The ongoing COVID-19 pandemic and travel restriction in China prevented some remote-living patients from completing the week 26 visit.

In conclusion, combined with a non-standard corticosteroid regimen, cyclosporine-based immunosuppressant strategy was found to be non-inferior to adalimumab-based biologic strategy by 26 weeks for visual improvement in a cohort of patients with Vogt-Koyanagi-Harada disease, 75% of whom had a late-phase disease.

## Methods

### Trial design and participants

The Ethics Committee of the First Affiliated Hospital of Chongqing Medical University approved the trial. All trial procedures adhered to the provisions of the Declaration of Helsinki and Good Clinical Practice guidelines. This trial was conducted at Uveitis Center of the First Affiliated Hospital of Chongqing Medical University, Chongqing, China. This academic, university-affiliated, teaching hospital has set up the large, specialized medical center for uveitis care, having provided service to a total of 2079 VKH disease from 32 provincial administrative regions across mainland China as of 2018^39^. No commercial sponsors were involved in the trial. This trial was registered with Chinese Clinical Trial Registry, ChiCTR2100043061. The administrative department of scientific research of the First Affiliated Hospital of Chongqing Medical University oversaw the conduct, progress, integrity, and safety outcomes of the trial. Written informed consent was obtained from all patients. There is no participant compensation. Sex and/or gender were not considered in the study design. Sex and/or gender of participants were determined based on self-reporting to the treating physician. Difference between sex and gender was not relevant to the topic of the study. No sex- and gender-based analyses have been performed because sex and/or gender were not considered a confounder in this randomized study.

Adult patients (≥18 years) who had active VKH disease within the last 90 days and/or had a justification for remission induction with chronic oral prednisone at 20 mg per day or greater were eligible to participate^6^. Patients were considered an active disease if they had any of the following manifestations in either eye: anterior chamber cell grade ≥2+; vitreous haze grade ≥2+; or active inflammatory choroidal or retinal lesions detected by optical coherence tomography (OCT) or fluorescein fundus angiography (FFA). Anterior chamber cells were evaluated at grades from 0 to 4+ according to the Standardization of Uveitis Nomenclature (SUN) criteria, with higher values indicating poorer condition^40^. Vitreous haze was assessed at levels from 0 to 4+ according to the National Eye Institute (NEI) criteria modified by the SUN working group, with higher grades indicating worse condition^40,41^. Key exclusion criteria included visual acuity of hand motions or worse in the better-seeing eye and previous exposure to anti-TNF therapy. Supplementary Table 16 provides the full eligibility criteria.

### Randomization and treatment

Participants were randomly assigned to the biologic (adalimumab) group or the immunosuppressant (cyclosporine) group in a 1:1 ratio, stratified by disease phase (early vs. late) according to the Chinese diagnostic criteria^6^. Our trial was initiated earlier before a new set of classification criteria for early and late-stage VKH disease was reported by the SUN working group in August, 2021^22^. The SUN criteria had similar aspects to the Chinese diagnostic criteria. We performed a post-hoc evaluation of the consistency in classification outcomes between these two sets of criteria in a blinded manner. Random assignment sequence was computer generated, and allocations were obtained by telephone from a designated coordinator who was not involved in other parts of the trial. Participants and treating clinicians were not masked to treatment assignment.

The period of remission induction was 26 weeks. Patients in the biologic group received subcutaneous injection with adalimumab at 40 mg every 2 weeks. We followed the practice in some real-world settings^42–44^, where adalumimab was not given with a loading dose for chronic recurrent VKH disease. Patients in the immunosuppressant group received oral cyclosporine at a total amount of 100–200 mg per day (approximately equivalent to 2–4 mg per kilogram of body weight per day). Based on the pragmatic design of the trial, the specific dosage could be individualized within the range and prescribed at the treating clinician’s discretion. In participants initially assigned to cyclosporine therapy, step-up treatment was recommended if any worsening signs relative to the baseline condition developed, such as two-step increase in anterior chamber cell grade or vitreous haze grade, or occurrence of new active, inflammatory chorioretinal lesions, however, these criteria were not required to be met. Step-up treatment could be addition of oral chlorambucil (0.05–0.1 mg per kilogram per day)^5^ or transition to adalimumab injection (40 mg every 2 weeks), but the decision was ultimately made by the treating clinician.

Both groups additionally received the same corticosteroid regimen. Previous studies have indicated more intensive treatment for early-phase VKH disease^9^. In this stratified randomized trial, initial doses of prednisone were 30–40 mg per day (0.6–0.8 mg per kilogram per day) in early-phase VKH disease, and 20–30 mg per day (0.4–0.6 mg per kilogram per day) in late-phase disease. Such doses were used for 1–2 weeks and then decreased by 5 mg every 1–2 weeks, with a goal of tapering and holding at 15 mg per day over the remission induction period.

Topical corticosteroid drops combined with cycloplegic and mydriatic agents were used and adjusted according to the condition of anterior segment inflammation. Topical corticosteroids were tapered to discontinuation over four weeks after the anterior segment inflammation subsided. Prohibited therapies included glucocorticosteroid implant, periocular and intravitreal injections.

### Outcomes and measures

Trial visits were scheduled at week 2, week 9, week 17, and week 26. Clinical evaluation of uveitis activity was performed by the unmasked treating clinician with the use of slit-lamp microscopy and ophthalmoscopy with pupillary dilation. Inactive uveitis was defined as eyes with an anterior chamber cell grade ≤0.5+, a vitreous haze grade ≤0.5+, and no active inflammatory choroidal or retinal lesions. Examinations of best-corrected visual acuity (BCVA), intraocular pressure, visual field (central 24-degree tested with a 54-point grid), fundus photography, OCT, and FFA were carried out by masked certified personnel who were unaware of the treatment assignment with a standardized procedure (Supplementary Table 17). Evaluation of images of fundus photography, OCT, and FFA were performed by trained readers at a central reading center who were blinded to the treatment group. BCVA was measured with the letter score (range: 0 to 100, greater values with better visual acuity) of the Early Treatment Diabetic Retinopathy Study (ETDRS) acuity chart read by the patient at a starting distance of 4 meters, with a repetition at 1 meter if necessary. Participants completed European Quality of Life-5 Dimensions (EQ-5D; range 0 to 1, greater values with better general health status)^45^ and Visual Functioning Questionnaire-25 (VFQ-25; range: 0 to 100, greater values with better vision-related quality of life)^46^ at baseline and week 26. The primary outcome was the change from baseline in BCVA ETDRS score at week 26. According to prior clinical trial data^32^, the minimal clinically important difference (MCID) for changes in ETDRS score is −7 letters.

Secondary outcomes included the proportion of patients achieving an improvement in ETDRS score by 15 letters or greater at week 26, the proportion of patients achieving inactive uveitis in both eyes at week 26, the changes from baseline to week 26 in visual field indices, including visual field index, mean deviation, and pattern standard deviation, the resolution of retinal detachment detected by OCT at week 26, the change from baseline in central macular thickness on OCT at week 26, the proportion of patients initially assigned to cyclosporine therapy who received step-up treatment, and the changes from baseline in EQ-5D score and VFQ-25 composite score at week 26. Safety outcomes included frequency, severity, and relatedness of adverse events through week 26, adjudicated by independent safety monitors.

### Statistics and reproducibility

Statistical method was used to predetermine sample size. We hypothesized that cyclosporine plus corticosteroids would result in a mean change in ETDRS score that was no less than seven letters (MCID) lower than the mean change by adalimumab plus corticosteroids. Assuming the true difference of 0 between the two treatment groups in mean changes and a standard deviation of 10, we estimated that a sample size of 110 participants, accounting for a dropout rate of 20%, would provide 90% power to show the non-inferiority at a margin of −7 letters at a one-side significance level of 2.5%.

This was a randomized study. No data were excluded from the primary analysis. The Investigators were aware of treatment allocation during trial, but they were blinded to primary outcome assessment.

The primary effectiveness analyses were based on the intention-to-treat population comprised all randomized patients regardless of their adherence to assigned treatment as well as on the per-protocol population that included the subset of patients who completed a full treatment course without major protocol violations. Safety analyses were based on the safety population that included all those having received at least one dose of assigned treatment.

The primary outcome as well as the other continuous outcomes was analyzed with the generalized estimating equation with identity link to calculate the least-squares mean of changes from baseline to week 26 in each treatment group and the difference in means between the two treatment groups, accounting for baseline value, disease phase and the correlation between eyes of the same individual (if the unit of analysis was by eye instead of by individual). We used the independence assumption about the correlation structure with the robust standard error estimator in the generalized estimating equation. We also tested other assumptions about the correlation structure in sensitivity analyses but found that the choice of working correlation matrix had little impact on results (Supplementary Table 14). Binary secondary endpoints were analyzed by the treatment group with the log-binomial regression accounting for the disease phase. Missing data on the primary outcome were imputed with the use of the last observation carried forward. There was no plan to impute missing values for secondary outcomes. For the primary outcome, non-inferiority was assessed with a Wald test by comparing the observed between-group difference with the non-inferiority margin. Unless otherwise specified, all reported P values were one-sided. A one-sided *P* value < 0.025 was considered statistically significant. A prespecified subgroup analysis compared the effects between early and late-phase VKH disease by including the treatment-by-subgroup interaction term in the regression equation. Several prespecified and post hoc sensitivity analyses are summarized in Supplementary Table 5. The results of secondary outcomes and sensitivity analyses should not be used for drawing formal clinical inferences. Data were analyzed with the use of IBM SPSS Statistics, version 25.0.

### Reporting summary

Further information on research design is available in the Nature Portfolio Reporting Summary linked to this article.

## Supplementary information

Supplementary Information

Reporting Summary

## Data Availability

The complete de-identified patient data set are available under restricted access because they contain information on health records and making the data publicly available without additional consent or ethical approval might compromise patient’s privacy and the original ethical approval, and data access can be obtained by request from the corresponding author (peizengycmu@126.com). The timeframe for response to requests will be within two weeks. Data will be shared for non-commercial purposes, with investigator support, after approval of a proposal, and with a signed data access agreement. The trial protocol and statistical analysis plan are provided with the paper. Source data are provided with this paper.

## Source data

Source Data
