# Supplementary material for: A randomized non-inferiority trial of therapeutic strategy with immunosuppressants versus biologics for Vogt-Koyanagi-Harada disease
Source: Nat Commun. 2023 Jun 24;14:3768. doi: 10.1038/s41467-023-39483-5 (PMC10290648; doi:10.1038/s41467-023-39483-5)
Supplement: Supplementary file 1 — Supplementary Information [file 41467_2023_39483_MOESM1_ESM.pdf]

A randomized non-inferiority trial of therapeutic strategy with immunosuppressants versus biologics for Vogt-Koyanagi-Harada disease

Supplementary Information

Supplementary Table 1. Classification Outcome on Disease Phase 2

Supplementary Table 2. Prednisone Use throughout Trial Period 3

Supplementary Table 3. Step-up Treatment among Patients Initially Assigned to Cyclosporine-Corticosteroids Treatment 4

Supplementary Table 4. Primary Outcome in Per Protocol Population 5

Supplementary Table 5. Summary of Sensitivity Analyses on Primary Outcome 6

Supplementary Table 6. Sensitivity Analysis of Adjustment for Prednisone Dose 8

Supplementary Table 7. Sensitivity Analysis of Adjustment for Ocular Surgery 9

Supplementary Table 8. Sensitivity Analysis of Adjustment for Full Covariates 10

Supplementary Table 9. Sensitivity Analysis of Modified Intention-To-Treat Analysis 11

Supplementary Table 10. Sensitivity Analysis of Limited to Higher ETDRS Score 12

Supplementary Table 11. Sensitivity Analysis of Multiple Imputation 13

Supplementary Table 12. Sensitivity Analysis of Excluding Effects of Step-up Treatment 15

Supplementary Table 13. Sensitivity Analysis of Inverse Probability Weighting for Non-Missing Rate 16

Supplementary Table 14. Sensitivity Analysis of Working Correlation Structures in Generalized Estimating Equation 17

Supplementary Table 15. Full List of Adverse Events 18

Supplementary Table 16. Eligibility Criteria 22

Supplementary Table 17. Device of Ophthalmic Auxiliary Examination 25

Supplementary References 26

Supplementary Table 1. Classification Outcome on Disease Phase

SUN Criteria [Ref. 1]	Chinese Criteria [Ref. 2]		Total No. of Participants
	Early-Phase VKH Disease	Late-Phase VKH Disease	
**Early-Stage VKH Disease**	27	0	27
**Late-Stage VKH Disease**	0	83	83
**Total No. of Participants**	27	83	110

Supplementary Table 2. Prednisone Use throughout Trial Period

Variable	Cyclosporine-Corticosteroids	Adalimumab-Corticosteroids
**Overall**	**N = 56**	**N = 54**
Cumulative dose*		
Median	3350	3395
Interquartile range	2220 to 3611	2233 to 3636
Average daily dose†		
Median	20	20
Interquartile range	19.34 to 20.07	18.83 to 20.17
**Early phase**	**N = 14**	**N = 13**
Cumulative dose*		
Median	3542	3560
Interquartile range	2066 to 3792	3255 to 3770
Average daily dose†		
Median	20.14	20
Interquartile range	19.59 to 21.15	19.68 to 20.51
**Late phase**	**N = 42**	**N = 41**
Cumulative dose*		
Median	3338	3300
Interquartile range	2203 to 3556	2045 to 3623
Average daily dose†		
Median	20	20
Interquartile range	19.18 to 20	18.5 to 20.04

* The cumulative dose was defined as the total dose of prednisone or equivalent dose accumulated per day over the trial period.

† The daily dose was calculated by dividing the cumulative dose by the treatment duration (days).

Supplementary Table 3. Step-up Treatment among Patients Initially Assigned to Cyclosporine-Corticosteroids Treatment

Variable	Value
Total no. of patients	56
No. of patients treated	4
Adding agent, no./total no. (%)	
Chlorambucil	3/4 (75.0)
Mycophenolate mofetil	1/4 (25.0)
Time point of initiation	
Week 9	3/4 (75.0)
Week 17	1/4 (25.0)

Supplementary Table 4. Primary Outcome in Per Protocol Population

Primary Outcome*	Cyclosporine-Corticosteroids (95% CI) N = 40	Adalimumab-Corticosteroids (95% CI) N = 39	Difference (95% CI)
Overall	13.7 (9.0 to 18.3)	7.4 (3.3 to 11.6)	6.2 (0.1 to 12.4)
Early phase	14.2 (11.6 to 16.8)	15.2 (12.1 to 18.3)	-1.0 (-5.1 to 3.2)
Late phase	8.4 (3.5 to 13.3)	1.8 (-3.7 to 7.3)	6.6 (-0.9 to 14.2)

* The least-squares mean changes and between-group differences were adjusted for baseline value, disease phase and the correlation between eyes of the same patient, with the use of the generalized estimating equation.

Supplementary Table 5. Summary of Sensitivity Analyses on Primary Outcome

Sensitivity Analysis	Summary
Adjustment for Prednisone Dose*	Cumulative prednisone dose throughout 26 weeks was further added to the primary analysis generalized estimating equation (GEE) as a covariate. The analysis was conducted in the intention-to-treat (ITT) population.
Adjustment for Ocular Surgery*	Ocular surgery variables (receiving one specific surgery or not during the trial period) were further added to the primary analysis GEE as covariates. The analysis was conducted in the ITT population.
Adjustment for Full Covariates*	Cumulative prednisone dose, ocular surgery variables, age, sex, body mass index, central macular thickness and visual field indices (due to the potential multicollinearity, only visual field index used as a representative) measured at baseline were further added to the primary analysis GEE as covariates. The analysis was conducted in the ITT population.
Modified Intention-To-Treat Analysis*	The primary outcome was assessed in the modified intention-to-treat (mITT) population which included all participants who underwent randomization, with the exception of those who did not start any assigned treatment and those who did not have any primary outcome data at week 26. No data imputation was performed for mITT analysis. The mITT analysis eventually excluded 26 patients who withdrew or were lost to follow-up before week 26 from the ITT population. This analysis would test whether results were sensitive to data missing.
Limited to Higher ETDRS Score*	ETDRS score measurement may not be sensitive to a low vision. Therefore, the primary outcome was assessed in the ITT population only involving eyes with an ETDRS score of 20 letters or greater measured at baseline in the sensitivity analysis.
Prespecified Multiple Imputation*	Missing data were considered to be missing at random because the reason that data missing was not related to the treatment group and the numbers of patients with missing primary outcome data were similar between the two treatment groups. Missing data on the primary outcome were predicted with multiple imputation technique based on baseline information about ETDRS score, age, sex, body mass index, disease phase, central macular thickness and visual field indices (due to the potential multicollinearity, only visual field index used as a representative) with or without the treatment group as a predictor. The primary outcome was assessed in 50 imputed datasets and then combined with the use of the

	Rubin's rules.
Post-Hoc Multiple Imputation†	A post hoc multiple imputation was performed based on the same assumption of above mentioned prespecified ones. The post-hoc one predicted missing data on the primary outcome with the use of variables including treatment group, baseline information about age, sex, body mass index, disease phase, best-corrected visual acuity ETDRS score, central macular thickness and visual field index, and available data of each follow-up visit on best-corrected visual acuity ETDRS score and central macular thickness. The primary outcome was assessed in 50 imputed datasets and then combined with the use of the Rubin's rules.
Excluding Effects of Step-up Treatment*	The primary outcome data were assigned as the visual acuity value at the clinic visit when the step-up treatment was initiated. This analysis would produce more conservative estimate of effectiveness of cyclosporine therapy.
Inverse probability weighting for non-missing rate†	To account for the potential bias incurred by missing outcome data of individuals who withdrew or were lost to follow-up before week 26, we conducted an inverse-probability-weighted analysis. We used the logistic regression to predict the probability of being a complete case (non-missing rate) based on the following baseline factors: age, sex, body mass index, month of enrollment, province of residence, education level, marital status and occupation. This prediction model yield an area under the receiver operating characteristic (ROC) curve of 0.904 (95% CI, 0.847 to 0.961), Hosmer-Lemeshow test of P value =0.699, and a pseudo R^2 of 0.569. The weight of each participant was given by the inverse of the predicted probability of being a complete case. [Ref. 3] Then, the primary analysis was re-performed only on the non-missing observations (modified intention-to-treat population) with the weighted model.
Working Correlation Structures†	The primary analysis used the Independence Working Correlation Matrix with robust standard error estimator (also called the Huber/White/sandwich estimator). To assess the robustness of data with different working correlation structures in the generalized estimating equation, we reanalyzed the primary outcome data with the use of Exchangeable Correlation Matrix and Unstructured Correlation Matrix, respectively.

* These analyses were prespecified.

† These analyses were performed post-hoc.

Supplementary Table 6. Sensitivity Analysis of Adjustment for Prednisone Dose

Primary Outcome	Cyclosporine-Corticosteroids (95% CI) N = 56	Adalimumab-Corticosteroids (95% CI) N = 54	Difference (95% CI)
**Primary analysis***			
Overall	11.2 (7.5 to 14.9)	6.3 (3.1 to 9.6)	4.9 (0.2 to 9.5)
Early phase	12.1 (8.5 to 15.7)	13.0 (9.1 to 16.9)	-0.8 (-6.1 to 4.5)
Late phase	7.2 (3.4 to 11.0)	1.5 (-2.3 to 5.3)	5.7 (0.2 to 11.2)
**Sensitivity analysis†**			
Overall	11.2 (7.5 to 14.8)	6.2 (3.1 to 9.4)	5.0 (0.4 to 9.5)
Early phase	12.6 (10.3 to 14.8)	12.5 (10.0 to 15.1)	0.0 (-3.4 to 3.5)
Late phase	7.2 (3.4 to 10.9)	1.5 (-2.4 to 5.3)	5.7 (0.2 to 11.1)

* The least-squares mean changes and between-group differences were adjusted for baseline value, disease phase and the correlation between eyes of the same patient, with the use of the generalized estimating equation.

† The least-squares mean changes and between-group differences were adjusted for baseline value, disease phase, the correlation between eyes of the same patient, and cumulative prednisone dose throughout 26 weeks, with the use of the generalized estimating equation.

Supplementary Table 7. Sensitivity Analysis of Adjustment for Ocular Surgery

Primary Outcome	Cyclosporine-Corticosteroids (95% CI) N = 56	Adalimumab-Corticosteroids (95% CI) N = 54	Difference (95% CI)
**Primary analysis***			
Overall	11.2 (7.5 to 14.9)	6.3 (3.1 to 9.6)	4.9 (0.2 to 9.5)
Early phase	12.1 (8.5 to 15.7)	13.0 (9.1 to 16.9)	-0.8 (-6.1 to 4.5)
Late phase	7.2 (3.4 to 11.0)	1.5 (-2.3 to 5.3)	5.7 (0.2 to 11.2)
**Sensitivity analysis†**			
Overall	5.3 (0.6 to 10.0)	-0.1 (-5.0 to 4.8)	5.4 (0.7 to 10.0)
Early phase	17.9 (14.0 to 21.9)	17.8 (15.8 to 19.7)	0.2 (-5.0 to 5.3)
Late phase	3.5 (-0.5 to 7.6)	-2.2 (-6.2 to 1.7)	5.8 (0.3 to 11.2)

* The least-squares mean changes and between-group differences were adjusted for baseline value, disease phase and the correlation between eyes of the same patient, with the use of the generalized estimating equation.

† The least-squares mean changes and between-group differences were adjusted for baseline value, disease phase, the correlation between eyes of the same patient, peripheral iridotomy, trabeculectomy, and cataract phacoemulsification and intraocular lens implantation, with the use of the generalized estimating equation.

Supplementary Table 8. Sensitivity Analysis of Adjustment for Full Covariates

Primary Outcome	Cyclosporine-Corticosteroids (95% CI) N = 56	Adalimumab-Corticosteroids (95% CI) N = 54	Difference (95% CI)
**Primary analysis***			
Overall	11.2 (7.5 to 14.9)	6.3 (3.1 to 9.6)	4.9 (0.2 to 9.5)
Early phase	12.1 (8.5 to 15.7)	13.0 (9.1 to 16.9)	-0.8 (-6.1 to 4.5)
Late phase	7.2 (3.4 to 11.0)	1.5 (-2.3 to 5.3)	5.7 (0.2 to 11.2)
**Sensitivity analysis†**			
Overall	15.6 (9.6 to 21.6)	10.7 (5.0 to 16.5)	4.9 (0.6 to 9.2)
Early phase	11.6 (9.7 to 13.5)	11.6 (9.5 to 13.7)	-0.1 (-2.8 to 2.7)
Late phase	13.0 (6.8 to 19.2)	7.0 (1.1 to 12.9)	6.0 (0.9 to 11.1)

* The least-squares mean changes and between-group differences were adjusted for baseline value, disease phase and the correlation between eyes of the same patient, with the use of the generalized estimating equation.

† The least-squares mean changes and between-group differences were adjusted for baseline value, disease phase, the correlation between eyes of the same patient, cumulative prednisone dose, peripheral iridotomy, trabeculectomy, cataract phacoemulsification and intraocular lens implantation, age, sex, baseline body mass index, baseline central macular thickness and baseline visual field index, with the use of the generalized estimating equation.

Supplementary Table 9. Sensitivity Analysis of Modified Intention-To-Treat Analysis

Primary Outcome	Cyclosporine-Corticosteroids (95% CI)	Adalimumab-Corticosteroids (95% CI)	Difference (95% CI)
**Primary analysis – intention-to-treat population***			
Total no. of patients	56	54	-
Overall	11.2 (7.5 to 14.9)	6.3 (3.1 to 9.6)	4.9 (0.2 to 9.5)
Early phase	12.1 (8.5 to 15.7)	13.0 (9.1 to 16.9)	-0.8 (-6.1 to 4.5)
Late phase	7.2 (3.4 to 11.0)	1.5 (-2.3 to 5.3)	5.7 (0.2 to 11.2)
**Sensitivity analysis – modified intention-to-treat population†**			
Total no. of patients	43	41	-
Overall	13.4 (9.0 to 17.9)	7.2 (3.3 to 11.2)	6.2 (0.3 to 12.1)
Early phase	14.4 (11.9 to 16.9)	14.9 (11.8 to 18.0)	-0.5 (-4.5 to 3.6)
Late phase	8.3 (3.5 to 13.0)	1.8 (-3.4 to 6.9)	6.5 (-0.7 to 13.7)

* Intention-to-treat population included all participants who underwent randomization. Missing values on the primary outcome were imputed with the last observation carried forward.

† Modified intention-to-treat (mITT) population included all participants who underwent randomization, with the exception of those who did not start any assigned treatment and those who did not have any primary outcome data at week 26. No data imputation was performed for mITT analysis. The mITT analysis eventually excluded 26 patients who withdrew or were lost to follow-up before week 26 from the ITT population.

Supplementary Table 10. Sensitivity Analysis of Limited to Higher ETDRS Score

Primary Outcome	Cyclosporine-Corticosteroids (95% CI)	Adalimumab-Corticosteroids (95% CI)	Difference (95% CI)
**Primary analysis***			
Total no. of eyes	112	108	-
Overall	11.2 (7.5 to 14.9)	6.3 (3.1 to 9.6)	4.9 (0.2 to 9.5)
Early phase	12.1 (8.5 to 15.7)	13.0 (9.1 to 16.9)	-0.8 (-6.1 to 4.5)
Late phase	7.2 (3.4 to 11.0)	1.5 (-2.3 to 5.3)	5.7 (0.2 to 11.2)
**Sensitivity analysis†**			
Total no. of eyes	103	100	-
Overall	8.4 (5.3 to 11.5)	5.8 (2.7 to 9.0)	2.6 (-1.9 to 7.1)
Early phase	9.8 (5.8 to 13.7)	11.0 (7.2 to 14.9)	-1.3 (-6.8 to 4.3)
Late phase	5.4 (1.4 to 9.3)	1.6 (-2.4 to 5.5)	3.8 (-1.8 to 9.5)

* The analysis included eyes of all patients included in the intention-to-treat population.

† The analysis only included eyes with an ETDRS score of 20 letters or greater measured at baseline.

Supplementary Table 11. Sensitivity Analysis of Multiple Imputation

Primary Outcome	Cyclosporine-Corticosteroids (95% CI) N = 56	Adalimumab-Corticosteroids (95% CI) N = 54	Difference (95% CI)
**Primary analysis***			
Overall	11.2 (7.5 to 14.9)	6.3 (3.1 to 9.6)	4.9 (0.2 to 9.5)
Early phase	12.1 (8.5 to 15.7)	13.0 (9.1 to 16.9)	-0.8 (-6.1 to 4.5)
Late phase	7.2 (3.4 to 11.0)	1.5 (-2.3 to 5.3)	5.7 (0.2 to 11.2)
**Sensitivity analysis – not including treatment group as a predictor†**			
Overall	12.7 (8.7 to 16.7)	8.5 (4.5 to 12.6)	4.1 (-1.2 to 9.5)
Early phase	14.9 (9.7 to 20.1)	15.0 (10.5 to 19.4)	-0.1 (-6.8 to 6.6)
Late phase	7.7 (3.4 to 12.0)	3.2 (-1.7 to 8.0)	4.5 (-1.9 to 10.9)
**Sensitivity analysis – including treatment group as a predictor‡**			
Overall	13.3 (9.2 to 17.4)	8.1 (4.3 to 11.9)	5.2 (-0.1 to 10.4)
Early phase	15.7 (9.9 to 21.4)	14.5 (10.1 to 18.8)	1.2 (-5.7 to 8.0)
Late phase	8.2 (3.9 to 12.5)	2.7 (-1.9 to 7.4)	5.5 (-0.8 to 11.8)
**Sensitivity analysis – including follow-up data as predictors§**			
Overall	12.6 (8.7 to 16.4)	7.0 (3.4 to 10.7)	5.5 (0.4 to 10.7)
Early phase	14.1 (9.7 to 18.4)	13.8 (9.6 to 18.0)	0.2 (-5.6 to 6.1)
Late phase	7.9 (3.9 to 11.9)	1.7 (-2.9 to 6.3)	6.2 (0.1 to 12.4)

* Missing values on the primary outcome were imputed with the last observation carried forward in the intention-to-treat population.

† This analysis was prespecified. Missing values on the primary outcome were predicted in 50 imputed datasets with the multiple imputation in the intention-to-treat population. Baseline information about age, sex, body mass index, disease phase, best-corrected visual acuity ETDRS score, central macular thickness and visual field index was used for multiple imputations. The difference and the 95% CI were combined with the Rubin's rule from 50 imputation datasets.

‡ This analysis was prespecified. Missing values on the primary outcome were predicted in 50 imputed datasets with the multiple imputation in the intention-to-treat population. Treatment group and baseline

information about age, sex, body mass index, disease phase, best-corrected visual acuity ETDRS score, central macular thickness and visual field index were used for multiple imputations. The difference and the 95% CI were combined with the Rubin's rule from 50 imputation datasets.

§ This analysis was performed post-hoc. Missing values on the primary outcome were predicted in 50 imputed datasets with the multiple imputation in the intention-to-treat population. Treatment group, baseline information about age, sex, body mass index, disease phase, best-corrected visual acuity ETDRS score, central macular thickness and visual field index, and available data of each follow-up visit on best-corrected visual acuity ETDRS score and central macular thickness were used for multiple imputations. The difference and the 95% CI were combined with the Rubin's rule from 50 imputation datasets.

Supplementary Table 12. Sensitivity Analysis of Excluding Effects of Step-up Treatment

Primary Outcome	Cyclosporine-Corticosteroids (95% CI) N = 56	Adalimumab-Corticosteroids (95% CI) N = 54	Difference (95% CI)
**Primary analysis***			
Overall	11.2 (7.5 to 14.9)	6.3 (3.1 to 9.6)	4.9 (0.2 to 9.5)
Early phase	12.1 (8.5 to 15.7)	13.0 (9.1 to 16.9)	-0.8 (-6.1 to 4.5)
Late phase	7.2 (3.4 to 11.0)	1.5 (-2.3 to 5.3)	5.7 (0.2 to 11.2)
**Sensitivity analysis†**			
Overall	9.2 (6.3 to 12.2)	6.2 (3.0 to 9.3)	3.1 (-1.2 to 7.3)
Early phase	10.1 (6.5 to 13.7)	12.3 (7.8 to 16.7)	-2.1 (-7.9 to 3.6)
Late phase	5.8 (2.2 to 9.5)	1.6 (-2.2 to 5.3)	4.2 (-1.1 to 9.6)

* The least-squares mean changes and between-group differences were adjusted for baseline value, disease phase and the correlation between eyes of the same patient, with the use of the generalized estimating equation.

† The outcome data were assigned as the visual acuity value at the clinic visit when the step-up treatment was initiated in the sensitivity analysis.

Supplementary Table 13. Sensitivity Analysis of Inverse Probability Weighting for Non-Missing Rate

Primary Outcome	Cyclosporine-Corticosteroids (95% CI)	Adalimumab-Corticosteroids (95% CI)	Difference (95% CI)
**Primary analysis***			
Overall	11.2 (7.5 to 14.9)	6.3 (3.1 to 9.6)	4.9 (0.2 to 9.5)
Early phase	12.1 (8.5 to 15.7)	13.0 (9.1 to 16.9)	-0.8 (-6.1 to 4.5)
Late phase	7.2 (3.4 to 11.0)	1.5 (-2.3 to 5.3)	5.7 (0.2 to 11.2)
**Sensitivity analysis†**			
Overall	13.4 (9.0 to 17.9)	8.4 (4.7 to 12.1)	5.0 (-0.9 to 10.9)
Early phase	16.8 (14.6 to 19.0)	18.2 (15.6 to 20.8)	-1.4 (-4.9 to 2.1)
Late phase	5.5 (0.4 to 10.7)	1.5 (-3.5 to 6.5)	4.0 (-3.4 to 11.5)

* The primary analysis was performed in the intention-to-treat population.

† The sensitivity analysis was performed with a weighted model in the modified intention-to-treat population which included all patients in the intention-to-treat population with exception of those who withdrew or were lost to follow-up before week 26. The weight of each participant was given by the inverse of the predicted probability of being a complete case (non-missing rate).

Supplementary Table 14. Sensitivity Analysis of Working Correlation Structures in Generalized Estimating Equation

Primary Outcome*	Cyclosporine-Corticosteroids (95% CI) N = 56	Adalimumab-Corticosteroids (95% CI) N = 54	Difference (95% CI)
**Primary analysis – Independent working correlation matrix**			
Overall	11.2 (7.5 to 14.9)	6.3 (3.1 to 9.6)	4.9 (0.2 to 9.5)
Early phase	12.1 (8.5 to 15.7)	13.0 (9.1 to 16.9)	-0.8 (-6.1 to 4.5)
Late phase	7.2 (3.4 to 11.0)	1.5 (-2.3 to 5.3)	5.7 (0.2 to 11.2)
**Sensitivity analysis – Exchangeable working correlation matrix**			
Overall	11.2 (7.5 to 14.9)	6.3 (3.1 to 9.6)	4.9 (0.2 to 9.6)
Early phase	12.3 (8.8 to 15.8)	12.8 (8.8 to 16.8)	-0.5 (-5.7 to 4.6)
Late phase	7.2 (3.4 to 11.0)	1.4 (-2.5 to 5.4)	5.8 (0.2 to 11.4)
**Sensitivity analysis – Unstructured working correlation matrix**			
Overall	11.2 (7.5 to 14.9)	6.3 (3.1 to 9.6)	4.9 (0.2 to 9.6)
Early phase	12.3 (8.8 to 15.8)	12.8 (8.8 to 16.8)	-0.5 (-5.7 to 4.6)
Late phase	7.2 (3.4 to 11.0)	1.4 (-2.5 to 5.4)	5.8 (0.2 to 11.4)

* The least-squares mean changes and between-group differences were adjusted for baseline value, disease phase and the correlation between eyes of the same patient, with the use of the generalized estimating equation.

Supplementary Table 15. Full List of Adverse Events

Event	Cyclosporine-Corticosteroids (N = 56)		Adalimumab-Corticosteroids (N = 54)	
	No. of events	Events/100 person-years	No. of events	Events/100 person-years
**Adverse event possibly associated with trial drugs**	314	1286.9	257	1000.0
Alanine aminotransferse increased	9	36.9	8	31.1
Aspartate aminotransferase increased	3	12.3	2	7.8
Alkaline phosphatase increased	5	20.5	0	0.0
Blood bilirubin increased	17	69.7	9	35.0
Albumin decreased	1	4.1	4	15.6
Albumin globulin ratio increased	2	8.2	5	19.5
Albumin globulin ratio decreased	1	4.1	2	7.8
Blood lactate dehydrogenase increased	9	36.9	3	11.7
Creatinine increased	13	53.3	7	27.2
Glomerular filteration rate decreased	11	45.1	6	23.3
Gamma-glutamyltransferase increased	2	8.2	2	7.8
Blood urea nitrogen increased	13	53.3	9	35.0
C-reactive protein increased	4	16.4	5	19.5
Erythrocytes sedimentation rate increased	10	41.0	9	35.0

Event	Cyclosporine-Corticosteroids (N = 56)		Adalimumab-Corticosteroids (N = 54)	
	No. of events	Events/100 person-years	No. of events	Events/100 person-years
Hemoglobin increased	8	32.8	7	27.2
Lymphocyte count increased	25	102.5	37	144.0
Lymphocyte count decreased	3	12.3	0	0.0
Eosinophilia	4	16.4	2	7.8
Platelet count decreased	0	0.0	1	3.9
Leukocytosis	37	151.6	38	147.9
Hyperglycemia	15	61.5	12	46.7
Hypercalcemia	6	24.6	3	11.7
Hypomagnesemia	7	28.7	3	11.7
Hypophosphatemia	1	4.1	1	3.9
Hypocalcemia	3	12.3	0	0.0
Hyperuricemia	19	77.9	16	62.3
Hypertension (systolic BP≥120 mmHg or diastolic BP≥80 mmHg)	20	82.0	17	66.1
Malaise	4	16.4	0	0.0
Insomnia	4	16.4	2	7.8
Injection site reaction	0	0.0	4	15.6
Fever	0	0.0	1	3.9
Chest tightness	5	20.5	0	0.0
Chest pain	5	20.5	0	0.0
Arthragia	13	53.3	0	0.0
Toothache	8	32.8	0	0.0
Dizziness	8	32.8	0	0.0
Headeache	0	0.0	3	11.7

Event	Cyclosporine-Corticosteroids (N = 56)		Adalimumab-Corticosteroids (N = 54)	
	**No. of events**	**Events/100 person-years**	**No. of events**	**Events/100 person-years**
Abdominal pain	0	0.0	5	19.5
Stomach pain	2	8.2	0	0.0
Diarrhea	3	12.3	5	19.5
Pneumonitis	1	4.1	1	3.9
Folliculitis	0	0.0	1	3.9
Urticaria	0	0.0	5	19.5
Rash maculo-papular	3	12.3	4	15.6
Pruritus cutaneous	1	4.1	5	19.5
Rash acneiform	1	4.1	0	0.0
Shingles	1	4.1	1	3.9
Dry eye	6	24.6	0	0.0
Corneal ulcer	0	0.0	4	15.6
Watering eyes	0	0.0	8	31.1
Oral ulceration	1	4.1	0	0.0
**Adverse event possibly associated with VKH disease**	70	286.9	115	447.5
Eye redness	5	20.5	4	15.6
Eye pain	3	12.3	4	15.6
Flashing lights	2	8.2	2	7.8
Glaucoma	0	0.0	3	11.7
Photophobia	3	12.3	2	7.8
Cataract	15	61.5	14	54.5
Iris synechiae	4	16.4	16	62.3

**Event**	**Cyclosporine-Corticosteroids (N = 56)**		**Adalimumab-Corticosteroids (N = 54)**	
	**No. of events**	**Events/100 person-years**	**No. of events**	**Events/100 person-years**
Ocular hypertension	31	127.0	61	237.4
Macular edema	4	16.4	2	7.8
Retinal detachment	3	12.3	7	27.2
**Adverse event leading to discontinuation of trial drugs**	3	12.3	2	7.8
Alanine aminotransferse increased	2	8.2	0	0.0
Pneumonitis	1	4.1	0	0.0
Shingles	0	0.0	1	3.9
Corneal ulcer	0	0.0	1	3.9

Supplementary Table 16. Eligibility Criteria

Inclusion Criteria

a. Subjects of either sex aged ≥ 18 years at the time of signing the informed consent document.

b. Diagnosed with Vogt-Koyanagi-Harada disease.

c. An active disease status:

Requiring chronic oral prednisone (≥ 20 mg or equivalent) for the treatment for intraocular inflammation;

And/OR

Having an active inflammation or having documented history of experiencing an active inflammation within the last 90 days as defined by the presence of at least one of the following parameters in either eye:

(1) ≥ 2+ anterior chamber cells;

(2) ≥ 2+ vitreous haze;

(3) Presence of active inflammatory choroidal or retinal lesions detected by optical coherence tomography (OCT) or fluorescein fundus angiography (FFA).

d. Media clarity, pupillary dilatation and subject cooperation judged by the study Investigator for proper viewing of the posterior segment in at least one eye.

e. Able and willing to consistently use effective contraceptive methods in order to successfully prevent pregnancy.

f. Able and willing to self-administer subcutaneous injections or have a qualified condition available to administer subcutaneous injections.

g. Able to adhere to the study visit schedule and other protocol requirements.

Exclusion Criteria

a. Visual acuity of hand motions or worse in the better-seeing eye.

b. Previous exposure to anti-TNF therapy.

c. Using > 1 systemic immunosuppressive therapy (not including corticosteroids) within the last 28 days.

d. Uncontrolled intraocular pressure with ≥ 2 glaucoma medications or evidence of optic nerve injury.

e. Chronic hypotony (IOP < 5 mm Hg for > 3 months) in both eyes.

f. Received intraocular or periocular corticosteroids in the past 3 months.

g. Received Ozurdex® (dexamethasone implant) in the past 6 months.

h. Received Retisert® (glucocorticosteroid implant) within 3 years prior to the Screening.

i. Proliferative or severe non-proliferative diabetic retinopathy or clinically significant macular edema due to diabetic retinopathy.

j. Neovascular/wet age-related macular degeneration.

k. History of moderate to severe congestive heart failure (NYHA class III or IV) or recent cerebrovascular accident.

l. Current or history of demyelinating disease such as multiple sclerosis.

m. Malignancy or history of malignancy.

n. White blood cells $< 3 \times 10^9$/L at the Screening.

o. Platelet count $< 100 \times 10^9$/L or $> 1200 \times 10^9$/L at the Screening.

p. Hemoglobin < 8 g/dL at the Screening.

q. Abnormal alanine aminotransferase (ALT) and/or aspartate aminotransferase (AST) ≥ 2 times the upper limit of normal for the lab at the Screening.

r. Abnormal serum creatinine ≥ 2 times the upper limit of normal for the lab at the Screening.

The above laboratory tests will be allowed to be repeated 1 time if, in the Investigator's clinical judgment, there is a reasonable possibility of the repeat tests not meeting the exclusion values.

s. Systemic or opportunistic fungal infection.

t. Having active tuberculosis, HIV infection, syphilis, or hepatitis B or C based on laboratory data and clinical judgment by the study Investigator.

u. Known active current or history of recurrent infections (including but not limited to herpes zoster, histoplasmosis, coccidiomycosis, but excluding onychomycosis) or any major episode of infection requiring hospitalization or treatment with intravenous injection or oral antibiotics within 4 weeks of the Screening.

Beyond the tests listed above, the remainder of the work-up is at the discretion of the investigator and should be tailored to the clinical situation. Distinguishing between infectious and non-infectious uveitis is part of standard of care and should be dictated by the patient's clinical

exam, but at minimum, all patients must have testing for tuberculosis, syphilis and hepatitis prior to enrollment.

v. Prior history of suicide attempt at any time in the subject's lifetime or major psychiatric illness requiring hospitalization within 3 years prior to the Screening.

w. Medical problems or drug or alcohol dependence problems sufficient to prevent adherence to treatment and study procedures.

x. Scheduled surgery or other interventions that would interrupt the subject's participation in the study.

y. Pregnancy or current breast-feeding.

z. Enrolled in other clinical trials.

aa. Any other condition which, in the opinion of the Investigator, would put the subject at risk by participation in the protocol.

Supplementary Table 17. Device of Ophthalmic Auxiliary Examination

Device	Manufacturer	Instrument Model
Optical coherence tomography (OCT)	Heidelberg, Germany	Spectralis OCT
Fluorescein fundus angiography (FFA)	Heidelberg, Germany	Spectralis HRA
Fundus photography	Carl Zeiss Meditec, Inc., Germany	500
Visual field analyzer	Carl Zeiss Meditec, Inc., Germany	750i
Non-contact tonometer	Topcon, USA	CT-1

Supplementary References

1. Standardization of Uveitis Nomenclature (SUN) Working Group. Classification Criteria for Vogt-Koyanagi-Harada Disease. *Am J Ophthalmol* 228, 205-211 (2021).
2. Yang, P., *et al.* Development and evaluation of diagnostic criteria for Vogt-Koyanagi-Harada disease. *JAMA Ophthalmol* 136, 1025-1031 (2018).
3. Narduzzi, S., Golini, M.N., Porta, D., Stafoggia, M. & Forastiere, F. [Inverse probability weighting (IPW) for evaluating and "correcting" selection bias]. *Epidemiol Prev* 38, 335-341 (2014).

Conventional versus Biological Treatment for Vogt-Koyanagi-Harada Disease

A Randomized, Open-label, Blinded-endpoint, Non-inferiority Trial

Study Protocol

Version Number: 3.0

The First Affiliated Hospital of Chongqing Medical University,

Chongqing Key Laboratory of Ophthalmology and Chongqing Eye Institute,

Chongqing, China

Protocol Title: Conventional versus Biological Treatment for Vogt-Koyanagi-Harada Disease: A Randomized, Open-label, Blinded-endpoint, Non-inferiority Trial

Trial Code/Short Title: VKH-I

Version Number: 3.0

Protocol Date: December 20th, 2021

Site: Single center (The First Affiliated Hospital of Chongqing Medical University)

Grant: Chongqing Key Laboratory of Ophthalmology (CSTC, 2008CA5003) and National Natural Science Foundation of China

Back-up 24 Hour Global Emergency Contact Call: [redacted]

The back-up 24 hour global emergency contact call should only be used if you are not able to reach the Clinical Research Physician(s) or Study Coordinator(s) for emergency calls.

Document Revision History

Version

1. Protocol 1.0, January 1st, 2021

2. Protocol 2.0, May 15th, 2021

Revision in 6.3 Conventional Treatment Regimen

"If a specific contraindication to cyclosporine exists, subjects will be orally treated with chlorambucil at a dose of 0.05-0.1 mg/kg/day throughout the trial" added.

The dosage and duration of treatment with chlorambucil changed from 0.1 mg/kg/day to 0.05-0.1 mg/kg/day.

Protocol 3.0, December 20th, 2021

Revision in 7.2 Secondary Outcomes

"Change in BCVA ETDRS letter score from Baseline to the end of 2 months and 4 months" deleted.

"Proportion achieving a ≥ 15 ETDRS letter improvement (appreciable visual gain), a ≥ 10 letter improvement at the end of 2 months, 4 months and 6 months" changed to "Proportion achieving a ≥ 15 ETDRS letter improvement (appreciable visual gain) at the end of 26 weeks".

"Change in visual field mean deviation score from Baseline to the end of 26 weeks" changed to "Change in visual field indices (visual field index, mean deviation and pattern standard deviation) from Baseline to the end of 26 weeks".

"Change in anterior chamber cells from the Baseline to the end of 26 weeks" and "Change in vitreous haze from the Baseline to the end of 26 weeks" altogether changed to "Proportion achieving an inactive uveitis (an anterior chamber cell grade of 0.5+ or less, a vitreous haze grade of 0.5+ or less, and no active inflammatory choroidal or retinal lesions) in both eyes at the end of 26 weeks".

"Changes in OCT anatomical features from the Baseline to the end of 26 weeks" changed to "Changes in OCT anatomical features (central macular thickness) from the Baseline to the end of 26 weeks".

Grant support to the trial changed to Chongqing Key Laboratory of Ophthalmology (CSTC, 2008CA5003) and National Natural Science Foundation of China.

CONTENTS

CONTENTS 5
ABBREVIATIONS 8
1 Introduction 9
1.1 Background 9
1.2 Benefits and Risks 9
2 Objectives 11
2.1 Trial Objectives 11
2.2 Primary Objective 11
2.3 Secondary Objectives 11
2.4 Safety Objectives 11
3 Study Design 12
3.1 Overall Design 12
3.2 Study Schematic 12
3.3 Study Timeline 13
4 Eligibility Criteria 14
4.1 Inclusion Criteria 14
4.2 Exclusion Criteria 15
4.3 Re-screening of Patients 17
5 Study Procedures 18
5.1 Informed Consent Procedure 18
5.2 Eligibility Evaluation 18
5.3 Randomization Assignment and Masking 18
5.4 Baseline and Subsequent Assessments 19
5.5 Withdrawn and Lost to Follow-up 20
5.6 Study Assessments and Methods 21
5.7 Study Schedule Overview 25
6 Treatment Plan 27

6.1 Stratification ... 27

6.2 Corticosteroids ... 27

6.3 Conventional Treatment Regimen ... 27

6.4 Biological Treatment Regimen ... 28

6.5 Other Adjunctive Treatments ... 29

6.6 Prohibited Therapy ... 29

7 Outcome Assessments ... 31

7.1 Primary Outcome ... 31

7.2 Secondary Outcomes ... 31

7.3 Safety Outcomes ... 31

7.4 Pre-specified Subgroup Analyses ... 32

7.5 Cost Effectiveness ... 32

8 Adverse Events ... 33

8.1 Adverse Event Reporting ... 33

8.2 Serious Adverse Events ... 33

8.3 Severity Assessments ... 34

8.4 Causality Assessments ... 34

8.5 Uveitis-Related Events ... 34

8.6 Pregnancy ... 35

8.7 Management ... 36

9 Data Collection and Management ... 37

9.1 Confidentiality ... 37

9.2 Source and Data Collection ... 37

9.3 Quality Assurance ... 37

9.4 Database Lock ... 38

9.5 Archiving ... 38

10 Statistical Considerations ... 39

10.1 Study Hypothesis ... 39

10.2 Sample Size Calculation 39
10.3 Data Analyses 39
10.4 Interim Analysis 40
11 Ethical Considerations 41
REFERENCES 42
Appendix I 43
Appendix II 45
Appendix III 46

ABBREVIATIONS

AE	Adverse event
ALT	Alanine aminotransferase
AST	Aspartate aminotransferase
BCVA	Best corrected visual acuity
BUN	Blood Urea Nitrogen
CFDA	China Food and Drug Administration
CRF	Case report form
FFA	Fluorescein fundus angiography
EQ-5D	EuroQol-5D Questionnaire
ETDRS	Early treatment diabetic retinopathy study
MCID	Minimal clinically important difference
NEI	National Eye Institute
NYHA	New York Heart Association
OCT	Optical coherence tomography
PRO(s)	Patient reported outcome(s)
QALY(s)	Quality adjusted life year(s)
SUN	Standardization of Uveitis Nomenclature Working Group
TNF	Tumor necrosis factor
UBM	Ultrasound biomicroscope
VFQ-25	Visual functioning questionnaire 25
VKH	Vogt-Koyanagi-Harada

1 Introduction

1.1 Background

Vogt-Koyanagi-Harada (VKH) disease is an immune-mediated disorder characterized by bilateral uveitis frequently associated with neurological (meningeal), auditory, and integumentary manifestations.[1] Although the etiology and pathogenesis of VKH disease need to be further elucidated, it is widely accepted that the clinical manifestations are caused by an autoimmune response directed against melanin associated antigens in the target organs, i.e. the eye, inner ear, meninges and skin.[2] The most frequent complaint in patients with VKH disease is a rapid decrease in vision in one or both eyes following a transiently blurred vision.[3]

Currently, the treatment for VKH disease has not been extensively evaluated in prospective clinical trials and the treatment is generally empirical in clinical practice. Glucocorticosteroids and conventional immunosuppressive agents such as cyclosporine are still the most often prescribed drugs for VKH disease.[4-6] Adalimumab, a recombinant human monoclonal antibody that specifically binds and inhibits a proinflammatory cytokine, tumor necrosis factor (TNF), has been demonstrated the efficacy for noninfectious uveitis and yielded promising results in the treatment of uveitis refractory to conventional therapy.[7,8] Recently, it has been recognized that VKH disease is largely heterogeneous and treatment for the disease may be individualized.[3,4] It remains unclear whether an individualized, conventional drug based treatment strategy is as effective as the biological treatment for VKH disease.

Therefore, we aim to initiate a clinical trial to compare the clinical and cost effectiveness between the conventional and biological treatment strategies for VKH disease.

1.2 Benefits and Risks

This trial will determine if there are any differences in clinical and cost effectiveness, safety profile and other outcomes between conventional drugs and biological drugs in a randomized trial, of which results will guide clinical decision-making in the treatment for VKH disease.

This trial may incur some risks to subjects, mostly related to the common side effects for study drugs, such infection, allergy, injection-site reaction. Nevertheless, the study drugs had been evaluated for the treatment of other diseases in phase 3 clinical trials,[7,8] which suggested that these study drugs were generally safe.

2 Objectives

2.1 Trial Objectives

The objective is to compare the clinical and cost effectiveness of an individualized treatment strategy based on conventional immunosuppressive drugs with that of a standardized treatment strategy based on the biological drug, adalimumab in Vogt-Koyanagi-Harada disease. We aim to determine if the conventional treatment is as effective as the biological treatment in treating visual loss in Vogt-Koyanagi-Harada disease and whether they have an equivalent side effect profile.

2.2 Primary Objective

To determine whether the conventional treatment is non-inferior to the biological treatment in improving the best corrected visual acuity (BCVA) at 26 weeks among patients with Vogt-Koyanagi-Harada disease.

2.3 Secondary Objectives

To compare efficacy, clinical outcomes, cost effectiveness and patient reported outcomes (PROs) between the conventional and biological treatment groups in Vogt-Koyanagi-Harada disease.

2.4 Safety Objectives

To evaluate the safety and tolerability of the conventional versus biological treatment for Vogt-Koyanagi-Harada disease.

3 Study Design

3.1 Overall Design

This is an investigator-initiated, randomized, open-label, blinded-endpoint, non-inferiority clinical trial to compare the clinical and cost effectiveness of conventional and biological treatment strategies/regimens for subjects with Vogt-Koyanagi-Harada disease and who have an active inflammation within the last 90 days in any eye and/or require chronic oral prednisone ≥ 20 mg/day for the treatment for intraocular inflammation.

Approximately 110 eligible subjects will be randomized 1:1 (55 subjects per group) to receive one of two treatment strategy arms using the disease phase (early or late phase VKH) as the stratification factor. In the conventional treatment arm, subjects will receive an individualized regimen based on prednisone primarily combined with cyclosporine. In the biological treatment arm, subjects will receive a standardized regimen of prednisone and adalimumab throughout the trial. All participants will be followed up for 26 weeks since randomization.

The primary outcome will be the difference in mean Early Treatment Diabetic Retinopathy Study (ETDRS) BCVA letter score at 26 weeks. The minimal clinically important difference (MCID) for change in letters read is 7 letters, based on clinical trial results of pivotal trials of treatments in uveitis and wet macular degeneration.[9] This trial aims to test whether the mean change in ETDRS BCVA letters from randomization to Week 26 is not different by more than a margin of 7 letters.

3.2 Study Schematic

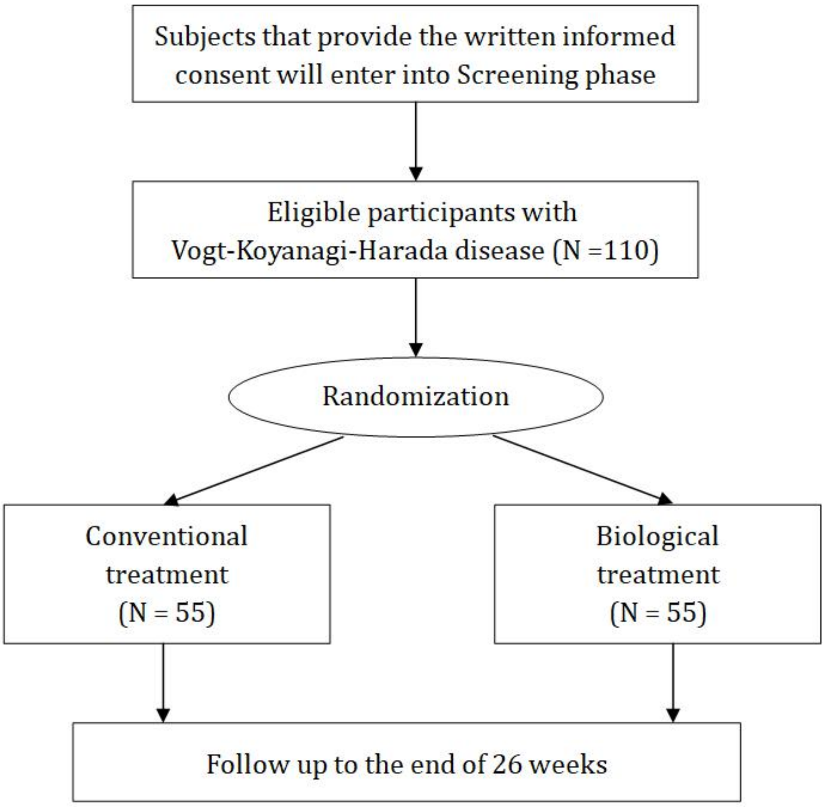

3.3 Study Timeline

Expected time for trial start: February 2021

Expected completion time of enrollment: September 2021

Expected time for end of trial: March 2022

The end of trial refers to the date of the last visit of the last subject to complete the study, or the date of receipt of the last data point from the last subject that is required for primary, secondary and/or exploratory analysis, as pre-specified in the protocol and/or the Statistical Analysis Plan, whichever is the later date.

Expected completion time of primary analysis: August 2022

4 Eligibility Criteria

4.1 Inclusion Criteria

A subject will be eligible for study participation if she/he meets all of the following inclusion criteria:

a. Subjects of either sex aged ≥ 18 years at the time of signing the informed consent document.

b. Diagnosed with Vogt-Koyanagi-Harada disease. See Diagnostic Criteria for Vogt-Koyanagi-Harada disease in Appendix I. [10]

c. An active disease status:

   Requiring chronic oral prednisone (≥ 20 mg or equivalent) for the treatment for intraocular inflammation;

   And/OR

   Having an active inflammation or having documented history of experiencing an active inflammation within the last 90 days as defined by the presence of at least one of the following parameters in either eye:

   (1) ≥ 2+ anterior chamber cells (See SUN criteria in Appendix II);

   (2) ≥ 2+ vitreous haze (See NEI/SUN criteria in Appendix III);

   (3) Presence of active inflammatory choroidal or retinal lesions detected by optical coherence tomography (OCT) or fluorescein fundus angiography (FFA).

d. Media clarity, pupillary dilatation and subject cooperation judged by the study Investigator for proper viewing of the posterior segment in at least one eye.

e. Able and willing to consistently use effective contraceptive methods in order to successfully prevent pregnancy.

f. Able and willing to self-administer subcutaneous injections or have a qualified condition available to administer subcutaneous injections.

g. Able to adhere to the study visit schedule and other protocol requirements.

4.2 Exclusion Criteria

The presence of any of the following will exclude a subject from the study enrollment:

a. Visual acuity of hand motions or worse in the better-seeing eye.

b. Previous exposure to anti-TNF therapy.

c. Using > 1 systemic immunosuppressive therapy (not including corticosteroids) within the last 28 days.

d. Uncontrolled intraocular pressure with ≥ 2 glaucoma medications or evidence of optic nerve injury.

e. Chronic hypotony (IOP < 5 mm Hg for > 3 months) in both eyes.

f. Received intraocular or periocular corticosteroids in the past 3 months.

g. Received Ozurdex® (dexamethasone implant) in the past 6 months.

h. Received Retisert® (glucocorticosteroid implant) within 3 years prior to the Screening.

i. Proliferative or severe non-proliferative diabetic retinopathy or clinically significant macular edema due to diabetic retinopathy.

j. Neovascular/wet age-related macular degeneration.

k. History of moderate to severe congestive heart failure (NYHA class III or IV) or recent cerebrovascular accident.

l. Current or history of demyelinating disease such as multiple sclerosis.

m. Malignancy or history of malignancy.

n. White blood cells $< 3 \times 10^9$/L at the Screening.

o. Platelet count $< 100 \times 10^9$/L or $> 1200 \times 10^9$/L at the Screening.

p. Hemoglobin < 8 g/dL at the Screening.

q. Abnormal alanine aminotransferase (ALT) and/or aspartate aminotransferase (AST) ≥ 2 times the upper limit of normal for the lab at the Screening.

r. Abnormal serum creatinine ≥ 2 times the upper limit of normal for the lab at the Screening.

The above laboratory tests will be allowed to be repeated 1 time if, in the Investigator's clinical judgment, there is a reasonable possibility of the repeat tests not meeting the exclusion values.

s. Systemic or opportunistic fungal infection.

t. Having active tuberculosis, HIV infection, syphilis, or hepatitis B or C based on laboratory data and clinical judgment by the study Investigator.

u. Known active current or history of recurrent infections (including but not limited to herpes zoster, histoplasmosis, coccidiomycosis, but excluding onychomycosis) or any major episode of infection requiring hospitalization or treatment with intravenous injection or oral antibiotics within 4 weeks of the Screening.

Beyond the tests listed above, the remainder of the work-up is at the discretion of the investigator and should be tailored to the clinical situation. Distinguishing between infectious and non-infectious uveitis is part of standard of care and should be dictated by the patient's clinical exam, but at minimum, all patients must have testing for tuberculosis, syphilis and hepatitis prior to enrollment.

v. Prior history of suicide attempt at any time in the subject's lifetime or major psychiatric illness requiring hospitalization within 3 years prior to the Screening.

w. Medical problems or drug or alcohol dependence problems sufficient to prevent adherence to treatment and study procedures.

x. Scheduled surgery or other interventions that would interrupt the subject's participation in the study.

y. Pregnancy or current breast-feeding.

z. Enrolled in other clinical trials.

aa. Any other condition which, in the opinion of the Investigator, would put the subject at risk by participation in the protocol.

4.3 Re-screening of Patients

Individuals that do not meet the above eligibility criteria may be re-screened a minimum of 4 weeks after the last screening visit.

5 Study Procedures

5.1 Informed Consent Procedure

Procedures for obtaining consent include explaining the patient's disease, prognosis, and treatment options, introducing the aims and methods of this trial, discussing the risks and benefits of participation and addressing the patient's questions and concerns Patients will be given at least 24 hours to consider taking part. The subject is assured that participation in this study is voluntary and he/she can withdraw at any time, without giving a reason. A signed informed consent will be obtained from the subject or their legally authorized representative before any study-related procedures are undertaken.

5.2 Eligibility Evaluation

The eligibility evaluation will be performed at the Screening. For detailed inclusion/exclusion criteria see Section 4. Also please see Section 5.7 for assessments at the Screening visit.

If a subject had any item of the results of *Tuberculosis screening, Hepatitis B, Hepatitis C, syphilis and Human immune deficiency virus screening*, *Clinical laboratory tests, Fundus photography, Optical coherence tomography (OCT), B ultrasonography,* and *Ultrasound biomicroscope (UBM)* within 30 days and the results of *Fundus fluorescence angiography (FFA)* within 60 days prior to Screening, and all protocol required documentation is available, this test does not need to be repeated for the items, provided nothing has changed in the subject's medical history to warrant a repeat test.

5.3 Randomization Assignment and Masking

After eligibility has been determined, assignment of a randomization takes place at the Baseline visit. Only subjects who meet all of the inclusion criteria and none of the

exclusion criteria are eligible for randomization into the treatment phase. Randomization will be designed to yield expected assignment ratio of 1:1 to the conventional treatment arm and the biological treatment arm. Randomization will be stratified according to the phase of disease (early or late phase VKH, See Section 6.1) and will be blocked to assure balance across treatment groups. Randomization list will have been generated by a Statistician using a computer and will be properly kept by a Designated Study Coordinator. Both this Statistician and the Study Coordinator will have no involvement in other parts of the trial. The Investigator who is designated as the Primary Treating Ophthalmologist and is responsible for the treatment/management of the subject and the evaluation of the adverse events in this trial will telephone the Designated Study Coordinator to obtained patient assignment information.

Throughout the trial, the treatment assignment will not be masked to the Primary Treating Ophthalmologist. This trial will designate several Investigators as Examining Ophthalmologists who will perform *Visual acuity testing, Tonometry, Perimetry, Fundus photography, Optical coherence tomography (OCT), Fundus fluorescence angiography (FFA), B ultrasonography* and *Ultrasound biomicroscope (UBM)*, and will be masked to treatment assignment to prevent bias in study outcomes.

5.4 Baseline and Subsequent Assessments

The Baseline visit becomes time-point zero from which all other visit dates are calculated. The Screening and the Baseline can be performed on the same day, if all necessary test results are available. The time interval between the Screening and the Baseline should be no more than 10 days. Given this relatively narrow time interval, assessments, auxiliary examinations and laboratory tests already performed in the Screening will not need to be repeated, of which results will be collected and recorded for the Baseline visit. Subjects will initiate the assigned treatment at the Baseline per protocol (See Section 6). Subsequently, subjects will have scheduled clinic visits at

Week 2, Month 2, and every 2 months, until the end of 6 months (Week 26). This trial allows flexible completion of assessment and treatment within the window. Please see for the full study schedule.

Despite the regular scheduled visits, subjects may have unscheduled visits, such as if they experience new ocular or systemic symptoms or adverse events. These additional visits do not fall within the study visit window. The Investigator may carry out other necessary procedures or examinations than those performed in scheduled visits.

5.5 Withdrawn and Lost to Follow-up

Subjects have the right to withdraw from the trial at any time and for any reason, without providing a reason. The investigator also has the right to withdraw participants from the study in the event of inter-current illness, adverse events, protocol violations or other reasons.

If the subject withdraws from the trial, efforts will be made to continue to obtain follow-up data, with the permission of the subject. They will be given appropriate treatment, but will not continue with scheduled study follow-ups unless they allow it.

Subjects will also be considered to have been withdrawn if they have died. If the death is considered to be related to the study drug, the subject will be declared serious adverse event, rather than withdrawn.

Missing a visit does not necessarily mean that a patient has been withdrawn from the study. Some subjects may miss study visits, or do not adhere to the treatment plan, or stop the treatment regimen by self, but they are willing to return for study visits and declare they are interested in further participation. For these subjects, efforts will be made to bring them back as soon as possible. These subjects will be encouraged to return for subsequent study visits according to the protocol, regardless of whether or not they are taking study medication or adhering to the treatment plan. Also, they will be encouraged to resume the assigned treatment if appropriate.

For those subjects fail to attend study visits without stating an intention to withdraw from the study, the Investigator should contact the subject through telephone calls as soon as possible. If contact cannot be established up to the end of the 6-month follow-up period, the subject should be considered as “lost to follow-up” on the date of last contact.

5.6 Study Assessments and Methods

General information and medical history

Complete demographics and medical history will be obtained by the unmasked Primary Treating Ophthalmologist from each subject including date of birth, gender, ethnic background, education background, marriage status, occupation and history of tobacco and alcohol use, any prior ocular history and treatment.

Questionnaires

The EuroQol-5D Questionnaire (EQ-5D) will be used to evaluate the generic quality of life of the subject and The Visual Functioning Questionnaire (VFQ-25) will be used to measure the subject’s vision-related quality of life. Considering that the subject has impaired vision and may be unable to complete the full battery of questionnaires, these questionnaires will read aloud to subjects by a Study Coordinator who is masked to the treatment assignment. The Study Coordinator should never rephrase or interpret questions for the subject, and will record the subject’s answers on the appropriate questionnaires in their original form. These questionnaires should be assessed prior to discussion of the day’s examination results with the participant, to avoid biasing subjective responses by the day’s examination findings.

Vital signs/weight/height

These examinations include sitting blood pressure, heart rate, respiratory rate, body temperature, weight and height and will be performed by a masked Study Nurse.

Physical examination

At the Screening/Baseline, medically qualified, unmasked personnel who routinely do a complete physical exam will perform this assessment. The physical examination at the Baseline visit will serve as the baseline physical examination for the entire study. At all other visits, a symptom directed physical exam will be performed either by the Primary Treating Ophthalmologist or the medically qualified personnel who perform the complete physical exam. Abnormalities noted after the treatment should be evaluated and documented by the Investigator as to whether or not these are adverse events.

Visual acuity testing

With the appropriate corrective lenses based on that subject's refraction, the best corrected visual acuity (BCVA) will be measured using an ETDRS chart using standard operating procedures. The subject’s presenting visual acuity without refraction will also be evaluated using a Logarithmic visual acuity chart. Visual acuity will be obtained prior to pupil dilation. Visual acuity testing will be performed by a masked Examining Ophthalmologist.

Tonometry

Intraocular pressure for both eyes will be measured using a non-contact tonometry. Tonometry should be performed prior to prior to pupil dilation. Tonometry will be performed by a masked Examining Ophthalmologist.

Perimetry

Perimetry will be performed by a masked Examining Ophthalmologist for the exact, quantitative assessment of the extent of space visible in a patient’s periphery. Perimetry will be obtained prior to pupil dilation.

Slit-lamp biomicroscopy

A standard ophthalmic examination using slit lamp biomicroscopy will be performed in both eyes by the unmasked Primary Treating Ophthalmologist. The Anterior chamber cell count will be measured during the examination according to the SUN criteria (see Appendix II), which should be performed prior to pupil dilation.

Ophthalmoscopy

Ophthalmoscopy will be performed by the Primary Treating Ophthalmologist under the dilation of the pupil to determine both vitreous haze grading and the absence/presence of active chorioretinal and/or retinal vascular lesions. Grading of vitreous haze will be based on the publication from the National Eye Institute (NEI) which has also been adapted by the SUN working group (see Appendix III).

Fundus photography

Fundus photography will be performed by a masked Examining Ophthalmologist to obtain documented evidence of the absence/presence of active chorioretinal and/or retinal vascular lesions.

Optical coherence tomography (OCT)

Optical coherence tomography will be performed by a masked Examining Ophthalmologist to determine the central retinal thickness and the presence of macular alternations.

Fundus fluorescence angiography (FFA)

Fundus fluorescence angiography will be performed by a masked Examining Ophthalmologist to determine the absence/presence of active chorioretinal and/or retinal vascular lesions. OCT will be performed by masked Examining Ophthalmologists.

B ultrasonography and ultrasound biomicroscope (UBM)

B ultrasonography and ultrasound biomicroscope may be performed at the

Investigators' discretion according to the clinical needs and will not be mandatory during the trial. These two auxiliary examinations will be carried out by masked Examining Ophthalmologists who will write the examination reports.

Tuberculosis screening

Both PPD test and T-SPOT.TB test are eligible for the TB screening. Those subjects with a positive PPD or T-SPOT.TB need to additionally undertake the chest computed tomography to exclude active tuberculosis.

Human immune deficiency virus (HIV) screening

HIV tests will be performed based on an ELISA (enzyme-linked immunosorbent assay) technique, which seeks to detect both antigens (p24) and antibodies.

Hepatitis B, Hepatitis C and syphilis screening

Syphilis serological tests, hepatitis B surface antigen and hepatitis C antibody tests, (and if necessary, HBV-DNA PCR) will be performed to exclude active hepatitis B, hepatitis C and syphilis.

Clinical laboratory tests

Laboratory evaluations will be performed including Routine blood test (Hematocrit, Hemoglobin, Red Blood Cell count, White Blood Cell count, Neutrophils, Lymphocytes, Monocytes, Basophils, Eosinophils, Platelet count), Biochemical test (Blood Urea Nitrogen [BUN], Creatinine, Uric acid, Serum albumin, Total protein, Total bilirubin, Serum glutamic-pyruvic-transaminase [SGPT/ALT], Serum glutamic-oxaloacetic-transaminase [SGOT/AST], Alkaline phosphatase, Sodium Potassium, Calcium, Inorganic phosphorus), C-reactive protein and erythrocyte sedimentation rate. The urine pregnancy test will be performed if subjects have a childbearing potential. Specimens for Clinical laboratory tests will be collected according to local hospital or lab requirement and will be destroyed after testing.

5.7 Study Schedule Overview

	Screening	Baseline	Week 2	Week 9 (Month 2)	Week 17 (Month 4)	Week 26 (Month 6)	Unscheduled visit
Visit No.	0	1	2	4	5	6	
Visit window (day)	-10	0	±7	±14	±14	±21	
Informed consent	×						
Inclusion/exclusion criteria	×						
Randomization		×[a]					
General information and medical history	×						
Pregnancy test in women of child bearing potential	×						
Questionnaires		×[a]				×	
Vital signs/weight/height	×		×	×	×	×	×
Physical examination	×		×	×	×	×	×
Visual acuity testing	×		×[d]	×	×	×	×[d]
Tonometry	×		×	×	×	×	×
Perimetry	×			×	×	×	
Slit-lamp biomicroscopy	×	×[b]	×	×	×	×	×
Ophthalmoscopy	×	×[b]	×	×	×	×	×
Fundus photography	×			×	×	×	
Optical coherence tomography	×			×	×	×	
Fundus fluorescence angiography	×			×	×	×	
B ultrasonography	×[e]			×[e]	×[e]	×[e]	
Ultrasound biomicroscope	×[e]			×[e]	×[e]	×[e]	
Tuberculosis screening	×						
HIV screening	×						
Hepatitis B screening	×						
Hepatitis C screening	×						
Syphilis testing	×						
Routine blood test	×		×	×	×	×	×[e]
Biochemical test	×		×	×	×	×	×[e]
C-reactive protein		×[a]		×	×	×	
Erythrocyte sedimentation rate		×[a]		×	×	×	
Treatment and record		×	×	×	×	×	×
Protocol deviation evaluation			×	×	×	×	×

	Screening	Baseline	Week 2	Week 9 (Month 2)	Week 17 (Month 4)	Week 26 (Month 6)	Unscheduled visit
Visit No.	0	1	2	4	5	6	
Visit window (day)	-10	0	±7	±14	±14	±21	
Adverse event evaluation		×[c]	×	×	×	×	×

[a] Should be performed before the initiation of treatment.
[b] Will not be repeated if the Screening and Baseline are on the same day.
[c] Will be assessed after the initiation of treatment.
[d] Will be assessed with a Logarithmic visual acuity chart only.
[e] Will be at the Investigators' discretion according to the clinical needs.

6 Treatment Plan

6.1 Stratification

Before randomization, the phase of VKH disease will be determined. The subject will be diagnosed with early or late phase VKH disease according to the Diagnostic Criteria (see Appendix I).

Subjects will be randomized in a 1:1 ratio to receive either conventional or biological treatment regimens using the phase of VKH disease as stratification factor to ensure the balance of this factor between the two arms.

6.2 Corticosteroids

Systemic corticosteroids will be implemented for both arms in the same dose and duration.

The initial dose of corticosteroids (oral prednisone) for early and late phase VKH disease is summarized as follows:

Initial dose of oral prednisone

Phase	Dose [a]
Early phase VKH disease	0.6-0.8 mg/kg/day (or 30-40 mg/day)
Late phase VKH disease	0.4-0.6 mg/kg/day (or 20-30 mg/day)

[a] An equipotent dose of an alternative oral corticosteroid medication can be used if a specific contraindication to prednisone exists.

The initial dose of corticosteroids will be used for 1 or 2 weeks and then gradually tapered. The daily dose of corticosteroids will be decreased by 5 mg every 1-2 weeks, with a goal of tapering and holding at 15 mg until the end of 6 months.

6.3 Conventional Treatment Regimen

In combination with corticosteroids, regardless of early or late phase VKH disease,

subjects will be orally treated with cyclosporine at a dose of 2-4 mg/kg/day (or 100-200 mg/day) throughout the trial.

If a specific contraindication to cyclosporine exists, subjects will be orally treated with chlorambucil at a dose of 0.05-0.1 mg/kg/day throughout the trial.

If subjects are diagnosed with early-phase VKH disease but without exudative retinal detachment, Investigator may consider using the corticosteroid treatment alone rather than in combination with cyclosporine. But in this case, it will not be mandatory to use only corticosteroids for the treatment of disease. The Investigator has the option to initiate the cyclosporine at any time throughout the trial, depending on patient's condition and Investigator's judgment.

Step-up Treatment

For subjects initially randomized to the conventional treatment arm, a step-up treatment may be considered if patients have an inadequate response to the treatment meeting one of the following criteria in at least one eye:

a. Two-step increase in anterior chamber cells relative to the Baseline throughout the study period (See SUN criteria in Appendix II). This is represented by a change of Grade 0 to Grade 2+; or Grade 0.5+ to Grade 3+.

b. Two-step increase in vitreous haze relative to the Baseline throughout the study period (See NEI/SUN criteria in Appendix III). This is represented by a change of Grade 0 to Grade 2+; or Grade 0.5+ to Grade 3+.

c. New active, inflammatory chorioretinal and/or inflammatory retinal vascular lesions relative to the Baseline.

The Investigator has the option to determine which step-up treatment may be implemented, including but not limited to adding another immunosuppressive drug (chlorambucil, oral, 0.05-0.1 mg/kg/day) or transition to biological treatment (adalimumab, subcutaneous injection, 40 mg once every 2 weeks).

6.4 Biological Treatment Regimen

Adalimumab

In combination with corticosteroids, all subjects randomized to the biological treatment arm will receive subcutaneous injection of adalimumab at a dose of 40 mg once every 2 weeks throughout the trial.

6.5 Other Adjunctive Treatments

Adjunctive treatments described below can be used for subjects in both arms, and all these adjunctive treatments should be recorded on the case report form (CRF) at each visit, if implemented. Subjects can use different doses of topical corticosteroid drops into the study. To prevent side effect (such as increased intraocular pressure), topical corticosteroid drops cannot be used for a long term and should be gradually tapered to completely discontinue the topical use of corticosteroids, if the active anterior inflammation is controlled. Topical adjunctive medications such as cycloplegic agent will be permitted during the study, to be used according to best medical judgment. Intraocular pressure lowering medications, including but not limited to eye drops, drugs and even surgery, will be performed for patients with intraocular pressure elevation and glaucoma secondary to uveitis itself, its complications or induced by corticosteroid, if necessary. Unless there is a contraindication, the occurrence and progression of cataract will be evaluated and properly treated by surgery, YAG laser capsulotomy or surgical capsulotomy during the study period. Supplementation of vitamin D, calcium, or potassium can be implemented in the study to minimize the incidence of adverse events caused by corticosteroids. Patients developing hypertension or diabetes from systemically-administered drugs will be referred to their primary care physician for appropriate management. All adverse events will be noted and reported through the study.

6.6 Prohibited Therapy

The introductions of following medications for VKH disease are prohibited during

this clinical trial:

- All other biologic therapies.
- All other immunosuppressive therapies (not including step-up treatment, cyclosporine and chlorambucil).
- Systemic or topical non-steroidal anti-inflammatory drugs.
- Live attenuated vaccines.
- Anti-retroviral therapy.
- Periocular or intravitreal corticosteroid injection.
- Glucocorticosteroid implant.
- Anti-VEGF intravitreal injection.

7 Outcome Assessments

7.1 Primary Outcome

The primary outcome will be the change in BCVA letter score measured using an ETDRS chart from Baseline to the end of 26 weeks.

7.2 Secondary Outcomes

a. Proportion achieving a ≥ 15 ETDRS letter improvement (appreciable visual gain) at the end of 26 weeks.

b. Change in visual field indices (visual field index, mean deviation and pattern standard deviation) from Baseline to the end of 26 weeks.

c. Proportion of participants achieving an inactive uveitis (an anterior chamber cell grade of 0.5+ or less, a vitreous haze grade of 0.5+ or less, and no active inflammatory choroidal or retinal lesions) in both eyes at the end of 26 weeks.

d. Percent change in the presence of retinal detachment from Baseline to the end of 26 weeks.

e. Changes in OCT anatomical features (central macular thickness) from the Baseline to the end of 26 weeks.

f. Proportion receiving a step-up treatment in the conventional treatment group throughout the study period.

g. Change in EQ-5D scores from the Baseline to the end of 26 weeks.

h. Change in VFQ-25 composite score from the Baseline to the end of 26 weeks.

7.3 Safety Outcomes

Safety and tolerability as defined by the following:

- Type, frequency, severity, and relationship of the adverse events (AEs) to study

drugs.

- Number of subjects who prematurely discontinue study drugs due to any AE.
- Frequency of clinically significant changes in vital signs, and/or laboratory findings.

7.4 Pre-specified Subgroup Analyses

To determine differences between arms in mean change in BCVA ETDRS letter score at 26 weeks across subgroup variable defined by early or late phase VKH disease.

7.5 Cost Effectiveness

The incremental cost-effectiveness ratio calculation is the difference in costs divided by the difference in the number of quality adjusted life years (QALYs) that result from the two treatments over the time period being described:

[Cost(Conventional)-Cost(Biological)]/[QALYs(Conventional)-QALYs(Biological)]

When we use measures other than QALYs, we will also calculate the dollars spent per visual acuity outcome improvement (taking the methodological limitations into account in interpreting this result).

8 Adverse Events

8.1 Adverse Event Reporting

An adverse event (AE) is defined as any unfavorable medical occurrence in a subject who has ever received study medication, regardless of a causal relationship with this treatment. Any worsening of a pre-existing condition or illness should be considered an adverse event. Worsening in severity of a reported adverse event should also be reported as a new adverse event. An elective surgery/procedure scheduled to occur during a study will not be considered an adverse event if the surgery/procedure is being performed for a pre-existing condition and the surgery/procedure has been pre-planned prior to study entry. However, if the pre-existing condition deteriorates unexpectedly during the study (e.g., surgery performed earlier than planned), then, the deterioration of the condition for which the elective surgery/procedure is being done will be considered an adverse event. For adverse events to be considered intermittent, the events must be of similar nature and severity.

The independent Investigator will monitor each subject for clinical and laboratory evidence of adverse events on a routine basis throughout the study. The Investigator will assess and record any adverse event in detail including the date of onset, event diagnosis (if known) or sign/symptom, severity, time course, duration and outcome, relationship of the adverse event to study drug and any actions taken. All these information on non-serious and serious adverse events will be recorded on the CRF at each study visit.

8.2 Serious Adverse Events

Serious adverse events (SAE) should be reported to Chief Investigator and Ethics Committee within 24 hours of the Investigator being made aware of the SAE. Serious adverse events include any medical occurrence that results in the following outcomes:

Death.

Life-threatening experience.

Non-elective surgery or hospitalization for any reason.

Congenital anomaly or birth defect.

Persistent or significant disability or incapacity.

Any other important medical events, not immediately life-threatening or resulting in death or hospitalization but requiring medical or surgical intervention to prevent serious outcome.

8.3 Severity Assessments

The severity of adverse event can be assessed according to the following criteria:

Mild: The adverse event does not interfere with the volunteer's daily routine, and does not require intervention; it causes slight discomfort.

Moderate: The adverse event interferes with some aspects of the volunteer's routine, or requires intervention, but is not damaging to health; it causes moderate discomfort.

Severe: The adverse event results in alteration, discomfort or disability which is clearly damaging to health.

8.4 Causality Assessments

The Investigator should assess the relationship of the adverse event with the use of study medication by reporting according to the three-level classification of "associated", "not associated", and "inability to determine". Causality assessments will be required for study drugs as well as other adjunctive treatments.

8.5 Uveitis-Related Events

The following events are known complications related to the condition being treated

and will be classified as uveitis-related events. These events will be analyzed separately from other adverse events in the final study report. Notably, the following events may not be a complete list of all potential uveitis-related events. The Investigators must determine if a specific event is uveitis-related.

Loss of transparency of the cornea.

Band keratopathy.

Synechiae.

Cataracts.

Glaucoma/increased intraocular pressure.

Vitreous hemorrhage.

Macular edema.

Retinal detachment.

Epiretinal membrane.

Vitreo-macular traction.

Retinal ischemia.

Vision loss.

Hypotony.

8.6 Pregnancy

Subjects who become pregnant during the study period must be discontinued. Pregnancy in a study subject is not considered as an adverse event. However, the medical outcome of an elective or spontaneous abortion, stillbirth or congenital anomaly is considered as a serious adverse event.

8.7 Management

For the purpose of medical management, all adverse events and laboratory abnormalities that occur during the study must be evaluated by the Investigator. The occurrence of adverse events does not necessarily mean the time for the subject to exit the study. But, a serious adverse event will absolutely result in the discontinuation of the study immediately. For those developing non-serious adverse events, reducing the medication dose, administration of other adjunctive treatments or even temporarily stopping the medication may be used. Study medication may be restarted, even allowed in a modified dose, once the Investigator determines that the abnormalities have been successfully dealt with. Otherwise, at this point, patients will be recommended to withdraw from the study and be treated according to the best medical judgment of their clinician.

9 Data Collection and Management

9.1 Confidentiality

Participants will be identified via a unique ID. Identifiable information will not be stored in the eCRF and will not leave the site. Any participant contact information will be stored within the site on password protected computers or within secured locations with limited access. All study data and site files will be kept at site in a secure location with restricted access.

9.2 Source and Data Collection

Written informed consent will be obtained prior to screening and any other study specific procedures are performed. The study will employ an eCRF, whereby data will be managed via this system. Source data worksheets will be used for each patient and data will be entered onto the eCRF database. Source data worksheets will be reconciled at the end of the trial with the patients' medical notes in the study site. During the trial, critical clinical information will be written in the medical notes to ensure informed medical decisions can be made in the absence of the study team. Trial related clinical letters will be copied to the medical notes during the trial. The Principal Investigator will provide a signature for CRF once all queries are resolved and immediately prior to database lock.

It will be the responsibility of the Principal Investigator and his team to ensure the accuracy of all data entered in the worksheets and the eCRF are in accordance with Good Clinical Practice. The Principal Investigator will be responsible for ensuring that source data worksheets are filed in a suitably secure location to ensure source data verification can be undertaken throughout the study.

9.3 Quality Assurance

The study will incorporate a range of data management quality assurance functions as

the trial progresses. The Trial Data Manager will provide study training, ongoing study support and will conduct regular monitoring, checking source data for transcription errors. Any necessary alterations to entered data will be date and time stamped within the eCRF. Regular monitoring of study conduct and data collected will be performed to ensure the study is conducted in accordance with GCP.

9.4 Database Lock

Prior to database lock, the Trial Manager will review any outstanding warnings on the eCRF and resolve or close these as appropriate before database lock. Local study personnel should resolve any queries that arise promptly. Once all queries have been resolved no further changes will be made to the database unless specifically requested by the Study Office in response to the Statistician's data checks. The study Principal Investigator will review all the data and provide electronic signature to verify that all the data are complete and correct. At this point, all data will be formally locked for analysis.

9.5 Archiving

Principal Investigators will be responsible for securely archiving local data generated, essential documents and source data in accordance with local requirements, but for at least 5 years from the end of the study.

10 Statistical Considerations

10.1 Study Hypothesis

There is one formal comparison of interest on primary outcome:

Conventional Treatment vs. Biological Treatment

Conventional Treatment is hypothesized to be not inferior to biological Treatment, whereby the mean of change in best corrected ETDRS visual acuity letter score of conventional treatment arm is not worse than that of biological treatment by a margin of seven letters. This margin represents the minimal clinically important difference, based on clinical trial results of pivotal trials of treatments in uveitis and wet macular degeneration.[9]

The null hypothesis, that conventional treatment is inferior to biological Treatment, will be rejected if the estimated 95% confidence interval for the difference in treatment means lies wholly above the seven letter margin.

10.2 Sample Size Calculation

Assuming the standard deviation of the change in best corrected ETDRS visual acuity letter score at 10 for both conventional treatment arm and biological treatment arm, sample size of 44 subjects per arm would provide 90% power to show non-inferiority at a margin of difference less than 7 between these two arms based on a one-side significance level of 0.025. Expecting 20% dropout rate for those subjects lost during the course of the study and for whom no response data will be collected, the final dropout-inflated enrollment would be 55 subjects per group and 110 in total.

10.3 Data Analyses

Analyses will be on both an Intention-to-treat (ITT) basis and a Per Protocol (PP) basis. Safety analyses will be conducted in the safety set which includes all subjects

recruited and received at least one dose of study medication. A detailed statistical analysis plan will be prepared and finalized prior to database lock.

10.4 Interim Analysis

This study does not plan to perform interim analysis.

11 Ethical Considerations

This clinical trial will be conducted in compliance with the approved protocol, the Helsinki Declaration, the Good Clinical Practice (GCP) guidelines issued by the CFDA, and corresponding regulations.

Before the commencement of this experiment, the approval of the ethics committee must be obtained.

The Investigator is expected to take any immediate action required for the safety of any patient included in this study, even if this action represents a deviation from the protocol.

During the clinical study, any changes made to this trial protocol should be reported to the Ethics Committee and placed on record.

Subjects must provide informed consent to participate in the trial.

If the subject and his or her legal representative are illiterate, the informed consent process shall be attended by a witness, who shall sign the informed consent form after oral consent by the subject or his or her legitimate representative.

A copy of the informed consent form and the contact information for the researcher and the ethics committee will be provided to the patient.

The study site must be equipped with the necessary medical rescue equipment and first aid drugs for emergency issues.

REFERENCES

1. Du L, Kijlstra A, Yang P. Vogt-Koyanagi-Harada disease: Novel insights into pathophysiology, diagnosis and treatment. Prog Retin Eye Res 2016;52:84-111.

2. Zhong Z, Su G, Kijlstra A, Yang P. Activation of the interleukin-23/interleukin-17 signalling pathway in autoinflammatory and autoimmune uveitis. Prog Retin Eye Res 2020;16:100866.

3. Yang P, Ren Y, Li B, Fang W, Meng Q, Kijlstra A. Clinical characteristics of Vogt-Koyanagi-Harada syndrome in Chinese patients. Ophthalmology 2007;114:606-14.

4. Dick AD, Rosenbaum JT, Al-Dhibi HA, et al. Guidance on Noncorticosteroid Systemic Immunomodulatory Therapy in Noninfectious Uveitis: Fundamentals Of Care for UveitiS (FOCUS) Initiative. Ophthalmology 2018;125:757-73.

5. Yang P, Ye Z, Du L, et al. Novel treatment regimen of Vogt-Koyanagi-Harada disease with a reduced dose of corticosteroids combined with immunosuppressive agents. Curr Eye Res 2018;43:254-61.

6. Jabs DA, Rosenbaum JT, Foster CS, et al. Guidelines for the use of immunosuppressive drugs in patients with ocular inflammatory disorders: recommendations of an expert panel. Am J Ophthalmol 2000;130:492-513.

7. Jaffe G, Dick A, Brézin A, et al. Adalimumab in patients with active noninfectious uveitis. N Engl J Med 2016;375.

8. Nguyen QD, Merrill PT, Jaffe GJ, et al. Adalimumab for prevention of uveitic flare in patients with inactive non-infectious uveitis controlled by corticosteroids (VISUAL II): a multicentre, double-masked, randomised, placebo-controlled phase 3 trial. Lancet 2016;388:1183-92.

9. Writing Committee for the Multicenter Uveitis Steroid Treatment Trial, Follow-up Study Research Group, Kempen JH, et al. Association Between Long-Lasting Intravitreous Fluocinolone Acetonide Implant vs Systemic Anti-inflammatory Therapy and Visual Acuity at 7 Years Among Patients With Intermediate, Posterior, or Panuveitis. JAMA 2017;317:1993-2005.

10. Yang P, Zhong Y, Du L, et al. Development and evaluation of diagnostic criteria for Vogt-Koyanagi-Harada disease. JAMA Ophthalmol 2018;136:1025-31.

Appendix I

Diagnostic Criteria for Vogt-Koyanagi-Harada (VKH) Disease

A. No history of penetrating ocular trauma or intraocular surgery preceding the initial onset of uveitis

B. Bilateral ocular involvement (time interval between the 2 eyes should be 2 wk)

C. No evidence of infectious uveitis or accompanying systemic rheumatic diseases or evidence suggestive of other ocular disease entities[a]

D. Early-phase VKH disease:

1. Signs of diffuse choroiditis and exudative retinal detachment
2. Serous retinal detachment on OCT or B-scan ultrasonography
3. Choroidal thickening on EDI-OCT[b]
4. Early punctate staining and late subretinal dye pooling on FFA
5. Hyperfluorescence of the optic disc on FFA

Definite diagnosis:

Variant 1: In patients presenting with A + B + C + D(1)

Variant 2: In patients without clinically visible exudative retinal detachment, ie, A + B + C + D(2) + D(3) or A + B + C + D(4)

Variant 3: In patients already treated with systemic corticosteroids or combined with other immunosuppressive agents, a history of typical appearances of variant 1 or 2, and A + B + C + D(5)

E. Late-phase VKH disease

1. Signs of definite sunset glow fundus or retinal pigment epithelium clumping/migration
2. Signs of bilateral recurrent granulomatous anterior uveitis
3. Signs of Dalen-Fuchs nodules or multifocal chorioretinal atrophy
4. Window defects/moth-eaten fluorescence on FFA
5. Previous history of characteristic findings corresponding to diagnosis of early-phase VKH disease

Definite diagnosis:<br>Variant 1: In patients presenting with A + B + C + E(1) + E(2)<br>Variant 2: In patients without sunset glow fundus or visible pigment alternations due to early and appropriate treatment, ie, A + B + C + E(2) + E(3) or A + B + C + E(2) + E(4)<br>Variant 3: In patients with significant media opacity, ie,<br>A + B + C + E(2) + E(5)

Abbreviations: EDI, enhanced depth imaging; FFA, fluorescence fundus angiography; OCT, optical coherence tomography.

[a] This criterion includes (1) no history nor clinical evidence to show ocular tuberculosis, syphilis, or ocular toxoplasmosis; (2) no underlying systemic rheumatic disease that could explain the form of uveitis these patients have; and (3) no history or clinical evidence to suggest the possibility of a specific entity, for instance intraocular tumors, toxic uveitis, Fuchs syndrome, or Posner-Schlossman syndrome.

[b] Ultrasonography can be used to detect the choroidal thickening and therefore may serve as an alternative in the examination where the EDI-OCT is not available, although it is less precise.

From Yang P et al. Development and Evaluation of Diagnostic Criteria for Vogt-Koyanagi-Harada Disease. JAMA Ophthalmol. 2018;136(9):1025-1031.

Appendix II

SUN Working Group Grading System for Anterior Chamber Cells

Grade	Number of cells in field [a]
0	<1
0.5+	1-5
1+	6-15
2+	16-25
3+	26-50
4+	>50

[a] Field size is a 1mm by 1mm slit lamp beam. Grading should be done with the highest magnification and illumination and conducted in a completely dark room, prior to dilation.

From Jabs DA et al. Standardization of uveitis nomenclature for reporting clinical data. Results of the First International Workshop. Am J Ophthalmol 2005;140(3):509-16.

Appendix III

National Eye Institute (NEI)/SUN Criteria for Grading Vitreous Haze

Grade	Description [a]
0	No evident vitreal haze
0.5+	Slight blurring of the optic disc margin because of the haze; normal striations and reflex of the nerve fiber layer cannot be visualized
1+	Permits a better definition of both the optic nerve head and the retinal vessels (compared to higher grades)
2+	Permits better visualization of the retinal vessels (compared to higher grades)
3+	Permits the observer to see the optic nerve head, but the borders are quite blurry
4+	Optic nerve head is obscured

[a] Grading should be conducted in a completely dark room.

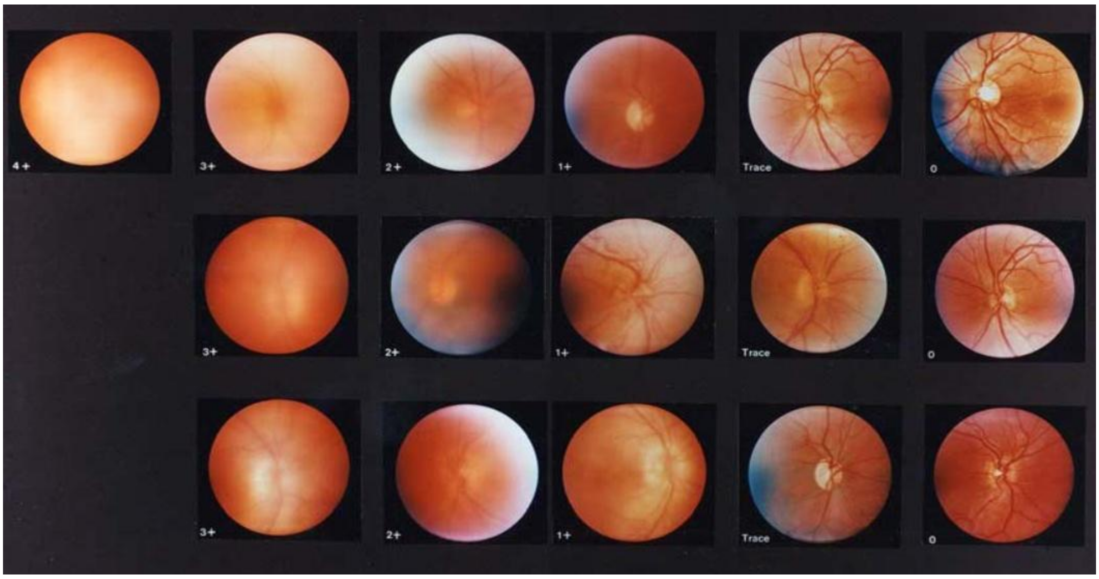

From Nussenblatt et al. Standardization of vitreal inflammatory activity in intermediate and posterior uveitis. Ophthalmology 1985;92:467-471.

Conventional versus Biological Treatment for Vogt-Koyanagi-Harada Disease

A Randomized, Open-label, Blinded-endpoint,

Non-inferiority Trial

Study Protocol

Version Number: 1.0

The First Affiliated Hospital of Chongqing Medical University,

Chongqing Key Laboratory of Ophthalmology and Chongqing Eye Institute,

Chongqing, China

Protocol Title: Conventional versus Biological Treatment for Vogt-Koyanagi-Harada Disease: A Randomized, Open-label, Blinded-endpoint, Non-inferiority Trial

Trial Code/Short Title: VKH-I

Version Number: 1.0

Protocol Date: January 1st, 2021

Site: Single center (The First Affiliated Hospital of Chongqing Medical University)

Grant: Chongqing Key Laboratory of Ophthalmology (CSTC, 2008CA5003)

Back-up 24 Hour Global Emergency Contact Call: [redacted]

The back-up 24 hour global emergency contact call should only be used if you are not able to reach the Clinical Research Physician(s) or Study Coordinator(s) for emergency calls.

CONTENTS

CONTENTS 3

ABBREVIATIONS 6

1 Introduction 7

1.1 Background 7

1.2 Benefits and Risks 7

2 Objectives 9

2.1 Trial Objectives 9

2.2 Primary Objective 9

2.3 Secondary Objectives 9

2.4 Safety Objectives 9

3 Study Design 10

3.1 Overall Design 10

3.2 Study Schematic 10

3.3 Study Timeline 11

4 Eligibility Criteria 12

4.1 Inclusion Criteria 12

4.2 Exclusion Criteria 13

4.3 Re-screening of Patients 15

5 Study Procedures 16

5.1 Informed Consent Procedure 16

5.2 Eligibility Evaluation 16

5.3 Randomization Assignment and Masking 16

5.4 Baseline and Subsequent Assessments 17

5.5 Withdrawn and Lost to Follow-up 18

5.6 Study Assessments and Methods 19

5.7 Study Schedule Overview 23

6 Treatment Plan 25

6.1 Stratification 25
6.2 Corticosteroids 25
6.3 Conventional Treatment Regimen 25
6.4 Biological Treatment Regimen 26
6.5 Other Adjunctive Treatments 27
6.6 Prohibited Therapy 27
7 Outcome Assessments 29
7.1 Primary Outcome 29
7.2 Secondary Outcomes 29
7.3 Safety Outcomes 29
7.4 Pre-specified Subgroup Analyses 30
7.5 Cost Effectiveness 30
8 Adverse Events 31
8.1 Adverse Event Reporting 31
8.2 Serious Adverse Events 31
8.3 Severity Assessments 32
8.4 Causality Assessments 32
8.5 Uveitis-Related Events 32
8.6 Pregnancy 33
8.7 Management 34
9 Data Collection and Management 35
9.1 Confidentiality 35
9.2 Source and Data Collection 35
9.3 Quality Assurance 35
9.4 Database Lock 36
9.5 Archiving 36
10 Statistical Considerations 37
10.1 Study Hypothesis 37

10.2 Sample Size Calculation 37

10.3 Data Analyses 37

10.4 Interim Analysis 38

11 Ethical Considerations 39

REFERENCES 40

Appendix I 41

Appendix II 43

Appendix III 44

ABBREVIATIONS

AE	Adverse event
ALT	Alanine aminotransferase
AST	Aspartate aminotransferase
BCVA	Best corrected visual acuity
BUN	Blood Urea Nitrogen
CFDA	China Food and Drug Administration
CRF	Case report form
FFA	Fluorescein fundus angiography
EQ-5D	EuroQol-5D Questionnaire
ETDRS	Early treatment diabetic retinopathy study
MCID	Minimal clinically important difference
NEI	National Eye Institute
NYHA	New York Heart Association
OCT	Optical coherence tomography
PRO(s)	Patient reported outcome(s)
QALY(s)	Quality adjusted life year(s)
SUN	Standardization of Uveitis Nomenclature Working Group
TNF	Tumor necrosis factor
UBM	Ultrasound biomicroscope
VFQ-25	Visual functioning questionnaire 25
VKH	Vogt-Koyanagi-Harada

1 Introduction

1.1 Background

Vogt-Koyanagi-Harada (VKH) disease is an immune-mediated disorder characterized by bilateral uveitis frequently associated with neurological (meningeal), auditory, and integumentary manifestations.[1] Although the etiology and pathogenesis of VKH disease need to be further elucidated, it is widely accepted that the clinical manifestations are caused by an autoimmune response directed against melanin associated antigens in the target organs, i.e. the eye, inner ear, meninges and skin.[2] The most frequent complaint in patients with VKH disease is a rapid decrease in vision in one or both eyes following a transiently blurred vision.[3]

Currently, the treatment for VKH disease has not been extensively evaluated in prospective clinical trials and the treatment is generally empirical in clinical practice. Glucocorticosteroids and conventional immunosuppressive agents such as cyclosporine are still the most often prescribed drugs for VKH disease.[4-6] Adalimumab, a recombinant human monoclonal antibody that specifically binds and inhibits a proinflammatory cytokine, tumor necrosis factor (TNF), has been demonstrated the efficacy for noninfectious uveitis and yielded promising results in the treatment of uveitis refractory to conventional therapy.[7,8] Recently, it has been recognized that VKH disease is largely heterogeneous and treatment for the disease may be individualized.[3,4] It remains unclear whether an individualized, conventional drug based treatment strategy is as effective as the biological treatment for VKH disease.

Therefore, we aim to initiate a clinical trial to compare the clinical and cost effectiveness between the conventional and biological treatment strategies for VKH disease.

1.2 Benefits and Risks

This trial will determine if there are any differences in clinical and cost effectiveness, safety profile and other outcomes between conventional drugs and biological drugs in a randomized trial, of which results will guide clinical decision-making in the treatment for VKH disease.

This trial may incur some risks to subjects, mostly related to the common side effects for study drugs, such infection, allergy, injection-site reaction. Nevertheless, the study drugs had been evaluated for the treatment of other diseases in phase 3 clinical trials,[7,8] which suggested that these study drugs were generally safe.

2 Objectives

2.1 Trial Objectives

The objective is to compare the clinical and cost effectiveness of an individualized treatment strategy based on conventional immunosuppressive drugs with that of a standardized treatment strategy based on the biological drug, adalimumab in Vogt-Koyanagi-Harada disease. We aim to determine if the conventional treatment is as effective as the biological treatment in treating visual loss in Vogt-Koyanagi-Harada disease and whether they have an equivalent side effect profile.

2.2 Primary Objective

To determine whether the conventional treatment is non-inferior to the biological treatment in improving the best corrected visual acuity (BCVA) at 26 weeks among patients with Vogt-Koyanagi-Harada disease.

2.3 Secondary Objectives

To compare efficacy, clinical outcomes, cost effectiveness and patient reported outcomes (PROs) between the conventional and biological treatment groups in Vogt-Koyanagi-Harada disease.

2.4 Safety Objectives

To evaluate the safety and tolerability of the conventional versus biological treatment for Vogt-Koyanagi-Harada disease.

3 Study Design

3.1 Overall Design

This is an investigator-initiated, randomized, open-label, blinded-endpoint, non-inferiority clinical trial to compare the clinical and cost effectiveness of conventional and biological treatment strategies/regimens for subjects with Vogt-Koyanagi-Harada disease and who have an active inflammation within the last 90 days in any eye and/or require chronic oral prednisone ≥ 20 mg/day for the treatment for intraocular inflammation.

Approximately 110 eligible subjects will be randomized 1:1 (55 subjects per group) to receive one of two treatment strategy arms using the disease phase (early or late phase VKH) as the stratification factor. In the conventional treatment arm, subjects will receive an individualized regimen based on prednisone primarily combined with cyclosporine. In the biological treatment arm, subjects will receive a standardized regimen of prednisone and adalimumab throughout the trial. All participants will be followed up for 26 weeks since randomization.

The primary outcome will be the difference in mean Early Treatment Diabetic Retinopathy Study (ETDRS) BCVA letter score at 26 weeks. The minimal clinically important difference (MCID) for change in letters read is 7 letters, based on clinical trial results of pivotal trials of treatments in uveitis and wet macular degeneration.[9] This trial aims to test whether the mean change in ETDRS BCVA letters from randomization to Week 26 is not different by more than a margin of 7 letters.

3.2 Study Schematic

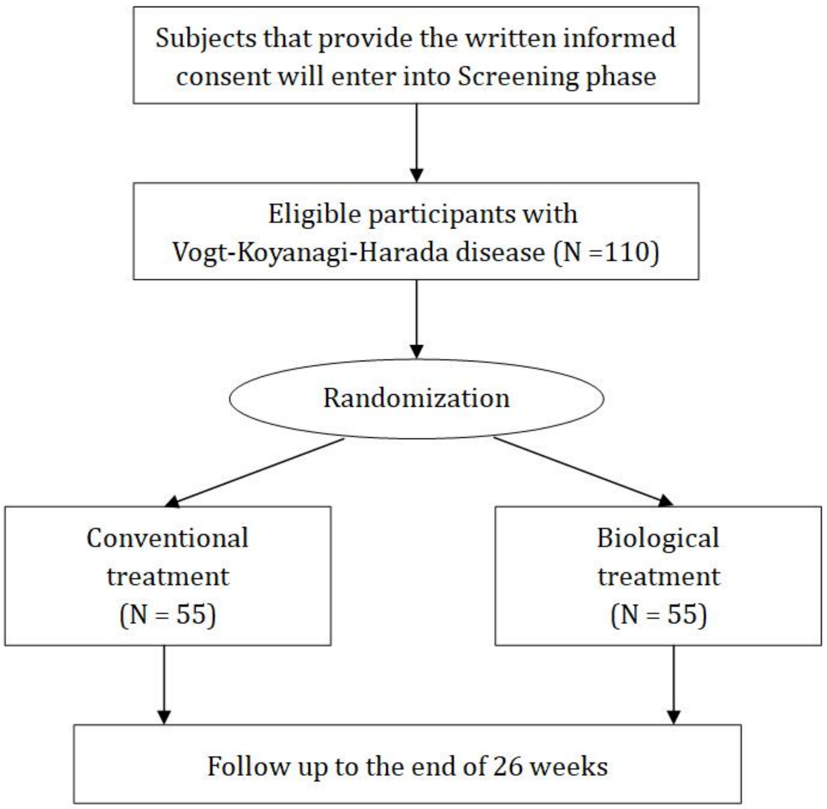

3.3 Study Timeline

Expected time for trial start: February 2021

Expected completion time of enrollment: September 2021

Expected time for end of trial: March 2022

The end of trial refers to the date of the last visit of the last subject to complete the study, or the date of receipt of the last data point from the last subject that is required for primary, secondary and/or exploratory analysis, as pre-specified in the protocol and/or the Statistical Analysis Plan, whichever is the later date.

Expected completion time of primary analysis: August 2022

4 Eligibility Criteria

4.1 Inclusion Criteria

A subject will be eligible for study participation if she/he meets all of the following inclusion criteria:

a. Subjects of either sex aged ≥ 18 years at the time of signing the informed consent document.

b. Diagnosed with Vogt-Koyanagi-Harada disease. See Diagnostic Criteria for Vogt-Koyanagi-Harada disease in Appendix I. [10]

c. An active disease status:

   Requiring chronic oral prednisone (≥ 20 mg or equivalent) for the treatment for intraocular inflammation;

   And/OR

   Having an active inflammation or having documented history of experiencing an active inflammation within the last 90 days as defined by the presence of at least one of the following parameters in either eye:

   (1) ≥ 2+ anterior chamber cells (See SUN criteria in Appendix II);

   (2) ≥ 2+ vitreous haze (See NEI/SUN criteria in Appendix III);

   (3) Presence of active inflammatory choroidal or retinal lesions detected by optical coherence tomography (OCT) or fluorescein fundus angiography (FFA).

d. Media clarity, pupillary dilatation and subject cooperation judged by the study Investigator for proper viewing of the posterior segment in at least one eye.

e. Able and willing to consistently use effective contraceptive methods in order to successfully prevent pregnancy.

f. Able and willing to self-administer subcutaneous injections or have a qualified condition available to administer subcutaneous injections.

g. Able to adhere to the study visit schedule and other protocol requirements.

4.2 Exclusion Criteria

The presence of any of the following will exclude a subject from the study enrollment:

a. Visual acuity of hand motions or worse in the better-seeing eye.

b. Previous exposure to anti-TNF therapy.

c. Using > 1 systemic immunosuppressive therapy (not including corticosteroids) within the last 28 days.

d. Uncontrolled intraocular pressure with ≥ 2 glaucoma medications or evidence of optic nerve injury.

e. Chronic hypotony (IOP < 5 mm Hg for > 3 months) in both eyes.

f. Received intraocular or periocular corticosteroids in the past 3 months.

g. Received Ozurdex® (dexamethasone implant) in the past 6 months.

h. Received Retisert® (glucocorticosteroid implant) within 3 years prior to the Screening.

i. Proliferative or severe non-proliferative diabetic retinopathy or clinically significant macular edema due to diabetic retinopathy.

j. Neovascular/wet age-related macular degeneration.

k. History of moderate to severe congestive heart failure (NYHA class III or IV) or recent cerebrovascular accident.

l. Current or history of demyelinating disease such as multiple sclerosis.

m. Malignancy or history of malignancy.

n. White blood cells $< 3 \times 10^9$/L at the Screening.

o. Platelet count $< 100 \times 10^9$/L or $> 1200 \times 10^9$/L at the Screening.

p. Hemoglobin < 8 g/dL at the Screening.

q. Abnormal alanine aminotransferase (ALT) and/or aspartate aminotransferase (AST) ≥ 2 times the upper limit of normal for the lab at the Screening.

r. Abnormal serum creatinine ≥ 2 times the upper limit of normal for the lab at the Screening.

The above laboratory tests will be allowed to be repeated 1 time if, in the Investigator's clinical judgment, there is a reasonable possibility of the repeat tests not meeting the exclusion values.

s. Systemic or opportunistic fungal infection.

t. Having active tuberculosis, HIV infection, syphilis, or hepatitis B or C based on laboratory data and clinical judgment by the study Investigator.

u. Known active current or history of recurrent infections (including but not limited to herpes zoster, histoplasmosis, coccidiomycosis, but excluding onychomycosis) or any major episode of infection requiring hospitalization or treatment with intravenous injection or oral antibiotics within 4 weeks of the Screening.

Beyond the tests listed above, the remainder of the work-up is at the discretion of the investigator and should be tailored to the clinical situation. Distinguishing between infectious and non-infectious uveitis is part of standard of care and should be dictated by the patient's clinical exam, but at minimum, all patients must have testing for tuberculosis, syphilis and hepatitis prior to enrollment.

v. Prior history of suicide attempt at any time in the subject's lifetime or major psychiatric illness requiring hospitalization within 3 years prior to the Screening.

w. Medical problems or drug or alcohol dependence problems sufficient to prevent adherence to treatment and study procedures.

x. Scheduled surgery or other interventions that would interrupt the subject's participation in the study.

y. Pregnancy or current breast-feeding.

z. Enrolled in other clinical trials.

aa. Any other condition which, in the opinion of the Investigator, would put the subject at risk by participation in the protocol.

4.3 Re-screening of Patients

Individuals that do not meet the above eligibility criteria may be re-screened a minimum of 4 weeks after the last screening visit.

5 Study Procedures

5.1 Informed Consent Procedure

Procedures for obtaining consent include explaining the patient's disease, prognosis, and treatment options, introducing the aims and methods of this trial, discussing the risks and benefits of participation and addressing the patient's questions and concerns Patients will be given at least 24 hours to consider taking part. The subject is assured that participation in this study is voluntary and he/she can withdraw at any time, without giving a reason. A signed informed consent will be obtained from the subject or their legally authorized representative before any study-related procedures are undertaken.

5.2 Eligibility Evaluation

The eligibility evaluation will be performed at the Screening. For detailed inclusion/exclusion criteria see Section 4. Also please see Section 5.7 for assessments at the Screening visit.

If a subject had any item of the results of *Tuberculosis screening, Hepatitis B, Hepatitis C, syphilis and Human immune deficiency virus screening*, *Clinical laboratory tests, Fundus photography, Optical coherence tomography (OCT), B ultrasonography,* and *Ultrasound biomicroscope (UBM)* within 30 days and the results of *Fundus fluorescence angiography (FFA)* within 60 days prior to Screening, and all protocol required documentation is available, this test does not need to be repeated for the items, provided nothing has changed in the subject's medical history to warrant a repeat test.

5.3 Randomization Assignment and Masking

After eligibility has been determined, assignment of a randomization takes place at the Baseline visit. Only subjects who meet all of the inclusion criteria and none of the

exclusion criteria are eligible for randomization into the treatment phase. Randomization will be designed to yield expected assignment ratio of 1:1 to the conventional treatment arm and the biological treatment arm. Randomization will be stratified according to the phase of disease (early or late phase VKH, See Section 6.1) and will be blocked to assure balance across treatment groups. Randomization list will have been generated by a Statistician using a computer and will be properly kept by a Designated Study Coordinator. Both this Statistician and the Study Coordinator will have no involvement in other parts of the trial. The Investigator who is designated as the Primary Treating Ophthalmologist and is responsible for the treatment/management of the subject and the evaluation of the adverse events in this trial will telephone the Designated Study Coordinator to obtained patient assignment information.

Throughout the trial, the treatment assignment will not be masked to the Primary Treating Ophthalmologist. This trial will designate several Investigators as Examining Ophthalmologists who will perform *Visual acuity testing, Tonometry, Perimetry, Fundus photography, Optical coherence tomography (OCT), Fundus fluorescence angiography (FFA), B ultrasonography* and *Ultrasound biomicroscope (UBM)*, and will be masked to treatment assignment to prevent bias in study outcomes.

5.4 Baseline and Subsequent Assessments

The Baseline visit becomes time-point zero from which all other visit dates are calculated. The Screening and the Baseline can be performed on the same day, if all necessary test results are available. The time interval between the Screening and the Baseline should be no more than 10 days. Given this relatively narrow time interval, assessments, auxiliary examinations and laboratory tests already performed in the Screening will not need to be repeated, of which results will be collected and recorded for the Baseline visit. Subjects will initiate the assigned treatment at the Baseline per protocol (See Section 6). Subsequently, subjects will have scheduled clinic visits at

Week 2, Month 2, and every 2 months, until the end of 6 months (Week 26). This trial allows flexible completion of assessment and treatment within the window. Please see for the full study schedule.

Despite the regular scheduled visits, subjects may have unscheduled visits, such as if they experience new ocular or systemic symptoms or adverse events. These additional visits do not fall within the study visit window. The Investigator may carry out other necessary procedures or examinations than those performed in scheduled visits.

5.5 Withdrawn and Lost to Follow-up

Subjects have the right to withdraw from the trial at any time and for any reason, without providing a reason. The investigator also has the right to withdraw participants from the study in the event of inter-current illness, adverse events, protocol violations or other reasons.

If the subject withdraws from the trial, efforts will be made to continue to obtain follow-up data, with the permission of the subject. They will be given appropriate treatment, but will not continue with scheduled study follow-ups unless they allow it.

Subjects will also be considered to have been withdrawn if they have died. If the death is considered to be related to the study drug, the subject will be declared serious adverse event, rather than withdrawn.

Missing a visit does not necessarily mean that a patient has been withdrawn from the study. Some subjects may miss study visits, or do not adhere to the treatment plan, or stop the treatment regimen by self, but they are willing to return for study visits and declare they are interested in further participation. For these subjects, efforts will be made to bring them back as soon as possible. These subjects will be encouraged to return for subsequent study visits according to the protocol, regardless of whether or not they are taking study medication or adhering to the treatment plan. Also, they will be encouraged to resume the assigned treatment if appropriate.

For those subjects fail to attend study visits without stating an intention to withdraw from the study, the Investigator should contact the subject through telephone calls as soon as possible. If contact cannot be established up to the end of the 6-month follow-up period, the subject should be considered as “lost to follow-up” on the date of last contact.

5.6 Study Assessments and Methods

General information and medical history

Complete demographics and medical history will be obtained by the unmasked Primary Treating Ophthalmologist from each subject including date of birth, gender, ethnic background, education background, marriage status, occupation and history of tobacco and alcohol use, any prior ocular history and treatment.

Questionnaires

The EuroQol-5D Questionnaire (EQ-5D) will be used to evaluate the generic quality of life of the subject and The Visual Functioning Questionnaire (VFQ-25) will be used to measure the subject’s vision-related quality of life. Considering that the subject has impaired vision and may be unable to complete the full battery of questionnaires, these questionnaires will read aloud to subjects by a Study Coordinator who is masked to the treatment assignment. The Study Coordinator should never rephrase or interpret questions for the subject, and will record the subject’s answers on the appropriate questionnaires in their original form. These questionnaires should be assessed prior to discussion of the day’s examination results with the participant, to avoid biasing subjective responses by the day’s examination findings.

Vital signs/weight/height

These examinations include sitting blood pressure, heart rate, respiratory rate, body temperature, weight and height and will be performed by a masked Study Nurse.

Physical examination

At the Screening/Baseline, medically qualified, unmasked personnel who routinely do a complete physical exam will perform this assessment. The physical examination at the Baseline visit will serve as the baseline physical examination for the entire study. At all other visits, a symptom directed physical exam will be performed either by the Primary Treating Ophthalmologist or the medically qualified personnel who perform the complete physical exam. Abnormalities noted after the treatment should be evaluated and documented by the Investigator as to whether or not these are adverse events.

Visual acuity testing

With the appropriate corrective lenses based on that subject's refraction, the best corrected visual acuity (BCVA) will be measured using an ETDRS chart using standard operating procedures. The subject’s presenting visual acuity without refraction will also be evaluated using a Logarithmic visual acuity chart. Visual acuity will be obtained prior to pupil dilation. Visual acuity testing will be performed by a masked Examining Ophthalmologist.

Tonometry

Intraocular pressure for both eyes will be measured using a non-contact tonometry. Tonometry should be performed prior to prior to pupil dilation. Tonometry will be performed by a masked Examining Ophthalmologist.

Perimetry

Perimetry will be performed by a masked Examining Ophthalmologist for the exact, quantitative assessment of the extent of space visible in a patient’s periphery. Perimetry will be obtained prior to pupil dilation.

Slit-lamp biomicroscopy

A standard ophthalmic examination using slit lamp biomicroscopy will be performed in both eyes by the unmasked Primary Treating Ophthalmologist. The Anterior chamber cell count will be measured during the examination according to the SUN criteria (see Appendix II), which should be performed prior to pupil dilation.

Ophthalmoscopy

Ophthalmoscopy will be performed by the Primary Treating Ophthalmologist under the dilation of the pupil to determine both vitreous haze grading and the absence/presence of active chorioretinal and/or retinal vascular lesions. Grading of vitreous haze will be based on the publication from the National Eye Institute (NEI) which has also been adapted by the SUN working group (see Appendix III).

Fundus photography

Fundus photography will be performed by a masked Examining Ophthalmologist to obtain documented evidence of the absence/presence of active chorioretinal and/or retinal vascular lesions.

Optical coherence tomography (OCT)

Optical coherence tomography will be performed by a masked Examining Ophthalmologist to determine the central retinal thickness and the presence of macular alternations.

Fundus fluorescence angiography (FFA)

Fundus fluorescence angiography will be performed by a masked Examining Ophthalmologist to determine the absence/presence of active chorioretinal and/or retinal vascular lesions. OCT will be performed by masked Examining Ophthalmologists.

B ultrasonography and ultrasound biomicroscope (UBM)

B ultrasonography and ultrasound biomicroscope may be performed at the

Investigators' discretion according to the clinical needs and will not be mandatory during the trial. These two auxiliary examinations will be carried out by masked Examining Ophthalmologists who will write the examination reports.

Tuberculosis screening

Both PPD test and T-SPOT.TB test are eligible for the TB screening. Those subjects with a positive PPD or T-SPOT.TB need to additionally undertake the chest computed tomography to exclude active tuberculosis.

Human immune deficiency virus (HIV) screening

HIV tests will be performed based on an ELISA (enzyme-linked immunosorbent assay) technique, which seeks to detect both antigens (p24) and antibodies.

Hepatitis B, Hepatitis C and syphilis screening

Syphilis serological tests, hepatitis B surface antigen and hepatitis C antibody tests, (and if necessary, HBV-DNA PCR) will be performed to exclude active hepatitis B, hepatitis C and syphilis.

Clinical laboratory tests

Laboratory evaluations will be performed including Routine blood test (Hematocrit, Hemoglobin, Red Blood Cell count, White Blood Cell count, Neutrophils, Lymphocytes, Monocytes, Basophils, Eosinophils, Platelet count), Biochemical test (Blood Urea Nitrogen [BUN], Creatinine, Uric acid, Serum albumin, Total protein, Total bilirubin, Serum glutamic-pyruvic-transaminase [SGPT/ALT], Serum glutamic-oxaloacetic-transaminase [SGOT/AST], Alkaline phosphatase, Sodium Potassium, Calcium, Inorganic phosphorus), C-reactive protein and erythrocyte sedimentation rate. The urine pregnancy test will be performed if subjects have a childbearing potential. Specimens for Clinical laboratory tests will be collected according to local hospital or lab requirement and will be destroyed after testing.

5.7 Study Schedule Overview

	Screening	Baseline	Week 2	Week 9 (Month 2)	Week 17 (Month 4)	Week 26 (Month 6)	Unscheduled visit
Visit No.	0	1	2	4	5	6	
Visit window (day)	-10	0	±7	±14	±14	±21	
Informed consent	×						
Inclusion/exclusion criteria	×						
Randomization		×[a]					
General information and medical history	×						
Pregnancy test in women of child bearing potential	×						
Questionnaires		×[a]				×	
Vital signs/weight/height	×		×	×	×	×	×
Physical examination	×		×	×	×	×	×
Visual acuity testing	×		×[d]	×	×	×	×[d]
Tonometry	×		×	×	×	×	×
Perimetry	×			×	×	×	
Slit-lamp biomicroscopy	×	×[b]	×	×	×	×	×
Ophthalmoscopy	×	×[b]	×	×	×	×	×
Fundus photography	×			×	×	×	
Optical coherence tomography	×			×	×	×	
Fundus fluorescence angiography	×			×	×	×	
B ultrasonography	×[e]			×[e]	×[e]	×[e]	
Ultrasound biomicroscope	×[e]			×[e]	×[e]	×[e]	
Tuberculosis screening	×						
HIV screening	×						
Hepatitis B screening	×						
Hepatitis C screening	×						
Syphilis testing	×						
Routine blood test	×		×	×	×	×	×[e]
Biochemical test	×		×	×	×	×	×[e]
C-reactive protein		×[a]		×	×	×	
Erythrocyte sedimentation rate		×[a]		×	×	×	
Treatment and record		×	×	×	×	×	×
Protocol deviation evaluation			×	×	×	×	×

	Screening	Baseline	Week 2	Week 9 (Month 2)	Week 17 (Month 4)	Week 26 (Month 6)	Unscheduled visit
Visit No.	0	1	2	4	5	6	
Visit window (day)	-10	0	±7	±14	±14	±21	
Adverse event evaluation		×[c]	×	×	×	×	×

[a] Should be performed before the initiation of treatment.
[b] Will not be repeated if the Screening and Baseline are on the same day.
[c] Will be assessed after the initiation of treatment.
[d] Will be assessed with a Logarithmic visual acuity chart only.
[e] Will be at the Investigators' discretion according to the clinical needs.

6 Treatment Plan

6.1 Stratification

Before randomization, the phase of VKH disease will be determined. The subject will be diagnosed with early or late phase VKH disease according to the Diagnostic Criteria (see Appendix I).

Subjects will be randomized in a 1:1 ratio to receive either conventional or biological treatment regimens using the phase of VKH disease as stratification factor to ensure the balance of this factor between the two arms.

6.2 Corticosteroids

Systemic corticosteroids will be implemented for both arms in the same dose and duration.

The initial dose of corticosteroids (oral prednisone) for early and late phase VKH disease is summarized as follows:

Initial dose of oral prednisone

**Phase**	**Dose [a]**
Early phase VKH disease	0.6-0.8 mg/kg/day (or 30-40 mg/day)
Late phase VKH disease	0.4-0.6 mg/kg/day (or 20-30 mg/day)

[a] An equipotent dose of an alternative oral corticosteroid medication can be used if a specific contraindication to prednisone exists.

The initial dose of corticosteroids will be used for 1 or 2 weeks and then gradually tapered. The daily dose of corticosteroids will be decreased by 5 mg every 1-2 weeks, with a goal of tapering and holding at 15 mg until the end of 6 months.

6.3 Conventional Treatment Regimen

In combination with corticosteroids, regardless of early or late phase VKH disease,

subjects will be orally treated with cyclosporine at a dose of 2-4 mg/kg/day (or 100-200 mg/day) throughout the trial.

If subjects are diagnosed with early-phase VKH disease but without exudative retinal detachment, Investigator may consider using the corticosteroid treatment alone rather than in combination with cyclosporine. But in this case, it will not be mandatory to use only corticosteroids for the treatment of disease. The Investigator has the option to initiate the cyclosporine at any time throughout the trial, depending on patient's condition and Investigator's judgment.

Step-up Treatment

For subjects initially randomized to the conventional treatment arm, a step-up treatment may be considered if patients have an inadequate response to the treatment meeting one of the following criteria in at least one eye:

a. Two-step increase in anterior chamber cells relative to the Baseline throughout the study period (See SUN criteria in Appendix II). This is represented by a change of Grade 0 to Grade 2+; or Grade 0.5+ to Grade 3+.
b. Two-step increase in vitreous haze relative to the Baseline throughout the study period (See NEI/SUN criteria in Appendix III). This is represented by a change of Grade 0 to Grade 2+; or Grade 0.5+ to Grade 3+.
c. New active, inflammatory chorioretinal and/or inflammatory retinal vascular lesions relative to the Baseline.

The Investigator has the option to determine which step-up treatment may be implemented, including but not limited to adding another immunosuppressive drug (chlorambucil, oral, 0.1 mg/kg/day) or transition to biological treatment (adalimumab, subcutaneous injection, 40 mg once every 2 weeks).

6.4 Biological Treatment Regimen

Adalimumab

In combination with corticosteroids, all subjects randomized to the biological

treatment arm will receive subcutaneous injection of adalimumab at a dose of 40 mg once every 2 weeks throughout the trial.

6.5 Other Adjunctive Treatments

Adjunctive treatments described below can be used for subjects in both arms, and all these adjunctive treatments should be recorded on the case report form (CRF) at each visit, if implemented. Subjects can use different doses of topical corticosteroid drops into the study. To prevent side effect (such as increased intraocular pressure), topical corticosteroid drops cannot be used for a long term and should be gradually tapered to completely discontinue the topical use of corticosteroids, if the active anterior inflammation is controlled. Topical adjunctive medications such as cycloplegic agent will be permitted during the study, to be used according to best medical judgment. Intraocular pressure lowering medications, including but not limited to eye drops, drugs and even surgery, will be performed for patients with intraocular pressure elevation and glaucoma secondary to uveitis itself, its complications or induced by corticosteroid, if necessary. Unless there is a contraindication, the occurrence and progression of cataract will be evaluated and properly treated by surgery, YAG laser capsulotomy or surgical capsulotomy during the study period. Supplementation of vitamin D, calcium, or potassium can be implemented in the study to minimize the incidence of adverse events caused by corticosteroids. Patients developing hypertension or diabetes from systemically-administered drugs will be referred to their primary care physician for appropriate management. All adverse events will be noted and reported through the study.

6.6 Prohibited Therapy

The introductions of following medications for VKH disease are prohibited during this clinical trial:

- All other biologic therapies.

- All other immunosuppressive therapies (not including step-up treatment, cyclosporine and chlorambucil).
- Systemic or topical non-steroidal anti-inflammatory drugs.
- Live attenuated vaccines.
- Anti-retroviral therapy.
- Periocular or intravitreal corticosteroid injection.
- Glucocorticosteroid implant.
- Anti-VEGF intravitreal injection.

7 Outcome Assessments

7.1 Primary Outcome

The primary outcome will be the change in BCVA letter score measured using an ETDRS chart from Baseline to the end of 26 weeks.

7.2 Secondary Outcomes

a. Change in BCVA ETDRS letter score from Baseline to the end of 2 months and 4 months.

b. Proportion achieving a ≥ 15 ETDRS letter improvement (appreciable visual gain), a ≥ 10 letter improvement at the end of 2 months, 4 months and 6 months.

c. Change in visual field mean deviation score from Baseline to the end of 26 weeks.

d. Change in anterior chamber cells from the Baseline to the end of 26 weeks.

e. Change in vitreous haze from the Baseline to the end of 26 weeks.

f. Percent change in the presence of retinal detachment from Baseline to the end of 26 weeks.

g. Changes in OCT anatomical features from the Baseline to the end of 26 weeks.

h. Proportion receiving a step-up treatment in the conventional treatment group throughout the study period.

i. Change in EQ-5D scores from the Baseline to the end of 26 weeks.

j. Change in VFQ-25 composite score from the Baseline to the end of 26 weeks.

7.3 Safety Outcomes

Safety and tolerability as defined by the following:

- Type, frequency, severity, and relationship of the adverse events (AEs) to study

drugs.

- Number of subjects who prematurely discontinue study drugs due to any AE.
- Frequency of clinically significant changes in vital signs, and/or laboratory findings.

7.4 Pre-specified Subgroup Analyses

To determine differences between arms in mean change in BCVA ETDRS letter score at 26 weeks across subgroup variable defined by early or late phase VKH disease.

7.5 Cost Effectiveness

The incremental cost-effectiveness ratio calculation is the difference in costs divided by the difference in the number of quality adjusted life years (QALYs) that result from the two treatments over the time period being described:

[Cost(Conventional)-Cost(Biological)]/[QALYs(Conventional)-QALYs(Biological)]

When we use measures other than QALYs, we will also calculate the dollars spent per visual acuity outcome improvement (taking the methodological limitations into account in interpreting this result).

8 Adverse Events

8.1 Adverse Event Reporting

An adverse event (AE) is defined as any unfavorable medical occurrence in a subject who has ever received study medication, regardless of a causal relationship with this treatment. Any worsening of a pre-existing condition or illness should be considered an adverse event. Worsening in severity of a reported adverse event should also be reported as a new adverse event. An elective surgery/procedure scheduled to occur during a study will not be considered an adverse event if the surgery/procedure is being performed for a pre-existing condition and the surgery/procedure has been pre-planned prior to study entry. However, if the pre-existing condition deteriorates unexpectedly during the study (e.g., surgery performed earlier than planned), then, the deterioration of the condition for which the elective surgery/procedure is being done will be considered an adverse event. For adverse events to be considered intermittent, the events must be of similar nature and severity.

The independent Investigator will monitor each subject for clinical and laboratory evidence of adverse events on a routine basis throughout the study. The Investigator will assess and record any adverse event in detail including the date of onset, event diagnosis (if known) or sign/symptom, severity, time course, duration and outcome, relationship of the adverse event to study drug and any actions taken. All these information on non-serious and serious adverse events will be recorded on the CRF at each study visit.

8.2 Serious Adverse Events

Serious adverse events (SAE) should be reported to Chief Investigator and Ethics Committee within 24 hours of the Investigator being made aware of the SAE. Serious adverse events include any medical occurrence that results in the following outcomes:

Death.

Life-threatening experience.

Non-elective surgery or hospitalization for any reason.

Congenital anomaly or birth defect.

Persistent or significant disability or incapacity.

Any other important medical events, not immediately life-threatening or resulting in death or hospitalization but requiring medical or surgical intervention to prevent serious outcome.

8.3 Severity Assessments

The severity of adverse event can be assessed according to the following criteria:

Mild: The adverse event does not interfere with the volunteer's daily routine, and does not require intervention; it causes slight discomfort.

Moderate: The adverse event interferes with some aspects of the volunteer's routine, or requires intervention, but is not damaging to health; it causes moderate discomfort.

Severe: The adverse event results in alteration, discomfort or disability which is clearly damaging to health.

8.4 Causality Assessments

The Investigator should assess the relationship of the adverse event with the use of study medication by reporting according to the three-level classification of "associated", "not associated", and "inability to determine". Causality assessments will be required for study drugs as well as other adjunctive treatments.

8.5 Uveitis-Related Events

The following events are known complications related to the condition being treated

and will be classified as uveitis-related events. These events will be analyzed separately from other adverse events in the final study report. Notably, the following events may not be a complete list of all potential uveitis-related events. The Investigators must determine if a specific event is uveitis-related.

Loss of transparency of the cornea.

Band keratopathy.

Synechiae.

Cataracts.

Glaucoma/increased intraocular pressure.

Vitreous hemorrhage.

Macular edema.

Retinal detachment.

Epiretinal membrane.

Vitreo-macular traction.

Retinal ischemia.

Vision loss.

Hypotony.

8.6 Pregnancy

Subjects who become pregnant during the study period must be discontinued. Pregnancy in a study subject is not considered as an adverse event. However, the medical outcome of an elective or spontaneous abortion, stillbirth or congenital anomaly is considered as a serious adverse event.

8.7 Management

For the purpose of medical management, all adverse events and laboratory abnormalities that occur during the study must be evaluated by the Investigator. The occurrence of adverse events does not necessarily mean the time for the subject to exit the study. But, a serious adverse event will absolutely result in the discontinuation of the study immediately. For those developing non-serious adverse events, reducing the medication dose, administration of other adjunctive treatments or even temporarily stopping the medication may be used. Study medication may be restarted, even allowed in a modified dose, once the Investigator determines that the abnormalities have been successfully dealt with. Otherwise, at this point, patients will be recommended to withdraw from the study and be treated according to the best medical judgment of their clinician.

9 Data Collection and Management

9.1 Confidentiality

Participants will be identified via a unique ID. Identifiable information will not be stored in the eCRF and will not leave the site. Any participant contact information will be stored within the site on password protected computers or within secured locations with limited access. All study data and site files will be kept at site in a secure location with restricted access.

9.2 Source and Data Collection

Written informed consent will be obtained prior to screening and any other study specific procedures are performed. The study will employ an eCRF, whereby data will be managed via this system. Source data worksheets will be used for each patient and data will be entered onto the eCRF database. Source data worksheets will be reconciled at the end of the trial with the patients' medical notes in the study site. During the trial, critical clinical information will be written in the medical notes to ensure informed medical decisions can be made in the absence of the study team. Trial related clinical letters will be copied to the medical notes during the trial. The Principal Investigator will provide a signature for CRF once all queries are resolved and immediately prior to database lock.

It will be the responsibility of the Principal Investigator and his team to ensure the accuracy of all data entered in the worksheets and the eCRF are in accordance with Good Clinical Practice. The Principal Investigator will be responsible for ensuring that source data worksheets are filed in a suitably secure location to ensure source data verification can be undertaken throughout the study.

9.3 Quality Assurance

The study will incorporate a range of data management quality assurance functions as

the trial progresses. The Trial Data Manager will provide study training, ongoing study support and will conduct regular monitoring, checking source data for transcription errors. Any necessary alterations to entered data will be date and time stamped within the eCRF. Regular monitoring of study conduct and data collected will be performed to ensure the study is conducted in accordance with GCP.

9.4 Database Lock

Prior to database lock, the Trial Manager will review any outstanding warnings on the eCRF and resolve or close these as appropriate before database lock. Local study personnel should resolve any queries that arise promptly. Once all queries have been resolved no further changes will be made to the database unless specifically requested by the Study Office in response to the Statistician's data checks. The study Principal Investigator will review all the data and provide electronic signature to verify that all the data are complete and correct. At this point, all data will be formally locked for analysis.

9.5 Archiving

Principal Investigators will be responsible for securely archiving local data generated, essential documents and source data in accordance with local requirements, but for at least 5 years from the end of the study.

10 Statistical Considerations

10.1 Study Hypothesis

There is one formal comparison of interest on primary outcome:

Conventional Treatment vs. Biological Treatment

Conventional Treatment is hypothesized to be not inferior to biological Treatment, whereby the mean of change in best corrected ETDRS visual acuity letter score of conventional treatment arm is not worse than that of biological treatment by a margin of seven letters. This margin represents the minimal clinically important difference, based on clinical trial results of pivotal trials of treatments in uveitis and wet macular degeneration.[9]

The null hypothesis, that conventional treatment is inferior to biological Treatment, will be rejected if the estimated 95% confidence interval for the difference in treatment means lies wholly above the seven letter margin.

10.2 Sample Size Calculation

Assuming the standard deviation of the change in best corrected ETDRS visual acuity letter score at 10 for both conventional treatment arm and biological treatment arm, sample size of 44 subjects per arm would provide 90% power to show non-inferiority at a margin of difference less than 7 between these two arms based on a one-side significance level of 0.025. Expecting 20% dropout rate for those subjects lost during the course of the study and for whom no response data will be collected, the final dropout-inflated enrollment would be 55 subjects per group and 110 in total.

10.3 Data Analyses

Analyses will be on both an Intention-to-treat (ITT) basis and a Per Protocol (PP) basis. Safety analyses will be conducted in the safety set which includes all subjects

recruited and received at least one dose of study medication. A detailed statistical analysis plan will be prepared and finalized prior to database lock.

10.4 Interim Analysis

This study does not plan to perform interim analysis.

11 Ethical Considerations

This clinical trial will be conducted in compliance with the approved protocol, the Helsinki Declaration, the Good Clinical Practice (GCP) guidelines issued by the CFDA, and corresponding regulations.

Before the commencement of this experiment, the approval of the ethics committee must be obtained.

The Investigator is expected to take any immediate action required for the safety of any patient included in this study, even if this action represents a deviation from the protocol.

During the clinical study, any changes made to this trial protocol should be reported to the Ethics Committee and placed on record.

Subjects must provide informed consent to participate in the trial.

If the subject and his or her legal representative are illiterate, the informed consent process shall be attended by a witness, who shall sign the informed consent form after oral consent by the subject or his or her legitimate representative.

A copy of the informed consent form and the contact information for the researcher and the ethics committee will be provided to the patient.

The study site must be equipped with the necessary medical rescue equipment and first aid drugs for emergency issues.

REFERENCES

1. Du L, Kijlstra A, Yang P. Vogt-Koyanagi-Harada disease: Novel insights into pathophysiology, diagnosis and treatment. Prog Retin Eye Res 2016;52:84-111.

2. Zhong Z, Su G, Kijlstra A, Yang P. Activation of the interleukin-23/interleukin-17 signalling pathway in autoinflammatory and autoimmune uveitis. Prog Retin Eye Res 2020;16:100866.

3. Yang P, Ren Y, Li B, Fang W, Meng Q, Kijlstra A. Clinical characteristics of Vogt-Koyanagi-Harada syndrome in Chinese patients. Ophthalmology 2007;114:606-14.

4. Dick AD, Rosenbaum JT, Al-Dhibi HA, et al. Guidance on Noncorticosteroid Systemic Immunomodulatory Therapy in Noninfectious Uveitis: Fundamentals Of Care for UveitiS (FOCUS) Initiative. Ophthalmology 2018;125:757-73.

5. Yang P, Ye Z, Du L, et al. Novel treatment regimen of Vogt-Koyanagi-Harada disease with a reduced dose of corticosteroids combined with immunosuppressive agents. Curr Eye Res 2018;43:254-61.

6. Jabs DA, Rosenbaum JT, Foster CS, et al. Guidelines for the use of immunosuppressive drugs in patients with ocular inflammatory disorders: recommendations of an expert panel. Am J Ophthalmol 2000;130:492-513.

7. Jaffe G, Dick A, Brézin A, et al. Adalimumab in patients with active noninfectious uveitis. N Engl J Med 2016;375.

8. Nguyen QD, Merrill PT, Jaffe GJ, et al. Adalimumab for prevention of uveitic flare in patients with inactive non-infectious uveitis controlled by corticosteroids (VISUAL II): a multicentre, double-masked, randomised, placebo-controlled phase 3 trial. Lancet 2016;388:1183-92.

9. Writing Committee for the Multicenter Uveitis Steroid Treatment Trial, Follow-up Study Research Group, Kempen JH, et al. Association Between Long-Lasting Intravitreous Fluocinolone Acetonide Implant vs Systemic Anti-inflammatory Therapy and Visual Acuity at 7 Years Among Patients With Intermediate, Posterior, or Panuveitis. JAMA 2017;317:1993-2005.

10. Yang P, Zhong Y, Du L, et al. Development and evaluation of diagnostic criteria for Vogt-Koyanagi-Harada disease. JAMA Ophthalmol 2018;136:1025-31.

Appendix I

Diagnostic Criteria for Vogt-Koyanagi-Harada (VKH) Disease

A. No history of penetrating ocular trauma or intraocular surgery preceding the initial onset of uveitis

B. Bilateral ocular involvement (time interval between the 2 eyes should be 2 wk)

C. No evidence of infectious uveitis or accompanying systemic rheumatic diseases or evidence suggestive of other ocular disease entities[a]

D. Early-phase VKH disease:

1. Signs of diffuse choroiditis and exudative retinal detachment
2. Serous retinal detachment on OCT or B-scan ultrasonography
3. Choroidal thickening on EDI-OCT[b]
4. Early punctate staining and late subretinal dye pooling on FFA
5. Hyperfluorescence of the optic disc on FFA

Definite diagnosis:

Variant 1: In patients presenting with A + B + C + D(1)

Variant 2: In patients without clinically visible exudative retinal detachment, ie, A + B + C + D(2) + D(3) or A + B + C + D(4)

Variant 3: In patients already treated with systemic corticosteroids or combined with other immunosuppressive agents, a history of typical appearances of variant 1 or 2, and A + B + C + D(5)

E. Late-phase VKH disease

1. Signs of definite sunset glow fundus or retinal pigment epithelium clumping/migration
2. Signs of bilateral recurrent granulomatous anterior uveitis
3. Signs of Dalen-Fuchs nodules or multifocal chorioretinal atrophy
4. Window defects/moth-eaten fluorescence on FFA
5. Previous history of characteristic findings corresponding to diagnosis of early-phase VKH disease

Definite diagnosis:

Variant 1: In patients presenting with A + B + C + E(1) + E(2)

Variant 2: In patients without sunset glow fundus or visible pigment alternations due to early and appropriate treatment, ie, A + B + C + E(2) + E(3) or A + B + C + E(2) + E(4)

Variant 3: In patients with significant media opacity, ie,

A + B + C + E(2) + E(5)

Abbreviations: EDI, enhanced depth imaging; FFA, fluorescence fundus angiography; OCT, optical coherence tomography.

[a] This criterion includes (1) no history nor clinical evidence to show ocular tuberculosis, syphilis, or ocular toxoplasmosis; (2) no underlying systemic rheumatic disease that could explain the form of uveitis these patients have; and (3) no history or clinical evidence to suggest the possibility of a specific entity, for instance intraocular tumors, toxic uveitis, Fuchs syndrome, or Posner-Schlossman syndrome.

[b] Ultrasonography can be used to detect the choroidal thickening and therefore may serve as an alternative in the examination where the EDI-OCT is not available, although it is less precise.

From Yang P et al. Development and Evaluation of Diagnostic Criteria for Vogt-Koyanagi-Harada Disease. JAMA Ophthalmol. 2018;136(9):1025-1031.

Appendix II

SUN Working Group Grading System for Anterior Chamber Cells

Grade	Number of cells in field [a]
0	<1
0.5+	1-5
1+	6-15
2+	16-25
3+	26-50
4+	>50

[a] Field size is a 1mm by 1mm slit lamp beam. Grading should be done with the highest magnification and illumination and conducted in a completely dark room, prior to dilation.

From Jabs DA et al. Standardization of uveitis nomenclature for reporting clinical data. Results of the First International Workshop. Am J Ophthalmol 2005;140(3):509-16.

Appendix III

National Eye Institute (NEI)/SUN Criteria for Grading Vitreous Haze

Grade	Description [a]
0	No evident vitreal haze
0.5+	Slight blurring of the optic disc margin because of the haze; normal striations and reflex of the nerve fiber layer cannot be visualized
1+	Permits a better definition of both the optic nerve head and the retinal vessels (compared to higher grades)
2+	Permits better visualization of the retinal vessels (compared to higher grades)
3+	Permits the observer to see the optic nerve head, but the borders are quite blurry
4+	Optic nerve head is obscured

[a] Grading should be conducted in a completely dark room.

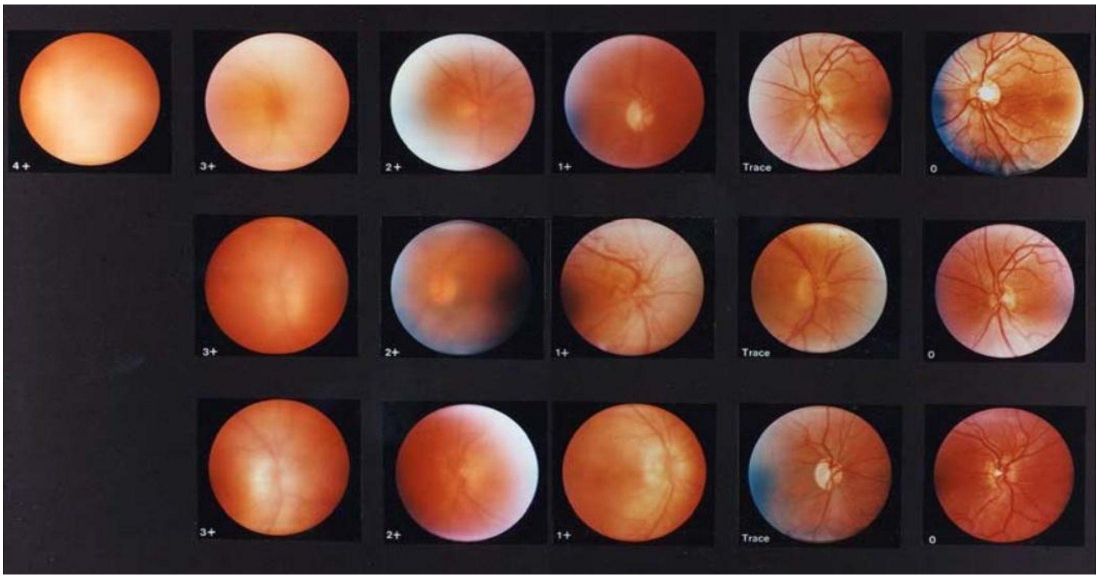

From Nussenblatt et al. Standardization of vitreal inflammatory activity in intermediate and posterior uveitis. Ophthalmology 1985;92:467-471.

Statistical Analysis Plan

Conventional versus Biological Treatment for Vogt-Koyanagi-Harada Disease

A Randomized, Open-label, Blinded-endpoint, Non-inferiority Trial

Version 1.0 December 20th, 2021

The First Affiliated Hospital of Chongqing Medical University

Chongqing Key Laboratory of Ophthalmology and Chongqing Eye Institute

Chongqing, China

CONTENTS

1 Introduction....4
2 Study Objective....4
2.1 Primary Objective....5
2.2 Secondary Objectives....5
2.3 Safety Objectives....5
3 Study Outcomes....5
3.1 Primary Outcome....5
3.2 Secondary Outcomes....5
3.3 Safety Outcomes....6
3.4 Cost Effectiveness....6
4 Statistical Hypothesis....6
5 Sample Size....7
6 Randomization....7
7 Analysis Population....8
7.1 Intention-To-Treat Population....8
7.2 Per Protocol Population....8
7.3 Safety Population....9
8 Missing Data....9
9 Baseline Characteristic Analysis....9
10 Primary Effectiveness Comparison....10
11 Analysis of Secondary Outcomes....11
11.1 General Principles....11
11.2 Analytical Methods....11
12 Safety Analysis....13
13 Subgroup Analysis....13
14 Planned Sensitivity Analysis....14
14.1 Adjustment for Prednisone Dose....14
14.2 Adjustment for Ocular Surgery....14

14.3 Adjustment for Full Covariates 14
14.4 Modified Intention-To-Treat Analysis 14
14.5 Limited to Higher ETDRS Score 15
14.6 Two-Stage Pooled Analysis 15
14.7 Multiple Imputation 15
14.8 Excluding Effects of Step-up Treatment 16
15 Interim Analysis 16
16 Statistics Software 16
References 16

1 Introduction

This is an investigator-initiated, parallel group-randomized, open-label, blinded-endpoint, non-inferiority clinical trial to compare the clinical effectiveness of conventional and biological treatment strategies/regimens for subjects with Vogt-Koyanagi-Harada (VKH) disease and who have an active inflammation within the last 90 days in any eye and/or require chronic oral prednisone ≥ 20 mg/day for the treatment for intraocular inflammation.

Approximately 110 eligible subjects will be randomized 1:1 (55 subjects per group) to receive either conventional treatment regimen or biological treatment regimen using the disease phase (early or late phase VKH) as the stratification factor. Classification of disease phase will be based on the Chinese Criteria.[1] All participants will be followed up for 26 weeks since randomization. The primary outcome will be the difference in mean change in Early Treatment Diabetic Retinopathy Study (ETDRS) BCVA letter score from baseline to 26 weeks. The ETDRS score will be measured in a blinded manner. The minimal clinically important difference (MCID) for change in letters read is 7 letters. This trial aims to test whether the mean change in ETDRS BCVA letters from randomization to Week 26 is not different by more than a margin of -7 letters.

The statistical analysis plan (SAP) documents the planned statistical analyses for the trial and is based on the protocol, together with any subsequent amendments. The SAP is intended for the use of project team members and should be read in conjunction with the aforementioned protocol. The analyses that are detailed in this SAP will be performed only when the database has been locked and all protocol violators identified. Analysis populations will be determined using the rules prespecified in this SAP. At a date to be agreed within the project team, a data look and further analysis will be performed.

2 Study Objective

2.1 Primary Objective

To determine whether the conventional treatment is non-inferior to the biological treatment in improving the best corrected visual acuity (BCVA) at 26 weeks among patients with Vogt-Koyanagi-Harada disease.

2.2 Secondary Objectives

To compare clinical effectiveness, clinical outcomes, cost-effectiveness and patient reported outcomes (PROs) between the conventional and biological treatment groups in Vogt-Koyanagi-Harada disease.

2.3 Safety Objectives

To evaluate the safety and tolerability of the conventional versus biological treatment for Vogt-Koyanagi-Harada disease.

3 Study Outcomes

3.1 Primary Outcome

The primary outcome will be the change in BCVA letter score measured using an ETDRS chart from Baseline to the end of 26 weeks.

3.2 Secondary Outcomes

a. Proportion achieving a $\geq$ 15 ETDRS letter improvement (appreciable visual gain) at the end of 26 weeks.

b. Change in visual field indices (visual field index, mean deviation and pattern standard deviation) from Baseline to the end of 26 weeks.

c. Proportion of participants achieving an inactive uveitis (an anterior chamber cell grade of 0.5+ or less, a vitreous haze grade of 0.5+ or less, and no active inflammatory choroidal or retinal lesions) in both eyes at the end of 26 weeks.

d. Percent change in the presence of retinal detachment from Baseline to the end of

26 weeks.

e. Changes in OCT anatomical features (central macular thickness) from the Baseline to the end of 26 weeks.

f. Proportion receiving a step-up treatment in the conventional treatment group throughout the study period.

g. Change in EQ-5D scores from the Baseline to the end of 26 weeks.

h. Change in VFQ-25 composite score from the Baseline to the end of 26 weeks.

3.3 Safety Outcomes

Safety and tolerability as defined by the following:

- Type, frequency, severity, and relationship of the adverse events (AEs) to study drugs.
- Number of subjects who prematurely discontinue study drugs due to any AE.
- Frequency of clinically significant changes in vital signs, and/or laboratory findings.

3.4 Cost Effectiveness

The incremental cost-effectiveness ratio calculation is the difference in costs divided by the difference in the number of quality adjusted life years (QALYs) that result from the two treatments over the time period being described:

[Cost(Conventional)-Cost(Biological)]/[QALYs(Conventional)-QALYs(Biological)]

When we use measures other than QALYs, we will also calculate the dollars spent per visual acuity outcome improvement (taking the methodological limitations into account in interpreting this result).

A detailed cost-effectiveness analysis plan will be prepared and finalized in additional documents and is not presented in this document.

4 Statistical Hypothesis

There is one formal comparison of interest on primary outcome:

Conventional Treatment vs. Biological Treatment

Conventional Treatment is hypothesized to be not inferior to biological Treatment, whereby the mean of change in best corrected ETDRS visual acuity letter score of conventional treatment arm is not worse than that of biological treatment by a margin of -7 letters. This margin represents the minimal clinically important difference, based on clinical trial results of pivotal trials of treatments in uveitis and wet macular degeneration. [2]

The null hypothesis, that conventional treatment is inferior to biological Treatment, will be rejected if the estimated 95% confidence interval for the difference in treatment means lies wholly above the -7 letter margin.

5 Sample Size

Assuming the standard deviation of the change in best corrected ETDRS visual acuity letter score at 10 for both conventional treatment arm and biological treatment arm and a true difference of 0 between two treatment arms in mean changes, sample size of 44 subjects per arm will provide 90% power to show non-inferiority at a margin of difference less than -7 between these two arms based on a one-side significance level of 0.025. Expecting 20% dropout rate for those subjects lost during the course of the study and for whom no response data will be collected, the final dropout-inflated enrollment will be 55 subjects per group and 110 in total.

6 Randomization

Randomization will be designed to yield expected assignment ratio of 1:1 to the conventional treatment arm and the biological treatment arm. Randomization will be stratified according to the phase of disease (early or late phase VKH) and will be blocked to assure balance across treatment groups. Randomization list will have been generated by a Statistician using a computer and will be properly kept by a

Designated Study Coordinator. Both this Statistician and the Study Coordinator will have no involvement in other parts of the trial. The Investigator who is designated as the Primary Treating Ophthalmologist and is responsible for the treatment/management of the subject will telephone the Designated Study Coordinator to obtained patient assignment information. The Investigators will be aware of patient treatment assignment; however, they will not have access to the randomization lists at the site.

7 Analysis Population

7.1 Intention-To-Treat Population

The Intent-To-Treat (ITT) Population will consist of all patients who had been randomized to a treatment group in the study regardless of their compliance with the study medication. For all ITT analyses, participants will be analyzed as randomized.

Of note, data of participants withdrawing consent to medication intake but not to data collection are planned to be included in the intention-to-treat analysis.

7.2 Per Protocol Population

The Per Protocol Population will consist of eligible patients who have completed the entire planned treatment with no major protocol deviations and where outcomes have been observed.

Participant data will be examined for evidence of protocol deviations in order to assess how well the protocol is followed. Protocol deviations are classified prior to database lock. The following are predefined major protocol deviations:

- Non-adherence to treatment: Adherence is defined as taking all doses of the prescribed drugs over the scheduled duration. Non-adherent participants, as in participants taking prohibited drugs or not taking all the anticipated doses, will not be included in the per-protocol analysis.

- Incomplete data collection: Data on primary outcome are not measured. This condition (incomplete data collection) also includes participants who are lost to follow up or withdraw from the trial before the completion of the trial. Of note, participants who withdraw and stop their assigned treatment but are willing to allow further data collection or follow up will not be included in the per-protocol analysis.

7.3 Safety Population

All patients who receive at least one dose of study drug according to the study protocol and safety assessment available will be included in the safety population. This is the main population for the safety evaluation of this study.

8 Missing Data

For baseline characteristics, missing values will be expected to be rare, and will be imputed with means or medians, as appropriate. In the primary analysis, for the primary outcome, missing data will be imputed with the last observation carried forward (LOCF). There is no plan to impute missing data for secondary outcomes. Analyses on secondary outcomes will be performed in populations where outcomes are observed.

9 Baseline Characteristic Analysis

A CONSORT flow diagram[3] will be used to summarize the following information:

-Number of people assessed for eligibility according to predefined criteria

-Number of ineligible people screened

-Number of eligible people randomized

-Number of participants excluded due to ineligibility post-randomization (incl. reasons)

-Number of participants randomized undergoing intervention

-Number participants with protocol deviations, lost to follow-up, or withdrawal (incl. reasons)

-Number participants in each analysis population

In general, baseline characteristics will be presented descriptively in the intention-to-treat population. According to the numerical characteristics of the variables, if the data follow a normal distribution, the mean with standard deviation will be used for statistical description; if the data are skewed, the median with interquartile range will be used for statistical description. Categorical variables will be summarized as numbers with percentages. Tests of statistical significance will not be taken for baseline characteristics.

Descriptive summaries of clinical variables will include age, gender, body mass index, disease phase, systemic symptoms and signs, EQ-5D score, VFQ-25 composite score, daily prednisone dose, cumulative or mean daily dose of prednisone throughout 26 weeks, ETDRS score per eye, intraocular pressure per eye, status of retinal detachment, central macular thickness per eye, and visual field indices per eye.

10 Primary Effectiveness Comparison

The primary outcome, change in BCVA ETDRS letter score from baseline to the end of 26 weeks, will be analyzed with data of the unit of eye level. The primary effectiveness comparison will be performed in the Intent-To-Treat population based on randomized treatment assignment. The primary analysis will be repeated on the Per Protocol population based on the treatment received as a supportive analysis. This analysis for the treatment group comparison will utilize a generalized estimating equation (GEE) to account for the correlation between eyes of the same patient, baseline BCVA ETDRS score per eye and disease phase (randomization stratification factor: early vs. late VKH disease). Least-squares mean changes by treatment group will be estimated with the GEE model, and the estimated between-group difference in mean changes and 97.5% one-sided confidence interval (CI) will be compared to the

non-inferiority margin (-7 letters) with the use of the Wald test. We will conclude that the conventional treatment is non-inferior to the biological treatment when the entire 97.5% one-sided confidence interval is greater than -7, which is equivalent to a one-sided P value of 0.025 or less indicating statistical significance of non-inferiority (Figure 1).

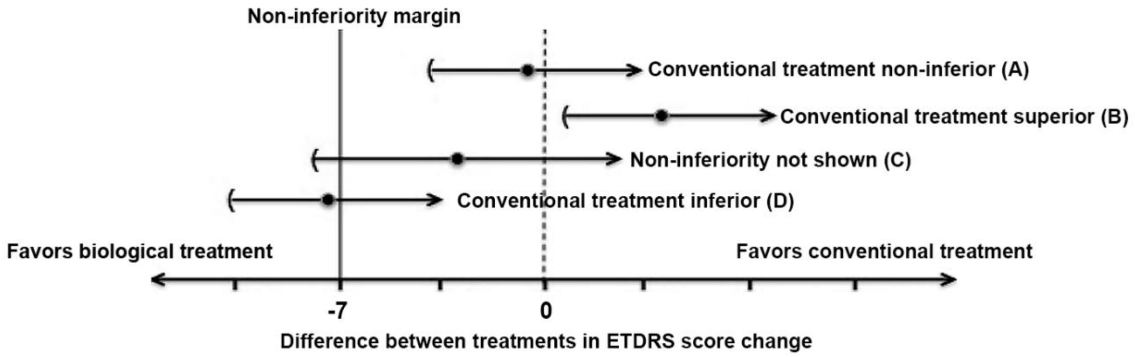

Figure 1. Trial Conclusion Diagram

11 Analysis of Secondary Outcomes

11.1 General Principles

The analysis of secondary outcomes will be performed by using an Intent-To-Treat framework within populations whose secondary outcome measures are available. No formal statistical comparisons for the secondary outcomes are planned, and no adjustment for multiple comparisons will be performed. No inferences can be drawn regarding the secondary outcomes, and these results will be descriptively presented as point estimates with unadjusted 95% confidence intervals only.

11.2 Analytical Methods

a. With accounting for disease phase, a log-binomial regression will be used to analyze the endpoint of achieving a ≥ 15 ETDRS letter improvement (in either eye and in both eyes, respectively) at the end of 26 weeks by treatment group.

b. With accounting for the correlation between eyes of the same patient, baseline value per eye and disease phase, the generalized estimating equation will be used

to estimate the mean change in visual field indices (visual field index, mean deviation and pattern standard deviation) from baseline to the end of 26 weeks by treatment group, respectively.

c. With accounting for disease phase, a log-binomial regression will be used to analyze the endpoint of achieving an inactive uveitis (an anterior chamber cell grade of 0.5+ or less, a vitreous haze grade of 0.5+ or less, and no active inflammatory choroidal or retinal lesions) in both eyes at the end of 26 weeks by treatment group.

d. Percent change in the presence of retinal detachment from Baseline to the end of 26 weeks will be reported by treatment group by calculating (Numbers of eyes with resolution of retinal detachment defined as normalization of macular thickness on OCT at week 26)/(Numbers of eyes with evidence of retinal detachment on OCT at baseline)*100%.

e. With accounting for the correlation between eyes of the same patient, baseline central macular thickness per eye and disease phase, the generalized estimating equation will be used to estimate the mean change in central macular thickness on OCT from baseline to the end of 26 weeks by treatment group.

f. Proportion receiving a step-up treatment in the conventional treatment group throughout the study period will be calculated as (Numbers of patients receiving a step-up treatment)/(Numbers of patients assigned to the conventional treatment group)*100%.

g. With accounting for baseline EQ-5D score and disease phase, the generalized estimating equation will be used to estimate the mean change in EQ-5D score from baseline to the end of 26 weeks by treatment group. We will use a standardized valuation for EQ-5D health state, which was developed for Chinese utility with the use of the time trade-off method.[4]

h. With accounting for baseline VFQ-25 composite score and disease phase, the

generalized estimating equation will be used to estimate the mean change in VFQ-25 composite score from baseline to the end of 26 weeks by treatment group. To calculate an overall composite score for the VFQ-25, we will simply average the vision-targeted subscale scores, excluding the general health rating question.[5]

12 Safety Analysis

All analyses of safety data will be carried out by using the safety population. All safety summaries will be tabulated by MedDRA System Organ Class and by randomized treatment group. Adverse events (AEs) that occur during treatment will be summarized descriptively and will be tabulated as events per 100 patient-years for following categories: any reported AE, AEs leading to death, severe AEs, AEs leading to discontinuation of study drug, AEs rated "associated" with study drug judged by the safety monitors, uveitis-related AEs judged by the safety monitors and AEs of special interest. The AE rate per 100 patient-years will be calculated as ([the total number of AEs reported/the total number of years of study drug exposure])*100. For each treatment group and overall, the numerator of the rate will be the total number of AEs reported that is a patient can be counted more than once overall; while, the denominator of the rates will be the total number of days exposed to study drug summed across all treated subjects divided by 365.25.

13 Subgroup Analysis

Subgroup analysis for the primary effectiveness will be performed on the ITT population. A pre-specified subgroup analysis will be performed to determine differences between treatment groups in mean change in BCVA ETDRS letter score at 26 weeks across subgroup variable defined by early or late phase VKH disease according to the previous criteria.[1] The subgroup analysis will be conducted with the use of the generalized estimating equation (GEE) by additionally including the treatment-by-subgroup interaction term. We will test for heterogeneity of treatment effect across the subgroups and report the corresponding P value for interaction. A

two-sided P value of less than 0.05 on the Wald χ^2 test will be considered to indicate statistical significance for the interaction term. If necessary, additional subgroup analyses will be performed as appropriate by testing other subgroup variables but will be considered exploratory.

14 Planned Sensitivity Analysis

Several sensitivity analyses on the primary outcome will be conducted to assess robustness of trial conclusions.

14.1 Adjustment for Prednisone Dose

A sensitivity analysis will be conducted in the ITT population in which cumulative prednisone dose throughout 26 weeks will be added to the primary analysis GEE model as a covariate.

14.2 Adjustment for Ocular Surgery

Owing to the pragmatic design of the trial, ocular surgeries are permitted during the trial period and are expected to be balanced between groups. A sensitivity analysis will be further conducted in the ITT population, where variables (receiving one specific surgery or not during the trial period) will be added to the primary analysis GEE model as covariates.

14.3 Adjustment for Full Covariates

A sensitivity analysis will be conducted in the ITT population in which cumulative prednisone dose throughout 26 weeks, surgery variables (receiving one specific surgery or not during the trial period), age, sex, baseline body mass index, baseline central macular thickness per eye and baseline visual field indices (visual field index, mean deviation and pattern standard deviation) per eye, will be added to the primary analysis GEE model as covariates.

14.4 Modified Intention-To-Treat Analysis

A sensitivity analysis to assess the primary outcome will be conducted in the modified intention-to-treat (mITT) population which includes all participants who undergo randomization, with the exception of those who do not start any assigned treatment and those who do not have any primary outcome data at week 26. No data imputation will be performed for mITT analysis.

14.5 Limited to Higher ETDRS Score

Since the ETDRS score ranges from 0 to 100 letters which represents the number of letters that can be read by a patient, the ETDRS score may not be able to differentiate the extent of changes within the range of too low vision. The primary outcome will be assessed in a sensitivity analysis only involving eyes with an ETDRS score (20 letters or higher) at baseline of participants in ITT population.

14.6 Two-Stage Pooled Analysis

In a sensitivity involving the ITT population, the primary outcome will be assessed within each subgroup (early vs. late-phase VKH disease) separately using consistent GEE model that accounts for the correlation between eyes of the same patient and baseline BCVA ETDRS score per eye, and then the results will be synthesized from each subgroup using the fixed-effects and random-effects meta-analysis, respectively. Between-subgroup heterogeneity will be statistically assessed with the use of I^2.

14.7 Multiple Imputation

It is expected that there will be lost to follow-up or withdrawal before the end of trial in some patients. We assume that the missing data on the primary outcome of these patients will be missing completely at random, and in a sensitivity analysis multiple imputation will be employed to address the non-response. Missing data on the primary outcome will be predicted with this approach based on available variables including disease phase, age, sex, baseline body mass index, baseline ETDRS score per eye, baseline central macular thickness per eye and baseline visual field indices (visual field index, mean deviation and pattern standard deviation) per eye. The

primary outcome will be assessed in 50 imputed datasets and then combined with the use of the Rubin's rules.

14.8 Excluding Effects of Step-up Treatment

A sensitivity analysis will be conducted in the Per Protocol population, where the visual acuity data at the clinic visit when the step-up treatment is initiated will be assigned to the primary outcome data of participants for analyses, that is, data from participants are censored when the step-up treatment is initiated.

15 Interim Analysis

This study does not plan to perform interim analysis.

16 Statistics Software

IBM SPSS Statistics, version 25.0 software will be used for statistical analysis.

References

1. Yang P, Zhong Y, Du L, et al. Development and evaluation of diagnostic criteria for Vogt-Koyanagi-Harada disease. JAMA Ophthalmol 2018;136:1025-31.

2. Writing Committee for the Multicenter Uveitis Steroid Treatment Trial, Follow-up Study Research Group, Kempen JH, et al. Association Between Long-Lasting Intravitreous Fluocinolone Acetonide Implant vs Systemic Anti-inflammatory Therapy and Visual Acuity at 7 Years Among Patients With Intermediate, Posterior, or Panuveitis. JAMA 2017;317:1993-2005.

3. Moher D, Schulz KF, Altman DG. The CONSORT statement: revised recommendations for improving the quality of reports of parallel-group randomised trials. Lancet 2001;357:1191-4.

4. Liu GG, Wu H, Li M, Gao C, Luo N. Chinese time trade-off values for EQ-5D health states. Value Health 2014;17:597-604.

5. Mangione CM, Lee PP, Gutierrez PR, Spritzer K, Berry S, Hays RD. Development of the 25-item National Eye Institute Visual Function Questionnaire. Arch Ophthalmol 2001;119:1050-8.
