# Supplementary material for: A randomized non-inferiority trial of therapeutic strategy with immunosuppressants versus biologics for Vogt-Koyanagi-Harada disease
Source: Nat Commun. 2023 Jun 24;14:3768. doi: 10.1038/s41467-023-39483-5 (PMC10290648; doi:10.1038/s41467-023-39483-5)
Supplement: Supplementary file 2 — Reporting Summary [file 41467_2023_39483_MOESM2_ESM.pdf]

nature portfolio

Corresponding author(s): Peizeng Yang

Last updated by author(s): May 31, 2023

Reporting Summary

Nature Portfolio wishes to improve the reproducibility of the work that we publish. This form provides structure for consistency and transparency in reporting. For further information on Nature Portfolio policies, see our Editorial Policies and the Editorial Policy Checklist.

Statistics

For all statistical analyses, confirm that the following items are present in the figure legend, table legend, main text, or Methods section.

n/a	Confirmed	
☐	☒	The exact sample size (*n*) for each experimental group/condition, given as a discrete number and unit of measurement
☐	☒	A statement on whether measurements were taken from distinct samples or whether the same sample was measured repeatedly
☐	☒	The statistical test(s) used AND whether they are one- or two-sided<br>*Only common tests should be described solely by name; describe more complex techniques in the Methods section.*
☐	☒	A description of all covariates tested
☐	☒	A description of any assumptions or corrections, such as tests of normality and adjustment for multiple comparisons
☐	☒	A full description of the statistical parameters including central tendency (e.g. means) or other basic estimates (e.g. regression coefficient) AND variation (e.g. standard deviation) or associated estimates of uncertainty (e.g. confidence intervals)
☐	☒	For null hypothesis testing, the test statistic (e.g. *F*, *t*, *r*) with confidence intervals, effect sizes, degrees of freedom and *P* value noted<br>*Give P values as exact values whenever suitable.*
☒	☐	For Bayesian analysis, information on the choice of priors and Markov chain Monte Carlo settings
☒	☐	For hierarchical and complex designs, identification of the appropriate level for tests and full reporting of outcomes
☐	☒	Estimates of effect sizes (e.g. Cohen's *d*, Pearson's *r*), indicating how they were calculated

Our web collection on statistics for biologists contains articles on many of the points above.

Software and code

Policy information about availability of computer code

Data collection	Research Manager (ResMan) Clinical Trial Management Public Platform was used for data collection.
Data analysis	Data were analyzed with the use of IBM SPSS Statistics, version 25.0.

For manuscripts utilizing custom algorithms or software that are central to the research but not yet described in published literature, software must be made available to editors and reviewers. We strongly encourage code deposition in a community repository (e.g. GitHub). See the Nature Portfolio guidelines for submitting code & software for further information.

Data

Policy information about availability of data

All manuscripts must include a data availability statement. This statement should provide the following information, where applicable:

- Accession codes, unique identifiers, or web links for publicly available datasets
- A description of any restrictions on data availability
- For clinical datasets or third party data, please ensure that the statement adheres to our policy

The complete de-identified patient data set are available under restricted access because they contain information of health records and making the data publicly available without additional consent or ethical approval might compromise patient's privacy and the original ethical approval, and data access can be obtained by request from the corresponding author (peizengycmu@126.com). The timeframe for response to requests will be within two weeks. Data will be shared for non-

commercial purposes, with investigator support, after approval of a proposal, and with a signed data access agreement. The trial protocol and statistical analysis plan are provided with the paper. Source data are provided with this paper.

Human research participants

Policy information about studies involving human research participants and Sex and Gender in Research.

Reporting on sex and gender	Findings do not apply to only one sex or gender. Sex and/or gender was not considered in the study design. Sex and/or gender of participants was determined based on self reporting to the treating physician. Difference between sex and gender was not relevant to the topic of the study. No sex- and gender-based analyses have been performed because sex and/or gender was not considered a confounder in this randomized study.
Population characteristics	Subjects were adult patients (≥18 years) who had active VKH disease within the last 90 days and/or had a justification for remission induction with chronic oral prednisone at 20 mg per day or greater. Patients were considered an active disease if they had any of the following manifestations in either eye: anterior chamber cell grade ≥2+; vitreous haze grade ≥2+; or active inflammatory choroidal or retinal lesions detected by optical coherence tomography (OCT) or fluorescein fundus angiography (FFA). Key exclusion criteria included visual acuity of hand motions or worse in the better-seeing eye and previous exposure to anti-TNF therapy. Overall, the mean (±std) age of the participants was 40.3±12.1 years (age range: 18-72 years), and 51 (46.4%) were female. The median duration of disease was 23 days (interquartile range, 12 to 40) for patients with early-phase disease and 17 months (interquartile range, 9 to 43) for those with late-phase disease.
Recruitment	Participants were recruited according to entry criteria outlined in the protocol at the First Affiliated Hospital of Chongqing Medical University to receive either the immunosuppressant therapeutic strategy with oral cyclosporine (N=56) or the biologic therapeutic strategy with adalimumab injection (N=54). Procedures for recruitment included obtaining consent, explaining the patient's disease, prognosis and treatment options, introducing the aims and methods of this trial, discussing the risks and benefits of participation and addressing the patient's questions and concerns. Patients were given at least 24 hours to consider taking part. The subject was assured that participation in this study was voluntary and he/she could withdraw at any time, without giving a reason. A signed informed consent for participation and recruitment was obtained from the subject or their legally authorized representative before any study-related procedures were undertaken. The study was designed as a blinded-endpoint, randomized trial to minimize the bias in the treatment effectiveness estimates.
Ethics oversight	The Ethics Committee of the First Affiliated Hospital of Chongqing Medical University approved the trial.

Note that full information on the approval of the study protocol must also be provided in the manuscript.

Field-specific reporting

Please select the one below that is the best fit for your research. If you are not sure, read the appropriate sections before making your selection.

☒ Life sciences ☐ Behavioural & social sciences ☐ Ecological, evolutionary & environmental sciences

For a reference copy of the document with all sections, see nature.com/documents/nr-reporting-summary-flat.pdf

Life sciences study design

All studies must disclose on these points even when the disclosure is negative.

Sample size	We hypothesized that cyclosporine plus corticosteroids would result in a mean change in Early Treatment Diabetic Retinopathy Study (ETDRS) score that was no less than 7 letters (minimal clinically important difference) lower than the mean change by adalimumab plus corticosteroids. Assuming the true difference of 0 between two treatment groups in mean changes and a standard deviation of 10, we estimated that a sample size of 110 participants, accounting for a dropout rate of 20%, would provide 90% power to show the non-inferiority at a margin of -7 letters at a one-side significance level of 2.5%.
Data exclusions	Effectiveness analyses were based on the intention-to-treat population that comprised all randomized patients as well as on the per protocol population that included the subset of patients who completed a full treatment course without major protocol violations. Safety analyses were based on the safety population that included all those having received at least one dose of assigned treatment.
Replication	Not applicable for clinical data, as we are reporting pre-specified analysis of a phase III clinical trial.
Randomization	Participants were randomly assigned to the biologic (adalimumab) group or the immunosuppressant (cyclosporine) group in a 1:1 ratio, stratified by disease phase (early vs. late) according to the Chinese criteria. Random assignment sequence was computer generated, and allocations were obtained by telephone from a designated coordinator who was not involved in other parts of the trial.
Blinding	Participants and treating clinicians were not masked to treatment assignment. Examinations of best corrected visual acuity (BCVA), intraocular pressure, visual field (central 24 degree tested with a 54-point grid), fundus photography, optical coherence tomography (OCT) and fluorescein fundus angiography (FFA) were carried out by masked certified personnel with a standardized procedure. Evaluation of images of fundus photography, OCT and FFA were performed by trained readers at a central reading center who were blinded to the treatment group.

Reporting for specific materials, systems and methods

We require information from authors about some types of materials, experimental systems and methods used in many studies. Here, indicate whether each material, system or method listed is relevant to your study. If you are not sure if a list item applies to your research, read the appropriate section before selecting a response.

Materials & experimental systems

n/a	Involved in the study
☒	☐ Antibodies
☒	☐ Eukaryotic cell lines
☒	☐ Palaeontology and archaeology
☒	☐ Animals and other organisms
☐	☒ Clinical data
☒	☐ Dual use research of concern

Methods

n/a	Involved in the study
☒	☐ ChIP-seq
☒	☐ Flow cytometry
☒	☐ MRI-based neuroimaging

Clinical data

Policy information about clinical studies

All manuscripts should comply with the ICMJE guidelines for publication of clinical research and a completed CONSORT checklist must be included with all submissions.

Clinical trial registration: This trial was registered with Chinese Clinical Trial Register, ChiCTR2100043061.

Study protocol: The trial protocol and statistical analysis plan are provided with the paper as part of the supplementary appendix.

Data collection: Data were collected between February, 2021 and July, 2022 at the First Affiliated Hospital of Chongqing Medical University.

Outcomes: The primary outcome was the change from baseline in best corrected visual acuity (BCVA) Early Treatment Diabetic Retinopathy Study (ETDRS) score at week 26. BCVA was measured with the letter score (range: 0 to 100, greater values with better visual acuity) of the ETDRS acuity chart read by the patient at a starting distance of 4 meters, with a repetition at 1 meter if necessary.

Secondary outcomes included the proportion of patients achieving an improvement in ETDRS score by 15 letters or greater at week 26, the proportion of patients achieving an inactive uveitis in both eyes at week 26, the changes from baseline to week 26 in visual field indices, including visual field index, mean deviation and pattern standard deviation, the resolution of retinal detachment detected by optical coherence tomography (OCT) at week 26, the change from baseline in central macular thickness on OCT at week 26, the proportion of patients initially assigned to cyclosporine therapy who received step-up treatment, and the changes from baseline in European Quality of Life-5 Dimensions (EQ-5D) score and Visual Functioning Questionnaire-25 (VFQ-25) composite score at week 26. Safety outcomes included frequency, severity and relatedness of adverse events through week 26, adjudicated by independent safety monitors.
